# The histone methyltransferase complex subunit RBBP5 serves as a central epigenetic regulator of proteostasis dynamics

**DOI:** 10.1371/journal.pbio.3004048

**Published:** 2026-10-08

**Authors:** Syeda Kubra, Michelle Sun, William Dion, Yu Bian, Ahmet Catak, Hannah Luong, Haokun Wang, Yinghong Pan, Jia-Jun Liu, Aishwarya Ponna, Lijun Liu, Ian Sipula, Jian-Hua Luo, Michael J. Jurczak, Silvia Liu, Bokai Zhu

**Affiliations:** 1 Aging Institute of UPMC, University of Pittsburgh School of Medicine, Pittsburgh, Pennsylvania, United States of America; 2 UPMC Genome Center, UPMC, Pittsburgh, Pennsylvania, United States of America; 3 Pittsburgh Liver Research Center, University of Pittsburgh, Pittsburgh, Pennsylvania, United States of America; 4 Organ Pathobiology and Therapeutics Institute, University of Pittsburgh School of Medicine, Pittsburgh, Pennsylvania, United States of America; 5 Department of Pathology, University of Pittsburgh School of Medicine, Pittsburgh, Pennsylvania, United States of America; 6 Division of Endocrinology and Metabolism, Department of Medicine, University of Pittsburgh School of Medicine, Pittsburgh, Pennsylvania, United States of America; 7 Center for Metabolism and Mitochondrial Medicine, University of Pittsburgh School of Medicine, Pittsburgh, Pennsylvania, United States of America; 8 Department of Pharmacology and Chemical Biology, University of Pittsburgh School of Medicine, Pittsburgh, Pennsylvania, United States of America; Charité - Universitätsmedizin Berlin, GERMANY

## Abstract

Proteostasis is essential for cellular health, with its disruption contributing to aging, neurodegeneration, and metabolic disorders. While the upstream proteotoxic stress-sensing and protein-folding mechanisms in the ER and cytosol are well studied, the transcriptional regulation of proteostasis remains incompletely understood, particularly concerning the temporal epigenome dynamics, chromatin landscapes, and co-regulatory networks underlying dynamic proteostasis control. Traditionally, proteotoxic stress responses were viewed as acute reactions to noxious stimuli, but recent evidence shows that many proteostasis genes exhibit ~12-hour ultradian rhythms under physiological conditions, driven by an XBP1s-dependent oscillator independent of the canonical circadian clock and cell cycle. By mapping the chromatin landscape of the murine 12-hour oscillator, we identified RBBP5—an essential subunit of the COMPASS complex responsible for H3K4 trimethylation—as a pivotal epigenetic regulator of proteostasis dynamics. In contrast, histone acetyltransferases and H3K9 acetylation were dispensable for dynamic proteostasis gene expression. RBBP5 is not only indispensable for the 12-hour oscillator but also essential for the transcriptional regulation of diverse proteotoxic stresses response, by coactivating XBP1s and promoting H3K4me3 deposition at the promoters of proteostasis genes. As a result, loss of RBBP5 sensitizes cells to proteotoxic stress in part due to impaired autophagy. Proximity labeling of H3K4me3 further uncovered a dynamic chromatin-associated proteomic architecture, including components of COMPASS, the Integrator complex and SWI/SNF remodelers, that constitutes the transcriptional response to proteotoxic stress. Together, these findings establish RBBP5 as a central regulator of proteostasis dynamics, essential for maintaining cellular resilience.

## Abstract 2

Proteostasis is essential for cellular health, but the epigenetic mechanisms regulating dynamic proteostasis programs are poorly understood. This study shows that RBBP5, a subunit of the COMPASS histone methyltransferase complex, controls ultradian proteostasis gene expression by promoting H3K4me3 deposition at the promoters of proteostasis genes.

## Introduction

Proteostasis, the maintenance of proper protein folding, trafficking, and turnover, is a major challenge for the cell [1]. Much of the cellular energy expenditure is dedicated to maintaining a healthy proteome, as millions of molecules are produced every single minute [2]. Challenges to proteostasis, exemplified by the accumulation of unfolded or misfolded proteins, can lead to a range of cellular dysfunctions and pathological conditions, such as aging, neurodegenerative diseases, cancer, and metabolic disorders [1,3–6]. To cope with proteotoxic stress, organisms have developed a wide range of stress response mechanisms, including the heat shock response, the unfolded protein response (UPR), and the more generalized integrated stress response (ISR) [7–9]. Although the detailed signaling molecules vary, these pathways generally involve transmitting the information of cellular stress to the nucleus by activating various transcription factors (TF) containing the basic leucine zipper domain (bZIP), such as spliced form of XBP1 (XBP1s), ATF4 and ATF6 and heat shock factor 1 (HSF1). After binding the stress response element in the promoters/enhancers, these TFs in turn up-regulate a plethora of genes aimed to restore proteostasis [10–12].

While proteotoxic stress responses have traditionally been studied as discrete, acute reactions to specific “noxious” stimuli, emerging evidence suggests that, even under physiological conditions in the absence of overt external stress, a large cohort of endoplasmic reticulum (ER) and proteostasis-related genes exhibits cell-autonomous ultradian oscillations with an approximate 12 h periodicity across multiple mammalian cell types and tissues, including humans [13–35]. These genes span both functional effectors—such as those involved in lipid metabolism, ER/Golgi protein processing, redox homeostasis, protein folding, ER-associated degradation (ERAD), and autophagy—as well as key upstream regulators and transcription factors, including HSF1, XBP1s, ATF4, ATF6, DDIT3, PERK, and IRE1α [15,28]*.*

Although the mechanisms governing these ~12 h rhythms remain an active area of investigation, three nonmutually exclusive models have been proposed [21,22,32,36]: (i) non–cell-autonomous regulation driven by interactions between the circadian clock and environmental cues [31,33]; (ii) emergence of cell-autonomous 12 h rhythms from two antiphase circadian transcriptional programs [29]; and (iii) the existence of a dedicated, cell-autonomous 12 h oscillator [30]. Supporting the last model, we and others have provided strong evidence that many ~12 h rhythms are driven by the UPR transcription factor XBP1s, independently of both the canonical BMAL1-dependent circadian clock and the cell cycle [14,16,17,22,25,30,37].

From an evolutionary perspective, the mammalian 12 h oscillator has been proposed to originate from ancestral circatidal clocks in marine organisms, subsequently co-opted in terrestrial animals to anticipate and adapt to recurring metabolic stress peaks at dawn and dusk [32]. Functionally, the physiological relevance of these rhythms is most extensively characterized in the mouse liver, where integrative multi-omics analyses coupled with kinetic mathematical modeling reveal that a substantial fraction of hepatic metabolic processes—particularly those governing lipid and carbohydrate metabolism—exhibit dominant ~12 h rhythmicity rather than classical ~24 h circadian oscillations [20]. These findings underscore a tight functional coupling between the 12 h UPR program and core metabolic regulation. Consistent with this framework, genetic disruption of hepatic 12 h rhythms accelerate liver aging, leading to impaired proteostasis, dysregulated lipid metabolism, and mitochondrial dysfunction [14,16,25]. Collectively, these observations position the 12 h ultradian rhythm as a central integrator of proteotoxic stress signaling. As such, it offers a conceptual and experimental framework for studying proteostasis at a system level—shifting the paradigm from isolated stress pathways toward a unified, temporally coordinated regulatory network.

While significant work has been done on upstream proteotoxic stress sensing and protein-folding mechanisms in the ER and cytosol, there remains a lack of fundamental understanding of the transcriptional regulation of proteostasis in general, particularly concerning the temporal epigenome dynamics, chromatin landscapes, and co-regulatory networks underlying global proteostasis control. In this study, we seek to address an important unanswered question: is there a designated epigenetic regulator that orchestrates global proteostasis dynamics? By analyzing the chromatin landscape of the murine 12 h hepatic oscillator, we identified RBBP5—an essential subunit of the Complex Proteins Associated with Set1 (COMPASS) complex responsible for depositing H3K4me3 [38]—as a pivotal epigenetic regulator that governs global proteostasis dynamics. By contrast, histone acetyltransferase (HAT) and associated H3K9Ac are dispensable for dynamic proteostasis gene expression. RBBP5 plays a critical role in regulating both the 12 h oscillator and transcriptional response to a plethora of acute proteotoxic stress, functioning as a co-activator for XBP1s, to mark H3K4me3 at the promoter of proteostasis genes. Loss of RBBP5 leads to heightened susceptibility to proteotoxic stress, impairing autophagy and reducing cell survival in vitro. In human liver, RBBP5 expression is also positively correlated with genes involved in ribosome biogenesis, translation, and mRNA processing. Proximity labeling of H3K4me3 further reveals the chromatin-associated proteomic architecture during proteotoxic stress, including subunits of the COMPASS, the Integrator complex and SWI/SNF chromatin remodelers. Our findings thus establish RBBP5 as a central regulator of proteostasis dynamics, essential for cellular resilience and organismal health.

## Results

### 12 h RBBP5 chromatin binding is concomitant with the promoter-proximal ~12 h H3K4me3 oscillation in mouse liver

The 12 h oscillator is transcriptionally regulated by the UPR TF XBP1s in mouse liver [14,17,30], and likely under control by additional bZIP TFs, such as ATF4 and ATF6 [23]. However, decades of research on eukaryotic gene regulation demonstrated that TFs themselves are not sufficient to drive transcription initiation and elongation, and additional so-called “co-regulators” are required for full gene activation by shaping the epigenetic landscape [39–41]. Currently, the epigenetic landscape of both the 12 h oscillator and proteotoxic stress responses is poorly characterized. To identify epigenetic regulators of global proteostasis control, we first examined a published temporal hepatic epigenome dataset for histone modifications associated with the active promoters of ~12 h genes [42]. We found ~12 h rhythms of histone H3 trimethylated at lysine 4 (H3K4me3) (*P* = 0.024 by the cosinor2 method [43]), but not two histone acetylation markers, histone H3 acetylated at lysine 9 (H3K9Ac) and lysine 27 (H3K27Ac), at the promoters of ~12 h genes (Fig 1A and 1B). This is particularly prominent for those ~12 h genes with rhythmic expressions peaking at ZT0 and ZT12 that are enriched for proteostasis pathways [17], with H3K4me3 levels also peaking at these two time points (Fig 1A and 1B).

**Fig 1:**
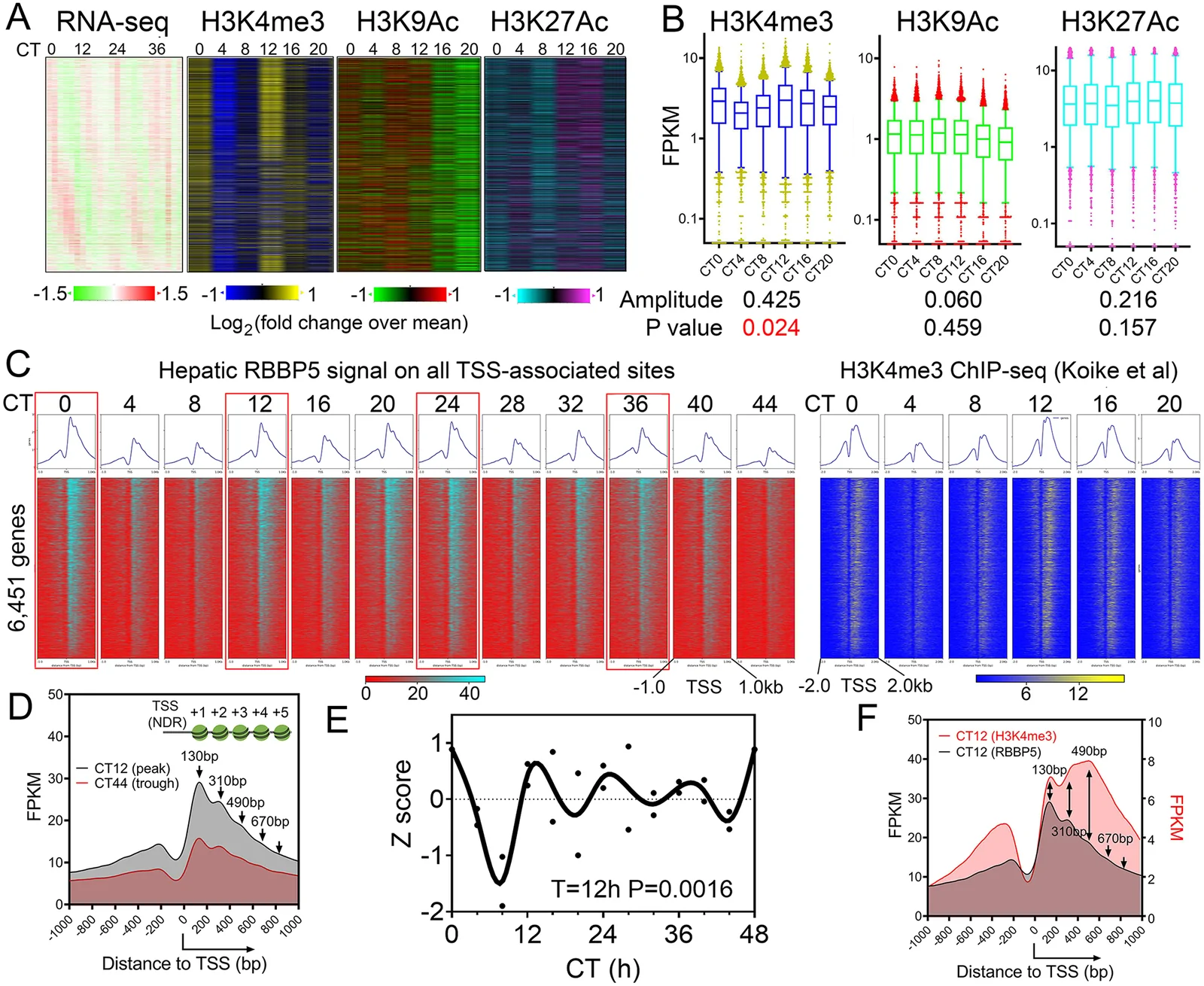
Global ~12 h RBBP5 cistrome is associated with promoter-proximal ~12 h H3K4me3 epigenome in mouse liver. **(A)** Heatmap of ~12 h hepatic transcriptome aligned with H3K4me3, H3K9Ac, and H3K27Ac cistromes. **(B)** Box and whiskers plot (5 ~ 95 percentile) of the quantification of temporal H3K4me3, H3K9ac, and H3K27Ac oscillations shown in A (*n* = 4,509 sites for each mark). Amplitude and p values for having 12 h rhythm for each of the epigenetic mark were calculated by the cosinor2 package [43] using the average FKPM value. **(C)** Heatmap showing RBBP5 and H3K4me3 chromatin occupancy 1kb ± of TSS of 6,451 genes. **(D)** RBBP5 chromatin occupancy at CT12 and CT44 aligned with nucleosome positioning. **(E)** Quantification of temporal average RBBP5 binding intensity (Z score normalized) (*n* = 2 independent samples at each time point). **(F)** RBBP5 chromatin occupancy aligned with H3K4me3 occupancy at CT12. All data were collected from mice housed under constant darkness condition with different times indicated by CT. Numerical values are available in S1 Data.

In eukaryotes, H3K4 is tri-methylated by the COMPASS complex, which includes regulatory subunits RBBP5, ASH2L, WDR5, DPY30, and catalytic subunits SETD1A/B/MLL1-4 (S1A Fig) [44–46]. Via writing H3K4me3, COMPASS is essential for the eviction of paused RNA polymerase II (RNAPII) and promotes transcriptional elongation [47,48]. Landscape In Silico deletion Analysis (LISA) [49] further inferred COMPASS subunits WDR5, ASH2L, DYP30, and RBBP5 as putative epigenetic regulators of hepatic ~12 h, but not circadian genes (S1B Fig). Being the commonly shared regulatory subunits for all MLL family H3K4 methyl transferase complex (including SETD1A/B and MLL1-4), RBBP5/ASH2L forms the minimal heterodimer sufficient to activate all MLL histone methyl transferase [50,51] and is essential for the assembly of the whole COMPASS complex [46,52]. Between the two essential subunits, we initially focused on RBBP5. Eigenvalue/pencil method [53] revealed that the levels of both hepatic *Rbbp5* mRNA and nuclear RBBP5 protein exhibit ~12 h oscillations, with the nuclear protein level peaking at ~CT0 and ~CT12 (S1C and S1D Fig). These phases closely align with the acrophases of H3K4me3 and rhythmic gene expression (Fig 1A and 1B), supporting coordinated temporal regulation. The ~12 h oscillation of nuclear RBBP5 was further validated by four independent statistical approaches (S1D Fig), underscoring the robustness of this rhythmic pattern. Beyond the liver, pronounced ~12 h rhythms in total RBBP5 protein were also observed across multiple mice tissues—including heart, inguinal white adipose tissue (iWAT), kidney, and several brain regions (cortex, hypothalamus, and cerebellum)—with peak expression consistently occurring at CT10–14 and CT22–26 in all tissues except iWAT (S1E and S1F Fig) [54]. Together, these findings suggest that RBBP5 may be a conserved component of the mammalian ~12 h oscillator.

To further evaluate the potential role of RBBP5 in establishing the epigenetic landscape of hepatic ~12 h rhythms, we performed temporal RBBP5 ChIP-seq at a 4-h interval for a total of 48 h in the liver of male C57BL/6 mice housed under constant darkness condition fed *ad libitum*. Importantly, two fully independent ChIP-seq experiments were conducted using separate biological samples, with chromatin immunoprecipitation, library preparation, and sequencing carried out independently for each run. Chromatin occupancies of RBBP5 align well with nucleosome positioning downstream of the transcription start sites (TSS) and the H3K4me3 epigenome, with ‘ridges’ corresponding to 130 bp, 310 bp, 490 bp, 670 bp downstream of TSS at all time points, thus demonstrating the high quality of our ChIP-seq dataset (Fig 1C–1F). In total, we identified 7,963 hepatic RBBP5 binding sites that are within 2kb (up and downstream) of TSS of 6,451 genes (Fig 1C and S1 Table). The average binding intensities of RBBP5 displayed a robust 12 h rhythm (*P* = 0.0016 with RAIN [55]), with peaks occurring at CT0, CT12, CT24, and CT36 (Figs 1C–1E and S2A). This 12 h rhythm aligns with the 12 h oscillation of promoter-proximal H3K4me3 (Fig 1C–1F), but not with those of H3K9Ac or H3K27Ac (S2B Fig). This pattern is exemplified by RBBP5 promoter binding to *Xbp1*, one of the major UPR TFs and the transcriptional regulator of the 12 h oscillator, and to *Hsph1*, a canonical 12 h oscillator output gene encoding a heat shock protein (S2C Fig). Using the RAIN [55] algorithm, we identified a total of 4,667 (adjP < 0.05) RBBP5 binding sites that cycle with a ~12 h period (S2D**–**S2F Fig and S1 Table). Collectively, these results establish the epigenetic landscape of hepatic 12 h oscillator is marked by RBBP5-H3K4me3.

### Hepatic RBBP5 cistrome coincides with that of XBP1s and hepatic ~12 h transcriptome

To investigate if RBBP5 regulates hepatic ~12 h rhythms of gene expression, we first examined the correlation between RBBP5 proximal promoter binding status and ~12 h rhythms of gene expression. As illustrated in Fig 2A, genes with proximal promoter RBBP5 binding are enriched for ~12 h rhythms of gene expression (*P* = 3.46e−66 by Chi-squared test). In addition, RBBP5 chromatin binding exhibits more robust ~12 h rhythmicity at sites associated with 12 h cycling genes compared to sites linked to genes lacking ~12 h rhythmicity (Fig 2B–2D). For those 2,527 ~12 h genes with RBBP5 binding, the phases of RBBP5 chromatin binding precede the phases of gene expression (*P* < 10e−15 by paired *t* test) (Fig 2B). This temporal relationship suggests that RBBP5 likely acts as a driver, rather than a consequence of ~12 h rhythms of gene expression.

**Fig 2:**
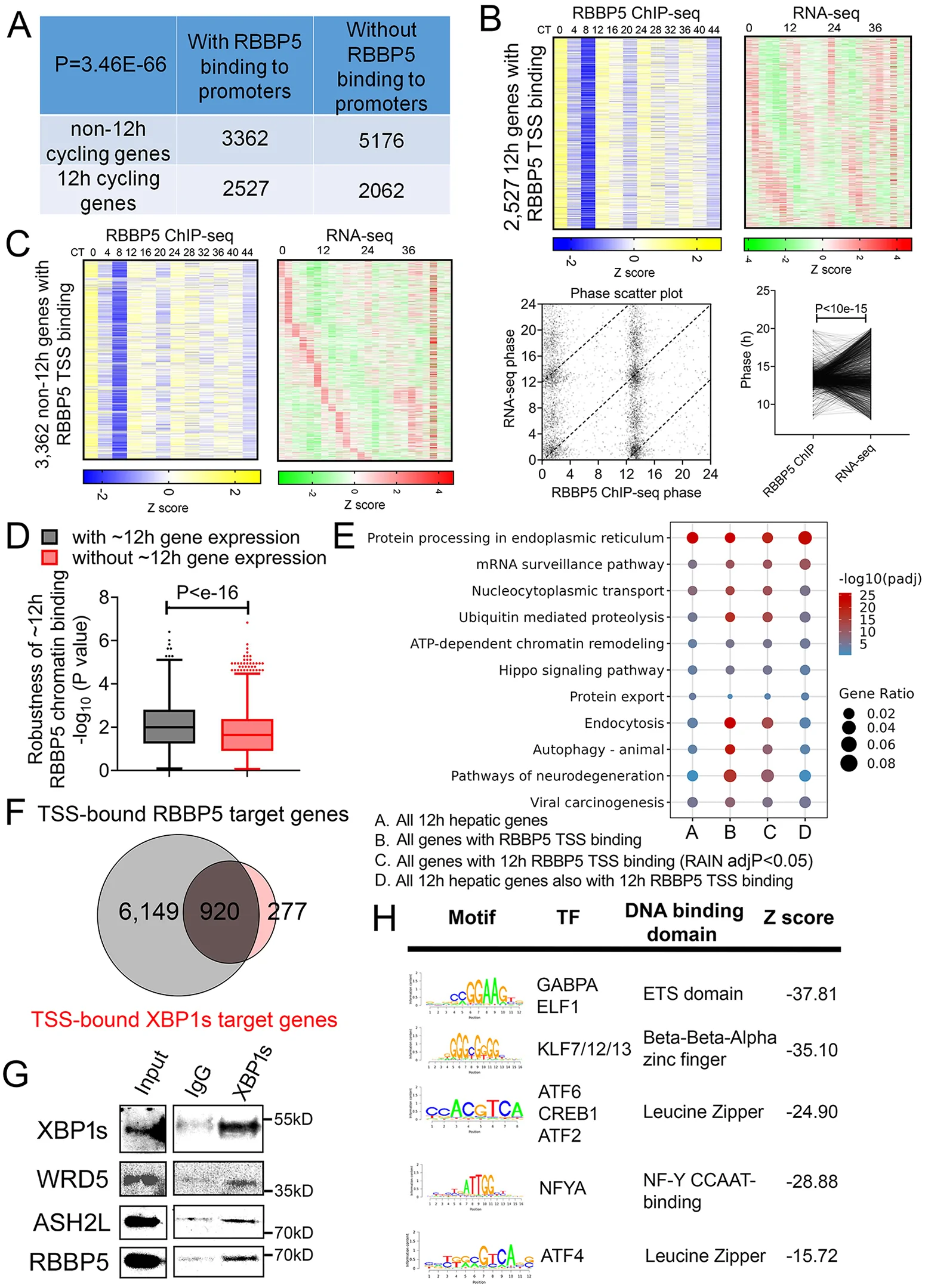
Hepatic RBBP5 cistrome coincides with that of XBP1s and hepatic ~12 h transcriptome. **(A)** The table shows the number of genes with or without ~12 h rhythms and with or without promoter RBBP5 binding. *P* value is calculated by Chi-squared test. **(B)** Heatmap showing RBBP5 binding intensity and ~12 h rhythms of gene expression on 2,527 genes (top). Scatter and bar plot demonstrating the phase relationship between temporal ~12 h RBBP5 chromatin recruitment and the hepatic ~12 h gene expression (bottom). **(C)** Heatmap showing RBBP5 binding intensity and gene expression on 3,362 genes that do not exhibit ~12 h rhythms of gene expression. **(D)** Box-whisker plot comparing robustness (calculated by negative log _10_ *P* value) of ~12 h RBBP5 chromatin binding rhythmicity on genes with or without ~12 h rhythmic expression. **(E)** Dot plot for GO analysis demonstrating the −log_10_ transformed adjP value and gene counts of different enriched pathways for different genes. **(F)** Venn diagram demonstrating distinct and common cistromes for RBBP5 and XBP1s. **(G)** western blot showing co-IP in liver nuclear extracts from CT0 using anti-XBP1s and normal IgG control antibody. **(H)** Motif analysis of RBBP5 binding sites of 6,149 genes. All data were collected from mice housed under constant darkness condition with different times indicated by CT. Two-tailed paired Student *T* test was used for B. Two-tailed unpaired Welch’s *t* test was used for D. Numerical values are available in S2 Data.

We next performed Gene Ontology (GO) analysis on those hepatic genes with proximal promoter RBBP5 binding and revealed strong enrichment of proteostasis pathways including protein processing in the ER and ubiquitin-mediated proteolysis (Figs 2E and S3). Additionally, pathways involved in mRNA processing, such as mRNA splicing and mRNA surveillance, were also enriched (Figs 2E and S3). These findings align with previous observations that both mRNA and protein metabolism pathways are enriched in genes exhibiting ~12 h rhythms in both mice and humans [18,25,56]. The coupling of mRNA and protein metabolism is orchestrated by a SON-XBP1s axis and involves the liquid-liquid phase separation (LLPS) dynamics of nuclear speckles [25]. Similar pathways are also enriched in genes exhibiting both ~12 h rhythms of RBBP5 promoter binding and gene expression (Figs 2E and S3).

XBP1s is a major transcriptional factor regulating the hepatic 12 h oscillator and also exhibits a global 12 h rhythm of chromatin binding to the promoter regions peaking at CT0, 12, 24, and 36 [14,17]. Comparing the cistromes of RBBP5 and XBP1s revealed a significant overlap between the two, with 920 genes sharing RBBP5 and XBP1s proximal promoter binding (Fig 2F). Co-immunoprecipitation in the hepatic nuclear extracts at CT0 confirmed the physical interaction between XBP1s and subunits of SET1/COMPASS, including RBBP5, ASH2L, and WDR5 (Fig 2G). Motif analysis of the DNA sequences around the 6,149 RBBP5 binding sites that do not overlap with XBP1s cistrome identified binding motifs for GABPA, KLF, ATF6, NFYA, and ATF4 (Fig 2H). These findings suggest that, in addition to acting as a co-activator for XBP1s, RBBP5 may also work with other transcription factors to shape the hepatic ~12 h epigenome.

### RBBP5 is an epigenetic regulator of the hepatic ~12 h oscillator, but not the BMAL1-dependent canonical core circadian clock

To establish the causality between RBBP5 and hepatic 12 h rhythms of gene expression, we generated RBBP5 liver hepatocyte-specific knockout (RBBP5 ^*LKO*^) mice using the CRE-loxP system. Exon 10 of mouse *Rbbp5,* which encodes the SET/ASH2L binding domain, was flanked by loxP sites (Fig 3A). CRE-mediated deletion of Exon 10 is expected to lead to a frameshift and nonsense-mediated RNA decay of truncated *Rbbp5* transcript (S4A Fig). Crossing homozygous floxed *Rbbp5* mice with Albumin-CRE mice resulted in liver hepatocyte-specific deletion of RBBP5, which was confirmed by DNA genotyping, immunofluorescence (IF) against RBBP5 and western blot analysis (Figs 3B and S4B–S4D). The resulting RBBP5 ^*LKO*^ mice weighed slightly less than wild-type counterparts (S5A Fig) but maintained normal rhythmic locomotor activity and fasting-feeding cycles under both 12 h:12 h light/dark and constant darkness conditions (S5B–S5I Fig).

**Fig 3:**
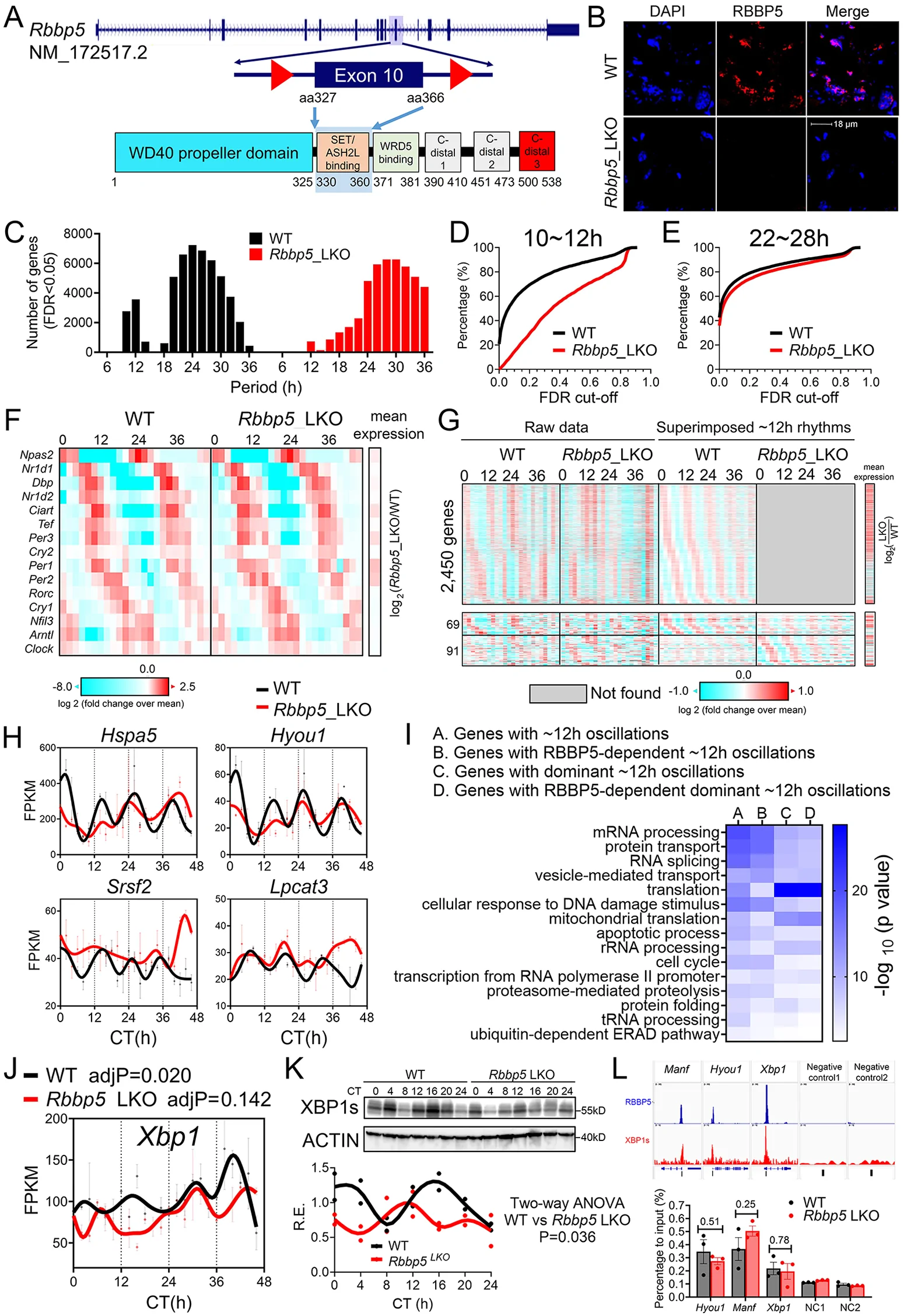
RBBP5 is an epigenetic regulator of the hepatic ~12 h ~ 24 h circadian clock. **(A)** Schematic of the design of *Rbbp5*^*Flox*^ mice. **(B)** Immunofluorescence of RBBP5 in the liver of *Rbbp5* ^*Flox*^ and *Rbbp5* ^*LKO*^ mice. **(C)** Histograms show the period distributions of all rhythmic genes uncovered from *Rbbp5* ^*Flox*^ and *Rbbp5* ^*LKO*^ mice (with adjP values or FDR < 0.05). **(D, E)** Cumulative distribution of 10~12 h (D) and 22~28 h (E) genes in *Rbbp5* ^*Flox*^ and *Rbbp5* ^*LKO*^ mice with different FDR cut-offs. **(F)** Heat map of 15 core circadian clock gene expression in the liver of *Rbbp5* ^*Flox*^ and *Rbbp5* ^*LKO*^ mice. Log_2_ normalized fold change of average gene expression across 48 hours between *Rbbp5* ^*Flox*^ and *Rbbp5* ^*LKO*^ mice was also shown. **(G)** Heat map of dominant ~12 h gene expression (or lack thereof) in *Rbbp5* ^*Flox*^ and *Rbbp5* ^*LKO*^ mice with both raw data and superimposed ~12 h rhythms shown. 2,450 ~12 h genes were abolished, 69 ~12 h genes dampened, and 91 ~12 h genes were enhanced in *Rbbp5* ^*LKO*^ mice, respectively. Log_2_ normalized fold change of average gene expression across 48 hours between *Rbbp5* ^*Flox*^ and *Rbbp5* ^*LKO*^ mice was also shown. **(H)** RNA-Seq data for representative RBBP5-dependent ~12 h-cycling proteostasis (*Hspa5* and *Hyou1*), mRNA processing (*Srsf2*) and lipid metabolism (*Lpcat3*) genes in the liver of *Rbbp5* ^*Flox*^ and *Rbbp5* ^*LKO*^ mice (*n* = 2 biologically independent samples per time point). **(I)** Heat map summary of GO analysis demonstrating the −log_10_ transformed P values of different enriched pathways for different genes. **(J)** Temporal expression profile of *Xbp1s* mRNA in livers of *Rbbp5* ^*Flox*^ (WT) and *Rbbp5* ^*LKO*^ mice over 48 h, as determined by RNA-seq. Adjusted *P* values calculated with RAIN for ~12-h rhythmicity are indicated. **(K)** Representative immunoblot and quantification of hepatic XBP1s protein abundance in *Rbbp5* ^*Flox*^ (WT) and *Rbbp5* ^*LKO*^ mice over 24 h (*n* = 2 biological replicates). ACTIN was used as a loading control. R.E., relative expression. Statistical significance between genotypes was determined by two-way ANOVA. **(L)** XBP1s ChIP-qPCR at CT12 in livers of *Rbbp5* ^*Flox*^ (WT) and *Rbbp5* ^*LKO*^ mice (*n* = 3 biological replicates). Top, representative genome-browser tracks showing RBBP5 and XBP1s occupancy at the indicated loci. The vertical bars below indicate position of each ChIP-qPCR amplicon. Bottom, XBP1s enrichment at the promoters of the canonical 12 h UPR genes *Hyou1*, *Manf*, and *Xbp1*, with two negative-control regions (NC1 and NC2). Two-tailed Welch’s *t* test were used. Data: Mean ± S.E.M. All data were collected from mice housed under constant darkness condition with different times indicated by CT. Numerical values are available in S3 Data.

To identify the RBBP5-dependent oscillating hepatic transcriptome, we performed bulk RNA sequencing (RNA-Seq) analysis in the liver of male *Rbbp5* ^*Flox*^ and *Rbbp5* ^*LKO*^ mice at 2-h intervals for a total of 48 h under constant darkness with biological duplicates (S2 Table). To identify genes cycling with a period ranging from 6 to 36 h, we initially applied the RAIN algorithm [55] to each genotype’s temporal transcriptome [55] (S3 Table). Compared to alternative methods like JTK_CYCLE, RAIN detects rhythms with arbitrary waveforms and therefore more robustly uncovers ultradian rhythms [17]. Consistent with past studies [16,17,30,33,35], two populations of hepatic transcripts cycling with periods of ~12 h and ~24 h were identified in *Rbbp5* ^*Flox*^ mice liver (Fig 3C–3E). By contrast, in *Rbbp5* ^*LKO*^ mice, the ~12 h rhythms are all but abolished and the circadian oscillations remain largely intact, which include all core circadian clock genes (such as *Bmal1*, *Per1/2/3*, and *Nr1d1/2*) displaying robust circadian rhythms with the same phases and amplitudes as those observed in *Rbbp5* ^*Flox*^ mice (Fig 3C–3F). Specifically, using RAIN with an FDR cutoff of 0.01 and 0.05, we identified a total of 3,549 and 6,025 hepatic transcripts cycling at periods between 10 and 12 h in *Rbbp5* ^*Flox*^ mice, respectively. By contrast, only 61 and 813 10–12 h genes were observed in *Rbbp5* ^*LKO*^ mice with the same FDR thresholds, indicating over 93% ~12 h hepatic transcriptome was abolished with hepatocyte RBBP5 ablation (S6A and S6B Fig and S3 Table). GO analysis confirmed that these RBBP5-depenent ~12 h hepatic transcriptomes are enriched in mRNA processing and proteostasis pathways, distinct from those of RBBP5-independent circadian genes enriched in lipid metabolism and phosphorylation (S6C Fig).

To assess the robustness of our findings across analytical approaches, we applied multiple orthogonal methods, including MetaCycle [57], cosinor2 [43], and the eigenvalue/pencil method [53]. While MetaCycle and cosinor2 are less sensitive than RAIN for detecting ultradian rhythms with nonsinusoidal or asymmetric waveforms, both methods nonetheless consistently revealed a marked depletion of ~12 h gene expression rhythms in *Rbbp5* ^*LKO*^ mice (S7A–S7H Fig). In contrast to these approaches, the eigenvalue/pencil method does not require pre-specification of period ranges, allowing unbiased decomposition of complex temporal signals into multiple superimposed oscillatory components [53]. Revealing up to three oscillations from each gene, eigenvalue/pencil analyses also revealed prevalent ~24 h and ~12 h oscillations in *Rbbp5* ^*Flox*^ mice, along with a third population cycling at a period around 8 h (S4 Table) (S8A and S8B Fig). Hepatic ~8 h oscillations were also previously reported, but their regulation and function remained elusive [28,33]. *Rbbp5* ^*LKO*^ mice, on the other hand, exhibited a drastically different spectrum, characterized by the near complete loss of the ~12 h rhythms, de novo gains of many faster ultradian rhythms with periods between 6 and 7 h, and the maintenance of most circadian rhythms (S8A–S8C Fig). Similar findings were also observed for dominant oscillations (which is the one with the largest amplitude among the three superimposed oscillations for each gene) for each gene (Fig 3G and 3H). For the ~12 h genes originally identified in *Rbbp5* ^*Flox*^ mice but lost in *Rbbp5* ^*LKO*^ mice, the period spectrum in the *Rbbp5* ^*LKO*^ mice displayed a diverse distribution. This ranged from a complete lack of rhythmicity (~39%) to shorter ultradian periods of 6–7 h, and even to longer periods exceeding 24 h (S8D Fig). RBBP5-dependent ~12 h transcriptome uncovered by the eigenvalue/pencil showed substantial overlap with the results obtained via the RAIN method. This convergence was evident both in the specific genes identified (S8E Fig) and in the enriched biological pathways (Figs 3I and S6C), thereby confirming the robustness of our findings.

Lastly, since XBP1s is a major transcriptional regulator of the hepatic 12 h oscillator [14,17], we examined how RBBP5 regulates its temporal expression. In the liver of *Rbbp5* ^*LKO*^ mice, the 12 h oscillations of *Xbp1s* mRNA and protein were impaired, accompanied by a reduction in mean daily XBP1s expression (Fig 3J and 3K). We next performed XBP1s ChIP-qPCR at CT12, when hepatic XBP1s protein levels were comparable between WT and *Rbbp5* ^*LKO*^ mice. Hepatic XBP1s occupancy at the promoters of canonical 12 h UPR genes was comparable between the two genotypes (Fig 3L). These results suggest that RBBP5 regulates the temporal expression of XBP1s but is likely not required for XBP1s chromatin recruitment, consistent with its role as a transcriptional co-regulator. Together, these results thus establish RBBP5 as a central epigenetic regulator of the hepatic 12 h oscillator, with more than 90% ~12 h hepatic transcriptome abolished in its absence.

In addition to the liver, cell-autonomous ~12 h rhythms of proteostasis gene expression can also be observed in mouse embryonic fibroblasts (MEFs) synchronized by low concentration of ER stress inducer tunicamycin (Tu) [30]. We observed a ~12 h rhythm of *Rbbp5* expression in Tu-synchronized MEFs, which is independent of BMAL1 but require XBP1s (S9A Fig), indicating RBBP5 itself is also under 12 h oscillator control. To test the functional role of COMPASS in regulating the cell-autonomous ~12 h rhythms, we knocked down either *Rbbp5* or *Ash2l* via siRNA in a previously described 12 h oscillator reporter MEFs that stably express *Manf* (*Manf* is an XBP1s-dependent 12 h oscillator output gene) promoter-driven destabilized luciferase (dluc) [17], and found that both siRNAs significantly dampen the ~12 h oscillation of luciferase activity as well as shorten the period (S9B Fig). By contrast, neither *RBBP5* nor *ASH2L* knocking down affects the circadian oscillation of *Bmal1* promoter activity in human U2OS cells in a previous genome-wide siRNA screen study [58] (S9C Fig). Collectively, our results demonstrate that RBBP5 is an integral component of the 12 h oscillator, but dispensable for the canonical circadian clock both in vivo and in vitro.

### RBBP5 modulates the hepatic transcriptional response to acute proteotoxic stress in vivo

In contrast to the self-sustained oscillatory dynamics of the UPR, its regulation has been more extensively characterized as a transient response to proteotoxic stress, most notably during ER stress [12]. Upon accumulation of misfolded proteins in the ER, the UPR is activated through signaling pathways that relay information from the ER to the nucleus, culminating in the activation of three principal transcription factors: XBP1s, ATF4, and ATF6. These factors coordinate an adaptive transcriptional program that upregulates proteostasis-related genes to restore ER homeostasis (Fig 4A) [59–61]. However, unsolved chronic ER stress can also trigger maladaptive and deleterious responses, such as the activation of pro-inflammatory and immune gene expression through the TRAF2-mediated activation of AP1 (comprised of c-Fos and c-Jun), NF-κB, or STAT3 TFs (Fig 4A) [62]. Our findings that RBBP5 can co-activate XBP1s to regulate the 12 h oscillator suggest that RBBP5 is essential for the adaptive UPR TFs-mediated activation of proteostasis genes but is not required for the maladaptive activation of immune genes by immune-regulatory TFs, whose binding motifs are absent from RBBP5 genome binding sites (Fig 2F).

**Fig 4:**
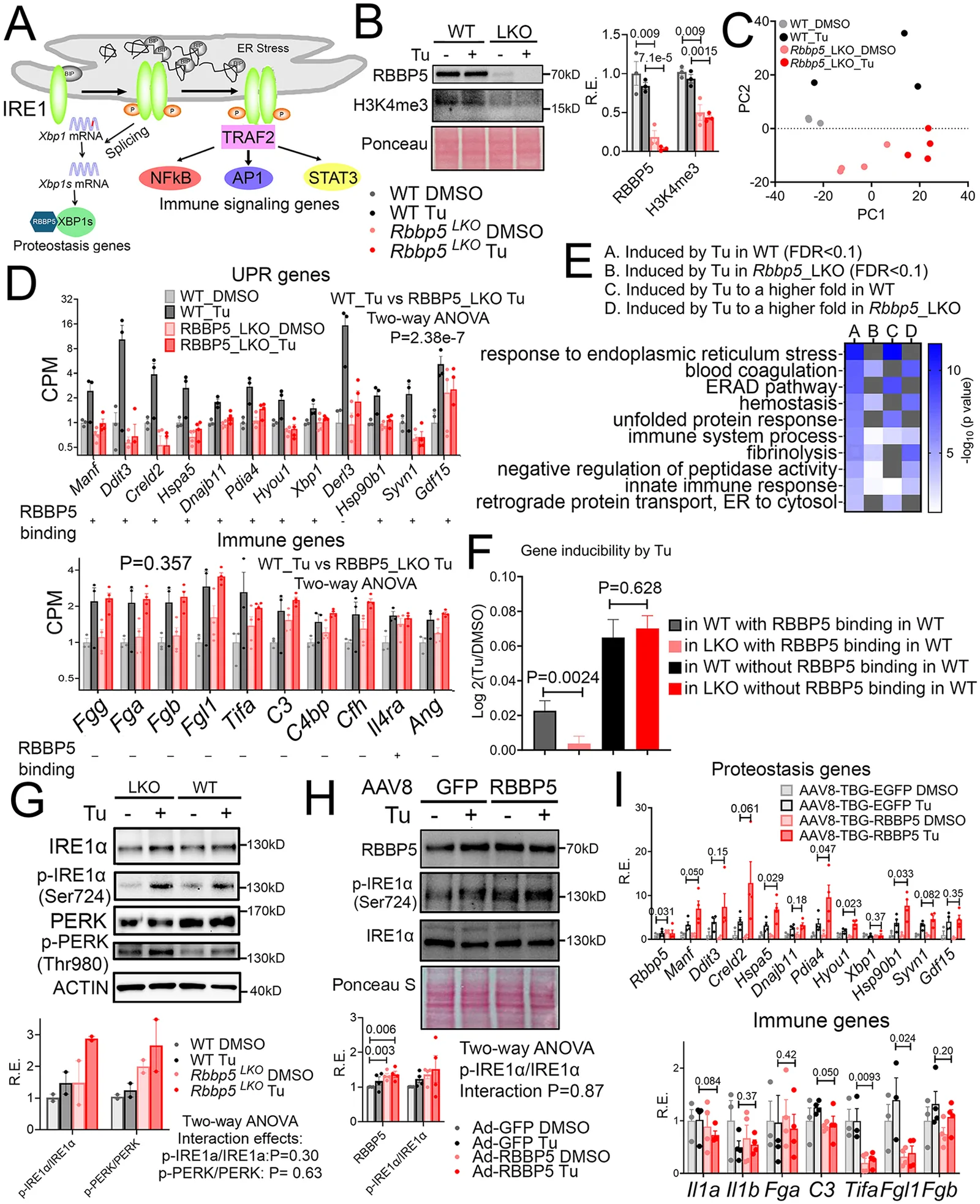
RBBP5 regulates the hepatic transcriptional response to proteotoxic stress. **(A)** The diagram of UPR signaling cascade. **(B)** western blot analysis and quantification of RBBP5 and H3K4me3 in the liver of *Rbbp5* ^*Flox*^ and *Rbbp5* ^*LKO*^ mice with or without Tu treatment (*n* = 3 biologically independent samples per group). **(C)** PCA showing the hepatic transcriptional response to Tu in *Rbbp5* ^*Flox*^ and *Rbbp5* ^*LKO*^ mice. *n* = 3 for *Rbbp5* ^*Flox*^ DMSO and *Rbbp5* ^*Flox*^ Tu, and *n* = 4 for *Rbbp5* ^*LKO*^ DMSO and *Rbbp5* ^*LKO*^ Tu. **(D)** Representative RNA-seq data for proteostasis (top) and immune genes (bottom) with RBBP5 promoter-binding status also shown below. *n* = 3 biologically independent samples for *Rbbp5* ^*Flox*^ DMSO and *Rbbp5* ^*Flox*^ Tu, and *n* = 4 biologically independent samples for *Rbbp5* ^*LKO*^ DMSO and *Rbbp5* ^*LKO*^ Tu. **(E)** Heat map summary of GO analysis demonstrating the −log_10_ transformed P values of different enriched pathways for different genes. **(F)** Log _2_ normalized fold induction by Tu in *Rbbp5* ^*Flox*^ and *Rbbp5* ^*LKO*^ mice for genes with or without RBBP5 promoter binding (*n* = 6,158 and 6,545 genes with or without RBBP5 binding, respectively). **(G)** western blot analysis and quantification of different proteins in *Rbbp5* ^*Flox*^ and *Rbbp5* ^*LKO*^ mice treated with or without Tu (*n* = 2 biologically independent samples per group). **(H, I)** Mice tail-vein injected with AAV8-TGB-GFP or AAV8-TGB-RBBP5 were treated with or without Tu. Western blot analysis and quantification (H) and qPCR (I). For western blot, *n* = 4 biologically independent samples per group. For qPCR, *n* = 3 for AAV8-TBG-EGFP DMSO, *n* = 4 for AAV8-TBG-EGFP Tu, *n* = 4 for AAV8-TGB-mRBBP5 DMSO and *n* = 4 for AAV8-TGB-mRBBP5 Tu. Data: Mean ± S.E.M. Statistical tests used: Two-way ANOVA for D, G, and H, unpaired two-tailed Student *t* test for B, F, H, and unpaired one-tailed Student *t* test for I. Numerical values are available in S4 Data.

To test this hypothesis, we intraperitoneally injected male *Rbbp5* ^*Flox*^ and *Rbbp5* ^*LKO*^ mice with vehicle control or 0.05 mg/kg Tu to induce acute ER stress and performed RNA-seq on hepatic transcriptome isolated 8 h post-injection (S5 Table). This lower dose of Tu was selected for its ability to induce the UPR, but it was significantly less toxic than the more common experimental dose of 1 mg/kg—which is known to elicit hepatocellular death and severe toxicity [63]. Western blot analysis confirmed the depletion of RBBP5 and a reduction of H3K4me3 level in the liver of *Rbbp5* ^*LKO*^ mice (Fig 4B). Principle component analysis (PCA) demonstrated the separation of the four groups on the transcriptome space (Fig 4C). Using an adjusted *p* value (FDR) of 0.10 as cut-off, we identified 139 and 45 genes significantly induced by Tu in the liver of *Rbbp5* ^*Flox*^ and *Rbbp5* ^*LKO*^ mice, respectively, with 21 commonly shared between the two (Figs 4D and S10A–S10G and S6 Table). In *Rbbp5* ^*Flox*^ mice, these 139 genes are enriched in pathways of proteostasis and immune functions as expected, with pathways of ‘response to ER stress’ and ‘ERAD’ among the top two GO terms (Fig 4D and 4E). By contrast, in *Rbbp5* ^*LKO*^ mice, these 45 genes are only enriched in immune functions, with proteostasis genes not being significantly induced by Tu (Fig 4D and 4E). In general, proteostasis genes were induced by Tu to a much greater fold in *Rbbp5* ^*Flox*^ mice (such as *Creld2*, *Xbp1*, *Hsp90b1*), while the opposite was found for blood coagulation and immune genes in *Rbbp5* ^*LKO*^ mice (such as *S100a9* and *Fgb*) (Figs 4D, 4E, S10E, S10F and S10H). Further integrating RBBP5 ChIP-seq results, we found that, on average, the ablation of RBBP5 markedly diminished the fold induction by Tu for genes with RBBP5 promoter binding, while it had no effects for those without RBBP5 chromatin recruitment (Fig 4D and 4F). To rule out the possibility that RBBP5 regulates UPR via influencing the upstream ER stressing in the ER, we performed western blot analysis of p-IRE1α and p-PERK in the liver of *Rbbp5* ^*Flox*^ and *Rbbp5* ^*LKO*^ mice and did not observe any notable difference in the increase in their phosphorylated level in response to Tu between the two genotypes (Fig 4G).

To determine if RBBP5 overexpression can also amplify UPR, we tail-vein injected AAV8-TBG-GFP and AAV8-TBG-RBBP5 viruses into wild-type male mice followed by intraperitoneal injection with vehicle control or 0.025 mg/kg Tu 30 days later. Thyroxine binding globulin (TBG) promoter is liver-specific and directs RBBP5 transgene expression in hepatocytes [64]. qPCR and western blot analysis confirmed an increase of total hepatic expression of RBBP5 of ~1.3 fold in AAV8-TBG-RBBP5 group under vehicle control condition (Fig 4H and 4I). Consequently, RBBP5 hepatic overexpression significantly amplified the UPR of proteostasis genes including *Manf*, *Hspa5* (*BiP*), *Pdia4*, *Hyou1*, and *Syvn1* without affecting the upstream ER stress sensing (Fig 4H and 4I). In addition, we found that RBBP5 overexpression attenuated the expression of several immune and blood coagulation genes such as *C3*, *Tifa* and *Fgl1* under ER stress conditions (Fig 4I).

Finally, leveraging the human gene-derived correlations across tissues (GD-CAT) dataset [65], we identified that in human liver, RBBP5 expression is also positively correlated with those involved in proteostasis (such as ribosome biogenesis, translation regulation) and mRNA metabolism (mRNA splicing and processing) and negatively associated with those implicated in immune response (acute phase response, complement activity, and immunoglobin activity) (S11 Fig). Taken together, these results demonstrate that RBBP5 modulates the transcriptional regulation of hepatic response to acute proteotoxic stress in vivo. RBBP5 activates the expression of proteostasis genes while repressing those involved in immune functions in both mice and humans.

### Loss of RBBP5 compromises the cell-autonomous transcriptional response to proteotoxic stress and nutrient-deprivation, resulting in impaired autophagy and reduced cell survival

To determine whether RBBP5 regulates transcriptional responses to proteotoxic stress in a cell-autonomous manner beyond the liver, we examined MEFs, which exhibit both a robust cell-autonomous XBP1s-dependent 12 h oscillator and strong transcriptional responses to proteotoxic stress [17,30]. *Rbbp5* mRNA expression was induced by Tu and transient knockdown of RBBP5 using siRNA significantly dampened the proteostasis gene expressions in response to Tu, mirroring the effects observed with XBP1s knockdown (S12A and S12B Fig). Similarly, knocking down ASH2L also markedly dampened the UPR, whereas knocking down SETD1A, one of the H3K4me3 methyltransferases in the SET1/COMPASS complex, had only modest effects, which were likely due to redundancy among the many H3K4me3 methyltransferases that exist (S12C Fig). As a negative control, we further knocked down both GCN5 and PCAF, proteins that function to write H3K9Ac as subunits in two distinct histone acetyltransferase (HAT) complexes, SAGA (Spt-Ada-Gcn5 acetyltransferase) and ATAC (Ada2a-containing) [66] and found it had no effects on the induction of *Xbp1* and *Manf* expression in response to ER stress (S12D Fig). The lack of effects on UPR for HAT subunits is consistent with the absence of ~12 h rhythms of both H3K9Ac and H3K27Ac levels around the promoter of ~12 h proteostasis genes in mouse liver (Fig 1A and 1B). ChIP-qPCR analysis confirmed a gradual, temporal increase in the recruitment of RBBP5 to the proximal promoters of select proteostasis genes in response to ER stress, concomitant with an increase in promoter-proximal H3K4me3 levels (Fig 5A). Since XBP1s is the major TF driving UPR, we further performed IF on XBP1s and RBBP5 in MEFs and found a strong recruitment of RBBP5 to XBP1s in response to ER stress (Fig 5B and 5C).

**Fig 5:**
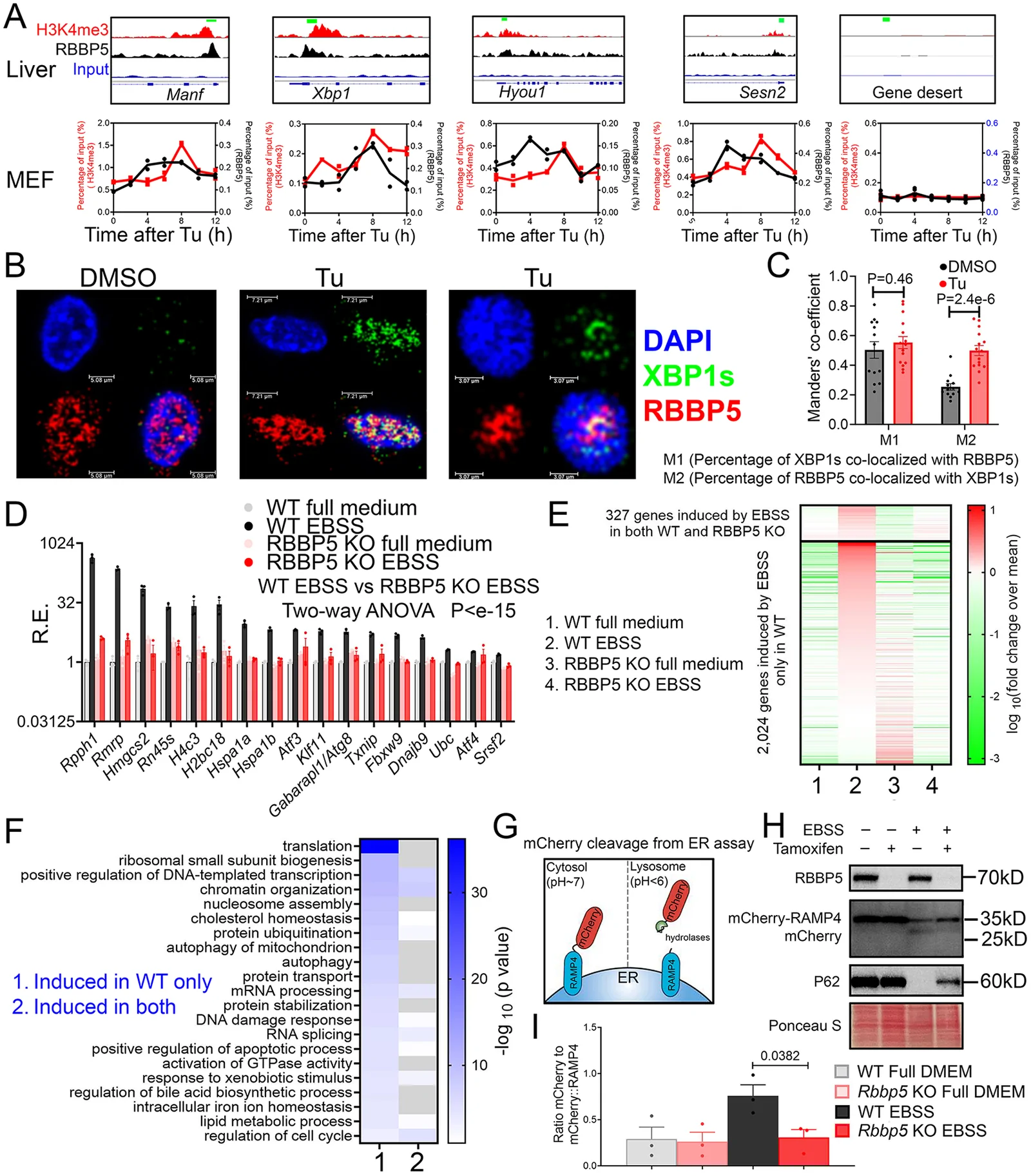
RBBP5 is required for cell-autonomous transcriptional response to ER and nutritional stress. **(A)** Selected genes aligned for RBBP5 and H3K4me3 ChIP-seq signal from CT0 mouse liver and ChIP-qPCR of RBBP5 and H3K4me3 in MEFs treated with 100ng/ml Tu for different hours (*n* = 2 biologically independent duplicates). **(B, C)** Immunofluorescence of XBP1s and RBBP5 in MEFs treated with vehicle control (DMSO) or Tu (100 ng/ml) for 8 h (B) and quantification of Manders’ coefficient of colocalization of XBP1s with RBBP5 (C) (*n* = 13 and 15 cells for DMSO and Tu group, respectively). **(D–F)** Transcriptome profiling of *Rbbp5* (fl/fl) ROSA26-CreERT2 (+/+) MEFs pre-treated with (RBBP5 KO) or without (WT) tamoxifen and then treated with full DMEM media or EBSS for 16 h by RNA-seq. Representative gene expression (D) (*n* = 3 biologically independent samples per condition), heatmap of all genes that are induced by EBSS in WT cells by EBSS (adjP < 0.05) (E), and GO analysis of EBSS-induced genes in only WT or both genotypes (F). **(G)** Diagram of the ER-phagy reporter using the mCherry cleavage from ER assay. **(H, I)** western blot of different proteins (H) and quantification (I) of mCherry cleavage in RAMP4-mCherry-expressing *Rbbp5* (fl/fl) ROSA26-CreERT2 (+/+) MEFs treated with vehicle (WT) or tamoxifen (*Rbbp5* KO) followed by treatment with full DMEM media or EBSS for 16 h (*n* = 3 biologically independent samples per condition). Data: Mean ± S.E.M. Statistical tests used: Two-way ANOVA for D, unpaired two-tailed Student *t* test for C and I. Numerical values are available in S4 Data.

To broadly assess the function of RBBP5 in the transcription regulation of proteotoxic stress response, we utilized orthogonal approaches to both deplete RBBP5 expression and trigger cellular stress. We took advantage of our homozygous *Rbbp5* ^*Flox*^ mice by crossing them with homozygous ROSA26 Cre-ERT2 mice, then isolated and immortalized primary MEFs from homozygous F2 embryos (S12E Fig). Treating these *Rbbp5* (flox/flox); ROSA26 Cre-ERT2 (+/+) MEFs with tamoxifen for 7 days resulted in the complete deletion of both floxed *Rbbp5* alleles (S12F Fig) and a subsequent ~90% reduction in RBBP5 protein and a reduction of H3K4me3 levels (S12G Fig). The remaining RBBP5 protein is likely extremely long-lived and remains stable even after the deletion of underlying coding genes. To trigger ER stress, we used a different stimulus: Dithiothreitol (DTT), which disrupts protein disulfide formation in the ER [67,68]. Similar to Tu, RBBP5 depletion significantly dampened the induction of proteostasis gene expression in response to DTT (S12H Fig).

Next, we tested two additional proteotoxic stressors that are more physiologically relevant: leucine deprivation to simulate amino acid restriction, and a more severe nutrient deprivation stress by culturing cells in Earle’s Balanced Salt Solution (EBSS) for 16 h. Both treatments not only trigger UPR but also activate the broader integrated stress response (ISR), resulting in the induction of autophagy [69]. RBBP5 depletion impaired the transcriptional response to both leucine deprivation and EBSS, leading to a reduced induction of autophagy-promoting genes (S13A Fig). Transcriptome profiling by RNA-seq confirmed the globally defective adaptive stress response to nutrient deprivation (EBSS) in the absence of RBBP5, where only 327 out of 2,351 genes can still be induced in RBBP5 KO MEFs (Fig 5D, 5E and S13B). GO analysis revealed that RBBP5-dependent nutrient deprivation-induced genes are strongly enriched in translation, autophagy, protein ubiquitination, and mRNA processing pathways (Fig 5F).

To investigate the physiological consequence of RBBP5 deletion, we quantified the autophagic flux by utilizing a tandem LC*3* reporter mCherry*-*GFP-LC*3* where an increase in the number of red-fluorescent cytosolic puncta indicates increased autolysosome formation and autophagic flux (S13C Fig) [69]. Consistent with the qPCR results, RBBP5 depleted MEFs failed to induce a robust general autophagy in response to leucine deprivation and further exhibited lower basal level of autophagic flux (S13D and S13E Fig). Western blot analysis of p62/SQSTM1, a marker of autophagic flux due to its degradation during autophagy [70], showed a significant reduction in wild-type, but not RBBP5 depleted cells following leucine deprivation (S13F Fig), thus confirming the defective autophagy phenotype associated with RBBP5 depletion. Since prolonged nutrient deprivation, such as 16 h of EBSS treatment, can robustly induce both general autophagy and autophagy of the ER (ER-phagy) [71], we further measured ER-phagy by utilizing a previously published ER-phagy reporter system where a stably expressed ER-targeting mCherry-RAMP4 fusion is cleaved to free mCherry during starvation-induced ER-phagy [72] (Fig 5G). As shown in Fig 5H and 5I, RBBP5 depletion prevented the cleavage of mCherry in response to EBSS, indicating an impaired ER-phagy. Western blot analysis of p62/SQSTM1 further showed undetectable levels in wild-type cells following EBSS treatment, whereas a significant level remained in RBBP5 depleted cells under the same conditions, thus confirming a reduction of the general autophagy in RBBP5 depleted cells in response to EBSS (Fig 5H). Consistent with weakened stress response and impaired autophagy, RBBP5 depleted MEFs displayed reduced cell survival under nutritional stress (S13G Fig). Taken together, our results demonstrate that RBBP5 is indispensable for regulating the cell-autonomous transcriptional responses to various proteotoxic stresses as a core subunit of the H3K4me3-writing COMPASS complex.

### Proximity labeling reveals H3K4me-associated proteomic architecture of the proteotoxic stress response

H3K4me3 is widely recognized as an active promoter mark, but its precise role in transcriptional regulation remains a subject of debate. Initially, it was believed that H3K4me3 facilitated transcription initiation by recruiting the RNAPII preinitiation complex [66], However, recent evidence suggests that H3K4me3 primarily regulates RNAPII pause-release and contributes to transcriptional elongation, likely through the recruitment of Integrator Complex subunit 11 (INTS11) [47,48]. This discrepancy may stem from the context-specific functions of H3K4me3 in recruiting different transcriptional regulators across different biological processes [73]. To shed light on the potential mechanisms by which H3K4me3 modulates proteostasis stress response, we performed quantitative TurboID-based proximity labeling in MEFs (in biological triplicates per condition), utilizing anti-H3K4me3 antibody (with anti-IgG as negative control) followed by recombinant protein A-TurboID labeling in isolated nuclei of MEFs before (T = 0 h) and 2.5 h and 8 h after ER stress induction by Tu (Figs 6A and S14A) [74]. While the total level of H3K4me3 remains largely unchanged during ER stress (Fig 6B), we identified a total of 239 proteins (*p* < 0.1) whose recruitment to H3K4me3 peaked at 8 h post Tu treatment (S9 Table). Pathway analysis of these 239 proteins revealed expected enrichment of COMPASS subunits (Figs 6C, S14B and S14C), RNAPII subunits (S14D Fig), as well as previously identified Integrator Complex subunits (such as INTS11) (S14E Fig). The previously identified H3K4me3 demethylase KDM5A [48] revealed a much more constant recruitment to H3K4me3 during ER stress, with only a slight increase at 8 h after ER stress induction (S14C Fig).

**Fig 6:**
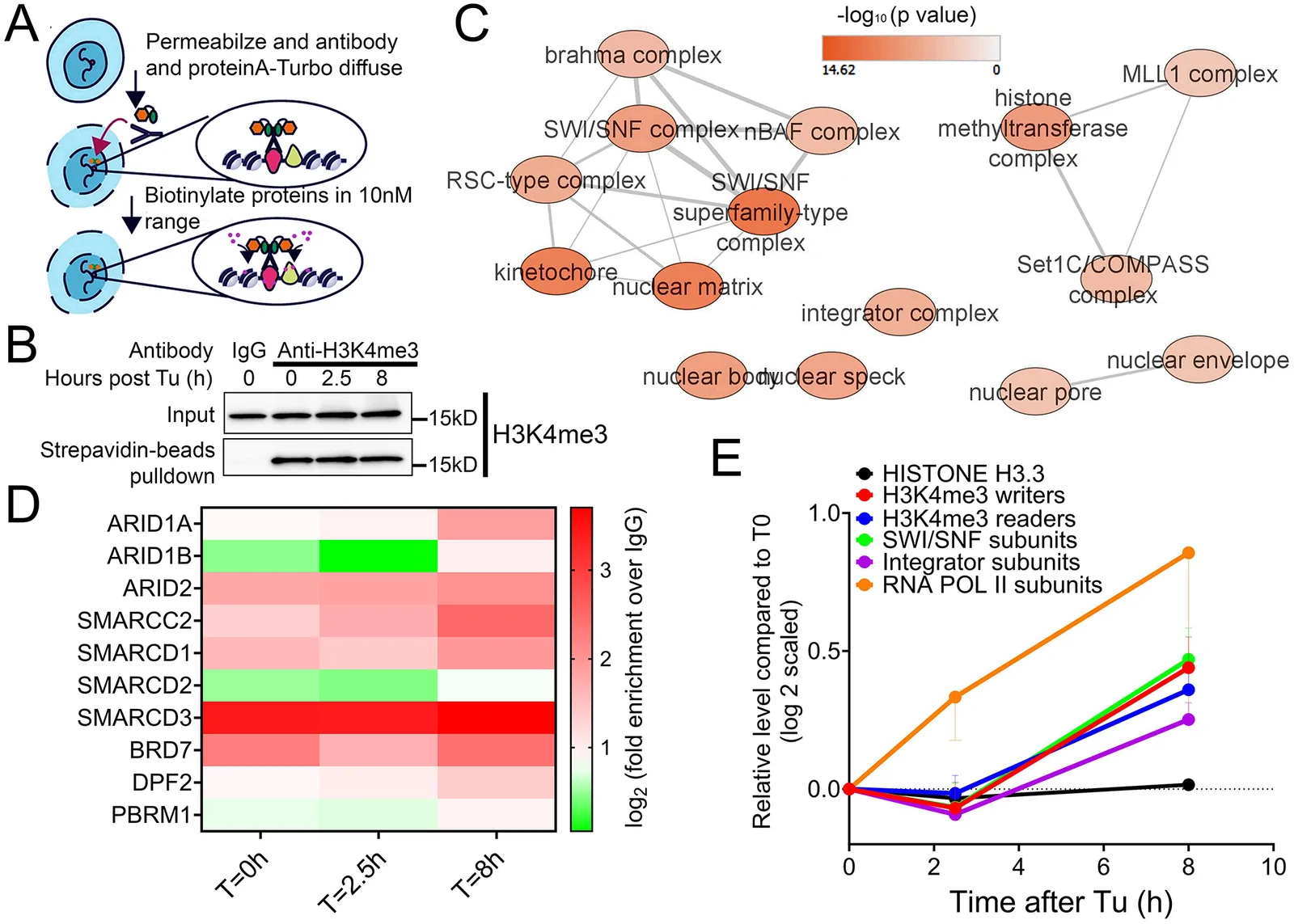
Proximity labeling reveals H3K4me-associated proteomic architecture of the proteotoxic stress response. **(A)** A diagram illustrating the procedure of performing H3K4me3 proximity labeling via using the recombinant Protein A-TurboID. **(B)** western blot analysis showing the nuclear level of total (input) or biotinylated (after streptavidin beads pull-down) level of H3K4me3 in MEF treated with 100ng/ml Tu for 0, 2.5, or 8 h before subject to anti-IgG and anti-H3K4me3 antibody-based proximity labeling. **(C)** Enriched biological network of 239 proteins. **(D)** Heatmap of fold enrichment of different SWI/SNF subunits (over IgG control) at different times after ER stress. **(E)** Quantification of the average relative enrichment of proteins in different complexes at different times after ER stress. Numerical values are available in S4 Data.

Besides previously identified H3K4me3 writer/eraser/readers, we also revealed strong enrichment of SWI/SNF chromatin remodelers on H3K4me3 peaking at 8 h after ER stress induction (Fig 6C and 6D). These include BAF-specific (ARID1A/B), PBAF-specific (ARID2, PBRM1 and BRD7) and shared subunits (SMARCD1/2/3 and SMARCC2) (Fig 6C and 6D) [75]. The timing of SWI/SNF recruitment to H3K4me3 during ER stress closely parallels that of COMPASS and Integrator subunits but lags behind RNAPII subunits (Fig 6E). This temporal pattern suggests that SWI/SNF might potentially play a role in facilitating transcriptional elongation during stress-induced gene activation. Together, these findings demonstrate that H3K4me3 functions as a dynamic signaling hub that coordinates multiple chromatin regulators to ensure effective transcriptional adaptation to proteotoxic stress.

## Discussion

While the epigenetic landscape of the mammalian ~24 h circadian clock was well-studied [42,76–79], the epigenome of the mammalian 12 h ultradian oscillator remains completely unexplored. In this study, we uncovered that unlike the circadian clock, which was modulated by histone acetyltransferase (HAT) [80–82] and histone deacetylase (HDAC) [78,83,84], the epigenome of the 12 h oscillator is associated with ~12 h H3K4me3 oscillation at the proximal promoters of UPR genes. Loss of one of the essential subunits of COMPASS, RBBP5, abolished more than 90% of the hepatic ~12 h transcriptome, while having little effects on the BMAL1-dependent core circadian clock. Knocking down RBBP5 in MEFs further dampened the cell-autonomous 12 h oscillations, but not the circadian rhythms. We further expanded our findings to the epigenetic regulation of response to acute proteotoxic stress and found both increased RBBP5 promoter recruitment and H3K4me3 level are associated with the transcription activation of adaptive stress response genes. Subsequently, depletion of RBBP5 significantly dampened the adaptive transcriptional responses to various proteotoxic stresses.

In our study, we uncovered distinct roles for H3K4me3 and H3K9Ac/H3K27Ac in regulating the 12 h oscillator and transcriptional responses to acute proteotoxic stress. While all three histone modifications—H3K4me3, H3K9Ac, and H3K27Ac—are traditionally linked to transcriptional activation [85–87], our findings suggest that H3K9Ac and H3K27Ac are dispensable for the transcriptional regulation of proteostasis under both basal (the 12 h oscillator) and stress conditions. Interestingly, recent work [88] demonstrates that among epigenetic modifications linked to transcriptional activation, deposition of H3K4me3 at promoters elicits the most robust transcriptional output. These findings provide strong functional support for a causal role of H3K4me3 in gene activation, while also emphasizing that distinct histone marks are not functionally equivalent in their capacity to drive transcription. Building on this, we speculate that H3K4me3 may have been preferentially selected during evolution as a principal epigenetic mechanism for stress-responsive gene regulation. Its ability to rapidly and potently activate transcription could confer a selective advantage by enabling swift adaptation to fluctuating or adverse environmental conditions. However, this hypothesis remains to be rigorously tested, and future studies will be required to determine whether the prominence of H3K4me3 in stress responses reflects evolutionary selection, mechanistic necessity, or both.

The precise role of H3K4me3 in transcriptional regulation remains a topic of debate. Recent evidence suggests that H3K4me3 positively contributes to transcriptional elongation by regulating the pause-release of RNAP II, likely through the recruitment of the Integrator subunit INST11 [73]. Our H3K4me3 proximity labeling not only supports this finding but also unexpectedly reveals increased recruitment of the SWI/SNF complex to H3K4me3 during the UPR in MEFs. Interestingly, the temporal dynamics of SWI/SNF recruitment to H3K4me3 coincide with the recruitment of the Integrator complex but occur after the initial increase in RNAP II subunits, which are likely involved in transcription initiation immediately following stress. This suggests that contrary to the widely accepted view that SWI/SNF primarily facilitates transcription initiation by increasing chromatin accessibility and transcription factor binding to the promoter, it may also play a crucial yet underexplored role in transcription elongation. Although the literature on this aspect is rather limited, some earlier studies hint at this possibility [89,90]. Future research is needed to firmly establish a causal relationship between SWI/SNF function, 12 h oscillator regulation, and the proteostasis stress response, as well as to uncover the underlying molecular mechanisms.

The physiological and pathological roles of mammalian COMPASS subunits, particularly RBBP5, remain incompletely understood [91]. While extensive research has primarily focused on their contributions to organogenesis, early development, and neurodevelopmental disorders, our study significantly broadens the scope of RBBP5’s physiological roles by uncovering its involvement in proteostasis and hepatic metabolism. The human GD-CAT dataset further confirmed that human RBBP5 liver expression is also positively correlated with those involved in proteostasis and mRNA metabolism and negatively associated with those implicated in immune response. This expansion not only advances our understanding of RBBP5’s function but also opens new avenues for exploring its broader impact on cellular and systemic processes. Of particular interest is a recent study that identified heterozygous loss-of-function mutations in *RBBP5* in humans, which were primarily linked to neurodevelopmental disorders [92]. This finding raises intriguing questions about the full spectrum of RBBP5’s physiological roles. Given our discovery of RBBP5’s involvement in metabolic regulation, it is tempting to hypothesize that individuals with RBBP5 loss-of-function mutations may also exhibit metabolic defects. Such a connection would have significant implications, suggesting that RBBP5 plays a multifaceted role in maintaining both neural and metabolic homeostasis. Future research should investigate whether these mutations influence metabolic pathways, potentially contributing to a broader spectrum of clinical symptoms in affected individuals. This line of inquiry could ultimately lead to a deeper understanding of how COMPASS subunits like RBBP5 integrate signals across different tissues to maintain overall physiological balance.

While our findings support a model in which RBBP5 functions as a key epigenetic regulator of proteostasis, an alternative explanation for our observations would be that RBBP5, as a core component of COMPASS, acts as an essential and broadly acting transcriptional cofactor, such that its loss nonspecifically perturbs global transcription, including genes involved in proteostasis. However, several lines of evidence argue against this interpretation. Most notably, RBBP5 itself exhibits robust ~12 h rhythms across multiple tissues, indicating that its activity is temporally regulated rather than constitutive. In this context, we favor a temporal function–relationship model, whereby genes and pathways involved in related biological processes cluster in time and share similar temporal profiles [93]. The tight synchronization between the ~12 h rhythm of RBBP5 and that of UPR/proteostasis gene expression strongly supports a more specific, functionally integrated role for RBBP5 in orchestrating proteostasis dynamics, rather than a purely global and epistatic effect on transcription. Consistent with this principle, our previous work demonstrated that nuclear speckle phase separation dynamics also follow a ~12 h rhythm in phase with UPR/proteostasis programs [25], and that nuclear speckles play key roles in regulating transcriptional responses to proteotoxic stress, including the heat shock and ER stress [94–97]. Together, these findings support a model in which temporally coordinated regulation underlies proteostasis control. Finally, we propose that the 12 h oscillator serves as an effective “discovery tool” for uncovering previously unrecognized mechanisms of proteostasis regulation. Continued exploration of the 12 h oscillator will likely reveal additional hidden principles governing proteostasis beyond nuclear speckles and RBBP5.

## Materials and methods

### Ethics statement

The animal studies were carried out in accordance with the National Institutes of Health guidelines and were granted formal approval by the University of Pittsburgh’s Institutional Animal Care and Use Committee (approval number IS00013119 and IS00013119).

### Plasmids

pCDH-EF1a-mCherry-EGFP-LC3B was a gift from Sang-Hun Lee (Addgene plasmid # 170446; http://n2t.net/addgene:170446; RRID: Addgene_170446) [98]. pLenti-X1-hygro-mCherry-RAMP4 was a gift from Jacob Corn (Addgene plasmid # 118391; http://n2t.net/addgene:118391; RRID:Addgene_118391) [72].

### The generation of *Rbbp5* ^(flox/flox)^ mice

The Rbbp5-flox allele was generated by the Mouse Embryo Services Core (University of Pittsburgh, Department of Immunology). The targeting strategy is based on the Easi-CRISPR method [99]. In brief, fertilized embryos from C57BL/6J background, produced by natural mating, were microinjected in the pronuclei with a mixture of 0.67 µM EnGen Cas9 protein (New England Biolabs, Cat.No. M0646T), two Cas9 guides RNA: Rbbp5-695rev and Rbbp5-951forw (21.23 ng/µl each) and a long single stranded oligonucleotides Rbbp5-flox-ssODN (5 ng/µl). The long single stranded oligonucleotide encoding the donor sequence was synthesized by Integrated DNA Technologies (IDT).

To produce the sgRNA, a double strand linear DNA template is created by annealing of the following target specific oligonucleotides with a common primer (gRNA-Scaffold-R: 5′-AAAAAAGCACCGACTCGGTGCCACTTTTTCAAGTTGATAACGGACTAGCCTTATTTTAACTTGCTATTTCTAGCTCTAAAAC-3′) containing the full tracrRNA sequence and then PCR-amplified as previously described [100]. The sgRNA was synthesized using HiScribe T7 Quick High Yield RNA Synthesis Kit (New England Biolabs, Cat. No. E2050S).

T7-Rbbp5-695rev sequence:

TAATACGACTCACTATAGGAAGCATTCTAAGATTAAACCGTTTTAGAGCTAGAAATAGCA

T7-Rbbp5-951forw sequence:

TAATACGACTCACTATAGGAGTAACAGATAGTATTCCGAGTTTTAGAGCTAGAAATAGCA

Rbbp5-flox-ssODN sequence:

gcagttaataaacttgaagtgtttatatagaaaatacatcaggatggcaaccaaaccctttagatacctgagattttattctcttatcccaggtATAACTTCGTATAGCATACATTATACGAAGTTATgaattcttaatcttagaatgcttagtctgtttcatgctgaagataagttgtcagaactcacattgttactgatttttcgttcagtgactcgtctgttttgtcataggaaaattggagtgcatttgcaccagacttcaaagagttggatgaaaatgtagaatatgaggaaagagaatcagaatttgatattgaggatgaagataagagtgagcctgagcaaacaggtgatgcctctcagtaacagatagtattgaattcATAACTTCGTATAGCATACATTATACGAAGTTATccgaagggaaggactgtcttactcaatgctctttgaattagtgaatgcatttatttactttgtgttgtaactgtgctcaaagcttcatctgccacgtttccctcacttgtacatagttttcacagtgcttgaagagtcttactg

The injected zygotes were cultured overnight, the next day the embryos that developed to the 2-cell stage were transferred to the oviducts of pseudo pregnant CD1 female surrogates. Following optimization of the cycling conditions, the potential founder mice were genotype with the following primers pair, using Q5 High-Fidelity DNA Polymerase (NEW ENGLAND BIOLABS, Cat. no. M0491L). The body weight of male adult mice was measured weekly.

*Rbbp5* genotyping forward primer: AGTTCAGAGCTGGTTTCCAAC

*Rbbp5* genotyping reverse primer: AGGTAATGCCCATGTGAGCC

### Biological rhythm study in mice

All mice used for biological rhythm study are in C57BL/6J background, male and between 3 and 4 months of age. Wild-type C57BL/6J mice (*n* = 12), *Rbbp5* (flox/flox); Alb-CRE (−/−) (*n* = 48) and *Rbbp5* (flox/flox); Alb-CRE (+/−) mice (*n* = 48) [*Rbbp5* (flox/flox); Alb-CRE (−/−) and *Rbbp5* (flox/flox); Alb-CRE (+/−) mice were littermates] were first entrained under LD12:12 conditions for 2 weeks before transferred to constant darkness for 24 h. Mice were then sacrificed via cervical dislocation at a 2 h interval [for *Rbbp5* (flox/flox); Alb-CRE (−/−) and *Rbbp5* (flox/flox); Alb-CRE (+/−) mice] or 4 h interval (for wild-type C57BL/6J mice) for a total of 48 h under constant darkness. Mice were fed *ad libitum* during the entire experiment.

### Food intake and locomotor activity monitoring

Promethion Multi-plexed Metabolic Cage System was used for real-time measuring of food intake and locomotor activity. Male *Rbbp5* ^*Flox*^ and *Rbbp5* ^*LKO*^ mice between 3 and 4 months of age were acclimated to the chambers and ad libitum food intake was monitored for 48 h under LD12:12 followed by 48 h of constant darkness. *n* = 6 for *Rbbp5* ^*Flox*^ and *n* = 8 for *Rbbp5* ^*LKO*^ mice for LD12:12 study. *n* = 5 for *Rbbp5* ^*Flox*^ and *n* = 3 for *Rbbp5* ^*LKO*^ mice for DD study.

### ER stress induction study in mice

For acute ER stress induction experiment, male littermates *Rbbp5* (flox/flox); Alb-CRE (−/−) and *Rbbp5* (flox/flox); Alb-CRE (+/−) mice between 5 and 7 months of age were randomly divided into two groups, and intraperitoneally injected with 0.05 mg/kg body weight of tunicamycin dissolved in 500 µl 3% DMSO in PBS or vehicle control (3% DMSO in PBS), respectively. Mice were injected with Tu between 9 and ~10 AM in the morning and 8 h later dissected for transcriptomic and western blot analysis for different tissues. The sample size is *n* = 3 for *Rbbp5* ^*Flox*^ DMSO, *n* = 3 for *Rbbp5* ^*Flox*^ Tu, *n* = 4 for *Rbbp5* ^*LKO*^ DMSO, n = 4 for *Rbbp5* ^*LKO*^ Tu.

For experiments involving RBBP5 liver-overexpressing mice, AAV8-TBG-EGFP and AAV8-TGB-mRBBP5 viruses were customarily designed and produced from Vector Biolab (Malvern, PA). Male wildtype C57BL/6J mice between 2 and 3 months of age were tail-vein injected with 0.5 x 10^11 genome copies of AAV8-TBG-EGFP or AAV8-TGB-mRBBP5 virus diluted in 100 µl of PBS. Thirty days later, mice injected with either viruses were randomly divided into two cohorts and intraperitoneally injected with 0.05 mg/kg body weight of tunicamycin dissolved in 500 µl 3% DMSO in PBS or vehicle control (3% DMSO in PBS). Mice were injected with Tu between 9 and ~10 AM in the morning and 8 h later dissected for transcriptomic and western blot analysis for different tissues. The sample size is *n* = 3 for AAV8-TBG-EGFP DMSO, *n* = 4 for AAV8-TBG-EGFP Tu, *n* = 4 for AAV8-TGB-mRBBP5 DMSO, and *n* = 4 for AAV8-TGB-mRBBP5 Tu.

### Generation of stable autophagy reporter and ER-phagy reporter cell line

Primary MEFs were isolated from *Rbbp5* (flox/flox); Rosa26 CRE (+/+) mice and immortalized with SV40 lentivirus as previously described [101]. For mCherry-EGFP-LC3B-expressing autophagy reporter MEFs, lentiviruses packaged in HEK293T cells with co-transfection of pCDH-EF1a-mCherry-EGFP-LC3B, pMD2.G and psPAX2 plasmids were used to infect *Rbbp5* (flox/flox); Rosa26 CRE (+/+) MEFs with a multiplicity of infection (MOI) of 3 for three times to achieve near complete infection. For mCherry-RAMP4-expressing ER-phagy reporter MEFs, lentiviruses packaged in HEK293T cells with co-transfection of pLenti-X1-hygro-mCherry-RAMP4, pMD2.G and psPAX2 plasmids were used to infect *Rbbp5* (flox/flox); Rosa26 CRE (+/+) MEFs with a multiplicity of infection (MOI) of 3. Stable MEFs were selected in the presence of 200 μg/ml hygromycin.

### siRNA Transient Transfections

Immortalized MEFs isolated from wild-type C57BL/6J mice were transfected with 10µM of different siRNAs for 24~48 h with Lipofectamine RNAiMAX reagents (Life technologies) per the manufacturer’s instructions. Source of siRNA are as follows: siGENOME nontargeting siRNA pool (Dharmacon, D-001206-1305), siGENOME SMARTpool mouse *Rbbp5* siRNA (Dharmacon, L-054560-01-0005), siGENOME SMARTpool mouse *Ash2l* siRNA (Dharmacon, L-048754-01-0005), siGENOME SMARTpool mouse *Setd1a* siRNA (Dharmacon, L-051358-01-0005), siGENOME SMARTpool mouse *Kat2a/Gcn5* siRNA (Dharmacon, L-040665-01-0005), siGENOME SMARTpool mouse *Kat2b/Pcaf* siRNA (Dharmacon, L-049885-01-0005) and siGENOME SMARTpool mouse *Xbp1* siRNA (Dharmacon, L-040825-00-0005). MEFs were transfected with 10 µM of different siRNAs for 48 h and treated with DMSO or 100 ng/ml of tunicamycin for 8 h.

### Real-time luminescence assay

Stable *Manf*-dluc MEFs were transfected with nontargeting, *Rbbp5,* or *Ash2l* siRNA for 48 h in DMEM (4.5g/L glucose) supplemented with 10% FBS and treated with 25 ng/ml tunicamycin in DMEM for 2 h before subjected to real-time luminescence assay using a Lumicycle (Actimetrics). Briefly, after tunicamycin treatment, MEFs were washed with 1x PBS and cultured with DMEM (4.5 g/L glucose) supplemented with 0.1 mM luciferin and 10 mM HEPES buffer in 35 mm tissue culture dishes in the absence of serum and transferred immediately to Lumicycle for real-time luminescence analysis. Periods of oscillation were identified by embedded Periodogram function.

### Immunofluorescence (IF)

Immunofluorescence was performed as previously described [25]. Briefly, liver OCT sections were fixed in cold acetone for 10 mins at −20 ºC. The sections were then air dried, rehydrated with PBS and permeabilized with PBS+ 0.1% Triton X-100. The sections were then blocked with 10% goat serum at room temperature for 1 h. Primary antibody against RBBP5 (Bethyl, A300-109A) was added to the OCT section at 1:1000 dilution overnight at 4 ºC. Next day, sections were washed three times with PBS and stained with Alexa-555 anti-rabbit secondary antibody at room temperature for 2 h. After that, the sections were washed with PBS three times and counterstained with DAPI before mounting (with ProlongGold Glass) and imaging using Leica SP8 lightening confocal microscope (Leica Microsystems).

### Time-lapse microscopy for autophagy reporter MEFs

Time-lapse imaging was performed using SP8 lightening confocal microscope (Leica Microsystems) with Okolab stage top incubator to maintain constant CO_2_ (5%), temperature (37 ºC) and humidity (90%). mCherry-EGFP-LC3B-expressing *Rbbp5* (flox/flox); Rosa26 CRE (+/+) MEFs were plated into 8-well chamber slides in full DMEM media and treated with (to deplete RBBP5) or without tamoxifen (3 µM/ml) for seven days before replacing with full DMEM or leucine-free media counterstained with DAPI and images were taken every 2 h for a total of 16 h. The number of cytosolic autophagosomes (GFP and mCherry double positive foci) and autolysosomes (GFP negative and mCherry positive foci) per cell were quantified manually.

### Immunoblot

Nuclear extracts were made from liver according to a previously published protocol [102]. Whole cell lysates were lysed in RIPA buffer as previously described [25]. Protein concentrations were determined by Bradford assays (Bio-Rad), and aliquots were snap-frozen in liquid nitrogen and stored at −80 °C until usage. Immunoblot analyses were performed as described previously [80]. Briefly, 25 ~50 µg proteins separated by 4~20% gradient SDS-PAGE gels (Biorad) were transferred to nitrocellulose membranes, blocked in TBST buffer supplemented with 5% bovine serum albumin (BSA) or 5% fat-free milk and incubated overnight with primary antibody anti-PERK (Cell signaling, #3192), anti-phospho-PERK (Thr908) (Thermo Fisher, MA5–15033), anti-ATF4 (Cell signaling, #11815), anti-IRE1α (Cell signaling, #3294), anti-phospho-IRE1α (Ser724) (ABclonal, AP0878), anti-XBP1s (Biolegend, 658802), anti-RBBP5 (Bethyl, A300-109A), anti-RBBP5 (D3I6P) (Cell signaling, #13171), anti-WDR5 (Cell signaling, #13,105), anti-ASH2L (Bethyl, A300-489A), anti-ASH2L (Bethyl, A300-112A), anti-SETD1A (Diagenode, CS-117-100), anti-SETD1B (Diagenode, CS-118-100), anti-MLL1-c (D6G8N) (Cell signaling, #14197), anti-mCherry (E5D8F) (Cell signaling, #43590), anti-p62/SQSTM1 (Santa Cruz, sc-28359) and anti-β-ACTIN (Cell signaling, #12620) at 4 °C. Blots were incubated with an appropriate secondary antibody coupled to horseradish peroxidase at room temperature for 1 h, and reacted with ECL reagents per the manufacturer’s (Thermo) suggestion and detected by Biorad ChemiDoc MP Imaging System.

### Co-immunoprecipitation (Co-IP)

Nuclear extracts (NE) were made from liver according to our published protocol [102]. Protein concentrations were determined by Bradford assays (Bio-Rad), and aliquots were snap-frozen in liquid nitrogen and stored at −80 °C until usage. 200 μg of NE was used for per IP. 5 μg of different antibodies or control IgG were coupled to 1.5 mg of magnetic Dynabeads (Life Technologies) for every IP using Dynabeads Antibody Coupling Kit (Life Technologies) per manufactures’ protocol. Co-IP was essentially carried out as previously described [80], except that coupled antibody Dynabeads were added to the NEs for incubation. The antibodies used for Co-IP are as follows: anti-XBP1s (Biolegend, 658802).

### qRT-PCR

Total mRNA was isolated from murine embryonic fibroblasts (MEFs) or mouse liver with PureLink RNA mini kit (Life Technologies) with additional on-column DNase digestion step to remove genomic DNA per the manufacturer’s instructions. Reverse transcription was carried out using 5 µg of RNA using Superscript III (Life Technologies) per the manufacturer’s instructions. For gene expression analyses, cDNA samples were diluted 1/30-fold (for all other genes except for 18sRNA) and 1/900-fold (for 18sRNA). qPCR was performed using the SYBR green system with sequence-specific primers. All data were analyzed with 18S or *β-actin* as the endogenous control. qPCR primer sequences are as follows and all primers span introns, except for primers for quantifying pre-mRNAs:

Mouse *Rbbp5* forward primer: CAGAGCCTATGCAGAAGCTGCA

Mouse *Rbbp5* reverse primer: CTTCACCAGGTTGCCAATGCTC

Mouse *Ddit3* forward primer: GGAGGTCCTGTCCTCAGATGAA

Mouse *Ddit3* reverse primer: GCTCCTCTGTCAGCCAAGCTAG

Mouse *Creld2* forward primer: CTACACCAAGGAGAGTGGACAG

Mouse *Creld2* reverse primer: TCTGTCTCCTCAAAGCCTTCCG

Mouse *Hspa5* forward primer: TGTCTTCTCAGCATCAAGCAAGG

Mouse *Hspa5* reverse primer: CCAACACTTCCTGGACAGGCTT

Mouse *Dnajb11* forward primer: TGTGACCGTCTCACTGGTTGAG

Mouse *Dnajb11* reverse primer: CCCTTTCTTCCACAGCTTGGCT

Mouse *Pdia4* forward primer: TGGGCTCTTTCAGGGAGATGGT

Mouse *Pdia4* reverse primer: GGGAGACTTTCAGGAACTTGGC

Mouse *Hsp90b1* forward primer: GTTTCCCGTGAGACTCTTCAGC

Mouse *Hsp90b1* reverse primer: ATTCGTGCCGAACTCCTTCCAG

Mouse *Syvn1* forward primer: CCAACATCTCCTGGCTCTTCCA

Mouse *Syvn1* reverse primer: CAGGATGCTGTGATAAGCGTGG

Mouse *Gdf15* forward primer: ACTCAGTCCAGAGGTGAGATTG

Mouse *Gdf15* reverse primer: GGGGCCTAGTGATGTCCCAG

Mouse *Il1a* forward primer: ACGGCTGAGTTTCAGTGAGACC

Mouse *Il1a* reverse primer: CACTCTGGTAGGTGTAAGGTGC

Mouse *Il1b* forward primer: TGGACCTTCCAGGATGAGGACA

Mouse *Il1b* reverse primer: GTTCATCTCGGAGCCTGTAGTG

Mouse *Fga* forward primer: GGATTCTAACTCACTGACCAGGA

Mouse *Fga* reverse primer: CCTCAGGATCTCAATTCTGCGC

Mouse *C3* forward primer: CGCAACGAACAGGTGGAGATCA

Mouse *C3* reverse primer: CTGGAAGTAGCGATTCTTGGCG

Mouse *Tifa* forward primer: AGCAAGAAAACCAGTTTGATGGTAG

Mouse *Tifa* reverse primer: GCAACAGGAACTGATACTCTCCG

Mouse *Fgl1* forward primer: GGAAACTGTGCTGAGGAAGAGC

Mouse *Fgl1* reverse primer: TCCGTTTCTGCCCTGTAGGAAC

Mouse *Fgb* forward primer: TGACACCTCCATCAAGCCGTAC

Mouse *Fgb* reverse primer: GGTCCCATTTCCTGCCAAAGTC

Mouse *Il6* forward primer: CTTCCATCCAGTTGCCTTCT

Mouse *Il6* reverse primer: CTCCGACTTGTGAAGTGGTATAG

Mouse *Herpud1* forward primer: acctgagccgagtctaccc

Mouse *Herpud1* reverse primer: aacagcagcttcccagaataaa

Mouse *Nbr1* forward primer: GGATTTAAAGCACCTCCTGATTCC

Mouse *Nbr1* reverse primer: ATTGGGTCCCACTCAGGTCT

Mouse *Sesn2* forward primer: CTGGCCGAACTCATCCAAG

Mouse *Sesn2* reverse primer: CTGCCTCATGCGTTCCATC

Mouse *Sqstm1* forward primer: AACAGATGGAGTCGGGAAAC

Mouse *Sqstm1* reverse primer: AGACTGGAGTTCACCTGTAGA

Mouse *Stbd1* forward primer: CTGGGAAGTAGATGGGAAAGTG

Mouse *Stbd1* reverse primer: TCGGTGTTTGTGGTGTAGTG

Mouse *Sirt1* forward primer: TGACAGAACGTCACACGCCA

Mouse *Sirt1* reverse primer: ATTGTTCGAGGATCGGTGCCA

Mouse *Gcn5* forward primer: TTGATTGAGCGCAAACAGGC

Mouse *Gcn5* reverse primer: CAGCCTGTCTCTCGAATGCC

Mouse *Pcaf* forward primer: CGTGAAGAAGGCGCAGTTG

Mouse *Pcaf* reverse primer: CAGGACTCCTCTGCCTTGC

Mouse *Ash2l* forward primer: gctgtgtctgctagtgggaac

Mouse *Ash2l* reverse primer: catcttgctgcttacgcttg

Mouse *Setd1a* forward primer: gggtagcaccccctactctc

Mouse *Setd1a* reverse primer: gggtttgaaggaggttgaagt

Mouse *Eif2ak3* forward primer: ccttggtttcatctagcctca

Mouse *Eif3ak3* reverse primer: atccagggaggggatgat

Mouse total *Xbp1* forward primer: gggtctgctgagtcc

Mouse total *Xbp1* reverse primer: cagactcagaatctgaagagg

Mouse *Sec23b* forward primer: tgaccaaactggacttctgga

Mouse *Sec23b* reverse primer: aaagaatctcccatcaccatgt

Mouse *Manf* forward primer: gacagccagatctgtgaactaaaa

Mouse *Manf* reverse primer: tttcacccggagcttcttc

Mouse *Hyou1* forward primer: GAGGCGAAACCCATTTTAGA

Mouse *Hyou1* reverse primer: GCTCTTCCTGTTCAGGTCCA

Mouse *Atf4* forward primer: CCACTCCAGAGCATTCCTTTAG

Mouse *Atf4* reverse primer: CTCCTTTACACATGGAGGGATTAG

Mouse *Atf6* forward primer: CATGAAGTGGAAAGGACCAAATC

Mouse *Atf6* reverse primer: CAGCCATCAGCTGAGAATTAGA

Mouse 18s RNA forward primer: ctcaacacgggaaacctcac

Mouse 18s RNA reverse primer: cgctccaccaactaagaacg

Mouse *β-actin* forward primer: aaggccaaccgtgaaaagat

Mouse *β-actin* reverse primer: gtggtacgaccagaggcatac

### RNA-Seq to identify RBBP5-dependent 12 h hepatic transcriptome

Mouse liver tissues were collected from *Rbbp5* ^*Flox*^ (*n* = 2) and *Rbbp5* ^*LKO*^ (*n* = 2) mice at 2 h intervals for a total of 48 h under constant darkness conditions. Total RNA was isolated from mouse liver with miRNeasy Mini Kit (Qiagen) per the manufacturer’s instructions. RNA was assessed for quality using an Agilent TapeStation 4150/Fragment Analyzer 5300 and RNA concentration was quantified on a Qubit FLEX fluorometer. Libraries were generated with the Illumina Stranded mRNA Library Prep kit (Illumina: 20040534) according to the manufacturer’s instructions. Briefly, 200 ng of input RNA was used for each sample. Following adapter ligation, 12 cycles of indexing PCR were completed, using IDT for Illumina RNA UD Indexes (Illumina: 20040553-6). Library quantification and assessment were done using a Qubit FLEX fluorometer and an Agilent TapeStation 4150/Fragment Analyzer 5300. The prepared libraries were pooled and sequenced using NoveSeq 6000 (Illumina), generating an average of 40 million 2 × 100 bp paired end reads per sample. RNA-Seq library preparation and sequencing were performed at UPMC Genome Center. Raw RNA-seq FASTQ files were analyzed by FastQC for quality control. Adaptors and low-quality reads were filtered by Trimmomatic [103]. Then the processed reads were aligned by HISAT2 [104] against mouse reference mm10. Gene and isoform FKPM values were calculated by Cufflinks. Since the background gene expression level is FPKM = 0.1 in mouse liver [17], only those genes with average gene expression across 48 hours in Rbbp5 ^*Flox*^ mice larger than 0.1 were included for rhythm-identification analysis. Averaged FPKM values at each time were used for cycling transcripts identification by the eigenvalue/pencil method. Raw temporal data were subject to polynomial detrend (*n* = 2) first, and superimposed oscillations were identified using the previously described eigenvalue/pencil method on the detrended dataset [17]. Specifically, three oscillations were identified from each gene. Criterion for circadian gene is period between 21 h and 27 h, decay rate between 0.9 and 1.1; for ~12 h genes: period between 10 h and 13 h, decay rate between 0.9 and 1.1. The relative amplitude is calculated via dividing the amplitude by the mean expression value of each gene. All analysis were performed in MatlabR2017A. Heat maps were generated by Gene Cluster 3.0 and TreeView 3.0 alpha 3.0 using log2 mean-normalized values. RAIN analysis was performed as previously described in Bioconductor (3.4) (http://www.bioconductor.org/packages/release/bioc/html/rain.html) [55] using the full detrended dataset with duplicated at each time point.

For the eigenvalue method, every gene consists of multiple superimposed oscillations. Therefore, we define a circadian gene as any gene that exhibits a superimposed circadian rhythm, regardless of its relative amplitude compared to other superimposed oscillations. Similar definitions apply to 12 h genes. Under this definition, a gene can be both a circadian and 12 h-cycling gene. By comparison, we define a dominant circadian gene as any gene whose superimposed circadian rhythm has the largest amplitude among all oscillations. Similar definitions also apply to 12 h genes. Under this definition, dominant circadian and dominant 12 h genes are mutually exclusive.

### RNA-seq for identifying RBBP5-dependent acute transcriptional response to ER stress

Mouse liver tissues were collected from *Rbbp5* ^*Flox*^ and *Rbbp5* ^*LKO*^ mice intraperitoneally treated with or without Tu for 8 h. Total mRNA was isolated from mouse liver with PureLink RNA mini kit (Life Technologies) with additional on-column DNase digestion step to remove genomic DNA per the manufacturer’s instructions. Detailed procedures for transcriptome sequencing were described previously [105] and were supported by the Genomics and Systems Biology Core of the Pittsburgh Liver Research Center. Briefly, the mRNA samples per mouse were processed into short-read libraries using the Bio-Rad SEQuoia Dual Indexed Primers. Next, the circularization was performed using the Element Biosciences Adept Library Compatibility Kit v1.1 based on the manufacturer’s instructions, followed by the quantification using qPCR with the provided standard. Finally, the libraries were measured by the Element Biosciences AVITI system to sequence paired end reads with 75 bp each. The raw FASTQ files from RNA-seq were trimmed by Trimmomatic [103] to filter out low-quality reads. The surviving reads were then aligned to the mouse mm10 reference genome by STAR aligner [106]. Fragments Per Kilobase of transcript per Million mapped reads (FPKM) values per library were quantified by the tool Cufflinks [107]. Log 2 normalized fold induction and adjusted *p* values between *Rbbp5* ^*Flox*^ DMSO and *Rbbp5* ^*LKO*^ DMSO, *Rbbp5* ^*Flox*^ Tu and *Rbbp5* ^*LKO*^ Tu, *Rbbp5* ^*Flox*^ DMSO and *Rbbp5* ^*Flox*^ Tu, and *Rbbp5* ^*LKO*^ DMSO and *Rbbp5* ^*LKO*^ Tu were calculated by using the iDEP (integrated Differential Expression and Pathway analysis) online application [108].

### Chromatin Immunoprecipitation (ChIP)-Seq and ChIP-qPCR

ChIP for RBBP5 was performed using anti-RBBP5 antibody (Bethyl, A300-109A) as previously described [17]. Briefly, mouse liver samples were submerged in PBS + 1% formaldehyde, cut into small (~1 mm^3^) pieces with a razor blade and incubated at room temperature for 15 min. Fixation was stopped by the addition of 0.125 M glycine (final concentration). The tissue pieces were then treated with a TissueTearer and finally spun down and washed twice in PBS. Chromatin was isolated by the addition of lysis buffer, followed by disruption with a Dounce homogenizer. The chromatin was enzymatically digested with MNase. Genomic DNA (Input) was prepared by treating aliquots of chromatin with RNase, Proteinase K and heated for reverse-crosslinking, followed by ethanol precipitation. Pellets were resuspended and the resulting DNA was quantified on a NanoDrop spectrophotometer. An aliquot of chromatin (30 μg) was precleared with protein A agarose beads (Invitrogen). Genomic DNA regions of interest were isolated using 4 μg of antibody. Complexes were washed, eluted from the beads with SDS buffer, and subjected to RNase and proteinase K treatment. Crosslinking was reversed by incubation overnight at 65 °C, and ChIP DNA was purified by phenol-chloroform extraction and ethanol precipitation. The DNA libraries were prepared at the University of Pittsburgh and sequenced at Gene by Gene, Ltd and Novagene per standard protocols. DNA libraries were prepared with Ovation Ultralow V2 DNA-Seq library preparation kit (NuGen) using 1ng input DNA. The size selection for libraries was performed using SPRIselect beads (Beckman Coulter) and purity of the libraries was analyzed using the High Sensitivity DNA chip on Bioanalyzer 2100 (Agilent). The prepared libraries pooled and sequenced using Nova-Seq 6000 (Illumina), generating ~30 million 75 bp single-end reads per sample for the first repeat and sequenced by Nova-Seq (Illumina), generated ~50 million 150 bp pair-end reads for the second repeat. ChIP-qPCR for MEFs was essentially performed the same way as previously described with anti-RBBP5 (Bethyl, A300-109A) and anti-H3K4me3 (Active Motif, 61379) antibodies, except that the MEFs were directly fixed with 1% formaldehyde before subject to nuclei isolation and chromatin immunoprecipitation. The primers used for ChIP-qPCR are as follows:

Gene desert forward primer: GCAACAACAACAGCAACAATAAC

Gene desert reverse primer: CATGGCACCTAGAGTTGGATAA

*Manf* promoter forward primer: CCCTTAAATGGGTCAACGTCTC

*Manf* promoter reverse primer: GGCGCTAAACCCAAGGAAA

*Xbp1* promoter forward primer: TCCGTACGGTGGGTTAGAT

*Xbp1* promoter reverse primer: ACCTTCTTCTGTGCCTGTG

*Hyou1* promoter forward primer: CCGTGTGGGTACGTCCT

*Hyou1* promoter reverse primer: GACGGCTGCTCCATCCT

*Sesn2* promoter forward primer: ACAGGAGGCCGGGACTA

*Sesn2* promoter reverse primer: CTGGGCTGAAAGGAGTGTCTAT

Negative control region 1 forward primer: TTCTGTCCCTGCAGTTCTTTC

Negative control region 1 reverse primer: GGCTGTGGTGTCTTGAGATTAG

Negative control region 2 forward primer: ACGTGATCCTAAGACCTCTAGT

Negative control region 2 reverse primer: GGGTAGCACTCTCCTTAGTTTC

### ChIP-Seq analysis

The sequences identified were mapped to the mouse genome (UCSC mm10) using BOWTIE function in Galaxy Deeptool (https://usegalaxy.org/) [109]. Only the sequences uniquely mapped with no more than 2 mismatches were kept and used as valid reads. PCR duplicates were also removed. Peak calling was carried out by MACS2 (version 2.1.1.20160309) in Galaxy (options --mfold 5, 50 --*p* value 1e−4), on each ChIP-seq file against input. To account for the different sequencing depths between samples, the BAM files generated from MACS2 were RPKM normalized to sequencing depth using the bamCoverage function in Galaxy Deeptool and the bigwig files were generated accordingly. The relative intensity of each RBBP5 binding site is further calculated via the computeMatrix function with the RPKM normalized bigwig files and bed files from the peak calling as inputs by calculating the area under the curve. Temporal RBBP5 binding intensities from the two independent runs were processed separately, with each dataset detrended using a second-order polynomial followed by Z-score normalization. The normalized signals were then used for 12 h rhythm detection using RAIN.

### Proximity labeling

Proximity labeling was performed using Protein A-TurboID as previously described [74]. Briefly, target cells were grown to 90%–100% confluency in 15 cm dishes, harvested in PBS using a cell scraper, and pelleted by centrifugation. Nuclei were isolated by resuspending the cell pellet in ice-cold HLB buffer, incubating on ice, and washing through sequential centrifugation steps. Isolated nuclei were incubated with digitonin buffer containing ~2 µg of IgG or H3K4me3 antibody (Active Motif, 61379) for 20 min at room temperature with shaking, followed by incubation with 1.4 µg of Protein A-TurboID for 30 min at 4 °C. After washing, biotinylation was induced by incubating samples in biotinylation buffer (containing ATP, MgCl_2_, and 500 µM biotin) for 10 min at 37 °C. Nuclei were lysed in RIPA buffer at 4 °C overnight, sonicated, and centrifuged to collect the supernatant. Streptavidin affinity purification was performed by incubating lysates with M-270 streptavidin beads for 2 h at room temperature or overnight at 4 °C. Beads were washed extensively with RIPA buffer, and bound proteins were eluted by boiling at 100 °C for 15 min in 70 µL of 1.5X LDS buffer. Samples were clarified by centrifugation and magnetic separation.

For protein identification, half of each sample was separated ~1.5 cm on a 10% Bis-Tris Novex mini-gel (Invitrogen) using the MES buffer system. The gel was stained with Coomassie, and each lane was excised into 10 equally sized segments. Gel pieces were processed using a DigestPro robot (CEM) with the following protocol: washes with 25 mM ammonium bicarbonate and acetonitrile, reduction with 10 mM dithiothreitol at 60 °C, alkylation with 50 mM iodoacetamide at room temperature, digestion with trypsin (Promega) at 37 °C for 4 h, and quenching with formic acid. The resulting peptides were analyzed by nano LC-MS/MS using a Waters M-class HPLC system interfaced with a ThermoFisher Fusion Lumos mass spectrometer. Peptides were loaded onto a trapping column and eluted over a 75 µm analytical column at 350 nL/min with a 30-min gradient. MS and MS/MS spectra were acquired in the Orbitrap at resolutions of 60,000 and 15,000 FWHM, respectively. Data were searched using Mascot against the SwissProt Mouse database with a 10 ppm peptide mass tolerance and a 20 ppm fragment mass tolerance. Search parameters included trypsin digestion, fixed carbamidomethylation of cysteines, and variable modifications such as oxidation (M), acetylation (protein N-term), deamidation (NQ), and pyro-glu (N-term Q). Data were processed using Scaffold software with a 1% false discovery rate (FDR) at both the protein and peptide levels, requiring at least two unique peptides per protein.

### Gene ontology analysis

DAVID (Version 2021) [110] (https://david.ncifcrf.gov) was used to perform Gene Ontology analyses. Briefly, gene names were first converted to DAVID-recognizable IDs using Gene Accession Conversion Tool. The updated gene list was then subject to GO analysis using all Homo Sapiens as background and with Functional Annotation Chart function. GO_BP_DIRECT, KEGG_PATHWAY, or UP_KW_BIOLOGICAL_PROCESS were used as GO categories. Only GO terms with a *p* value less than 0.05 were included for further analysis. In addition, for Fig 2C, enrichment analyses were performed using the clusterProfiler R package (v4.14.0). For each of the four gene sets, gene symbols were first converted to Entrez IDs using the bitr function with the mouse annotation database org.Mm.e.g.,db. Genes that failed to map (~3%–6% per group) were excluded. Over-representation analysis (ORA) for GO Biological Process terms was performed using the enrichGO function, and KEGG pathway enrichment was performed using the enrichKEGG function with organism code “mmu”. For both analyses, the complete set of annotated mouse genes (112,288 Entrez IDs) was used as the statistical background universe. *P*-values were adjusted for multiple testing using the Benjamini–Hochberg (BH) method, and terms with adjusted p-value < 0.05 and *q*-value < 0.2 were considered significant. Results were visualized as dot plots using ggplot2, with dot size representing the gene ratio (fraction of input genes annotated to each term) and dot color representing the −log10(adjusted p-value).

### Motif analysis

Motif analysis was performed with the SeqPos motif tool (version 0.590) [111] embedded in Galaxy Cistrome using all motifs within the mouse reference genome mm10 as background. LISA analysis was performed using webtool (http://lisa.cistrome.org/).

### Statistical analysis

Data were analyzed and presented with GraphPad Prism software (version 10.0.0). Exact *p* values were calculated for all statistical analyses unless they were smaller than 1.0e−15, which is the lower limit of detection in GraphPad Prism. In such cases, *p*  <  1.0e−15 is reported. All measurements were taken from distinct biological samples. Plots show individual data points and bars at the mean and ± the standard error of the mean (SEM). Individual tests used were provided at the end of each figure legend. Generally, two-tailed Student’s t-tests were used to compare differences between two groups, except in Fig 4I where strong prior evidence supports a unidirectional change and a subsequent utilization of one-tailed Student *t* test. Two-way ANOVA was used to compare the difference between two treatments with one additional variable (such as different proteostasis genes), such as data in Fig 4D. However, for data with unequal sample size, mixed effects analyses were used in lieu of Two-way ANOVA, such as data in S3A Fig. Cosinor2 was used to assess the presence of 12-h rhythms in different epigenetic marks (Fig 1B), as it is specifically designed for the analysis of short and sparsely sampled time-series data [43].

## Supporting information

S1 Fig ~12 h RBBP5 expression in mouse liver. **(A)** COMPASS writes H3K4me3 using S-adenosyl methionine (SAM) as the substrate. **(B)** LISA revealing putative TFs and co-regulators for hepatic top 500 most robust (the smallest p values by RAIN analysis) circadian (y-axis) and ~12 h (x-axis) genes. **(C)** Temporal murine hepatic *Rbbp5* expression assayed by RNA-seq (*n* = 2 biologically independent samples per time point). Period is calculated by the eigenvalue/pencil method. **(D)** western blot and quantification (*n* = 2 biologically independent samples per time points) of temporal nuclear RBBP5 level in mouse liver at different CTs normalized to total Ponceau S staining intensity. Period is calculated by eigenvalue/pencil method and is further tested against four different methods for the existence of ~12 h rhythm with respective *P* values shown. **(E)** Temporal expression of total RBBP5 proteins in different mice tissues previously reported in [54]. **(F)** Heatmap demonstrates the P values for having 12 h rhythm of RBBP5 expression in each tissue tested by four different methods. Data: Mean ± S.E.M. All data were collected from mice housed under constant darkness conditions with different times indicated by CT. Raw html file for B is available in S2 Raw Image file. Numerical values are available in S1 Data.
(JPG)

S2 Fig  ~12 h RBBP5 cistrome in mouse liver. **(A)** Heatmap showing two independent repeats of temporal RBBP5 ChIP-seq with each row corresponding to the same gene region. **(B)** Heatmap of RBBP5 (repeat 1), H3K9Ac and H3K27Ac chromatin occupancy 1kb ± of TSS of 6,451 genes. **(C)** Snapshot of target genes selected for alignment of hepatic RBBP5 (two repeats), H3K4me3, H3K9Ac, and H3K27Ac at different CTs. **(D)** Heatmap of temporal RBBP5 binding intensity (average of the two repeats) for 7,963 binding sites, along with –log_2_ transformed adjP value for having ~12 h rhythm by RAIN. Those 4,667 ~12 h RBBP5 (adjP < 0.05) sites were highlighted. **(E, F)** Cumulative distribution of the number of ~12 h and circadian RBBP5 binding sites ranked by *p* value (E) or adj P value/false discovery rate (FDR) (F). All data were collected from mice housed under constant darkness conditions with different times indicated by CT. Numerical values are available in S1 Data.
(JPG)

S3 Fig Hepatic RBBP5 cistrome is associated with proteostasis and mRNA metabolic genes. Dot plot for GO analysis demonstrating the −log_10_ transformed adjP value and gene counts of different enriched pathways for different genes. Numerical values are available in S2 Data.
(JPG)

S4 Fig The generation of *Rbbp5* ^*LKO*^ mice. **(A, B)** Expected (A) and actual genotyping result (B) for different mice. **(C, D)** western blot (C) and quantification (D) of RBBP5 in different tissues in *Rbbp5* (fl/fl); Alb-CRE (+/−) and *Rbbp5* (fl/fl); Alb-CRE (−/−) mice. *n* = 2 biologically independent samples per genotype. Data: Mean ± S.E.M. Statistical tests used: unpaired two-tailed Student *t* test for D. Numerical values are available in S3 Data.
(JPG)

S5 Fig Liver hepatocyte-specific deletion of RBBP5 does not alter rhythmic locomotor activity nor fasting-feeding cycles in mice. **(A)** Average body weight of male *Rbbp5* ^*Flox*^ and *Rbbp5* ^*LKO*^ mice at different ages. Mean ± 95% confidence interval. *n* = 3 ~ 71 mice for *Rbbp5* ^*Flox*^ and *n* = 6 ~ 54 mice for *Rbbp5* ^*LKO*^ mice for each week. **(B)** Real-time locomotor activity monitoring in *Rbbp5* ^*Flox*^ and *Rbbp5* ^*LKO*^ mice under 12 hr light/12 hr dark conditions. **(C)** Averaged measurements within the light and dark phase as described in B. **(D)** Real-time measurement of food intake in *Rbbp5* ^*Flox*^ and *Rbbp5* ^*LKO*^ mice under 12 hr light/12 hr dark conditions. **(E)** Averaged measurements within the light and dark phase as described in D. *n* = 6 for *Rbbp5* ^*Flox*^ and *n* = 8 for *Rbbp5* ^*LKO*^ mice. **(F)** Real-time locomotor activity monitoring in *Rbbp5* ^*Flox*^ and *Rbbp5* ^*LKO*^ mice under constant dark conditions. **(G)** Averaged measurements within the rest and active phase as described in F. **(H)** Real-time measurement of food intake in *Rbbp5* ^*Flox*^ and *Rbbp5* ^*LKO*^ mice under constant dark conditions. **(I)** Averaged measurements within the rest and active phase as described in H. *n* = 5 for *Rbbp5* ^*Flox*^ and *n* = 3 for *Rbbp5* ^*LKO*^ mice. Data: Mean ± S.E.M for C, G, E and I. Statistical tests used: unpaired two-tailed Student *t* test for C, G, E and I. Mixed effects analysis for A. Data from F, G, H and I were collected from mice housed under the constant darkness condition with different times indicated by CT. Data from B, C, D, and E were collected from mice housed under the 12 h light on/12 h light off condition, with ZT0 and ZT12 indicating light on and light off time, respectively. Numerical values are available in S3 Data.
(JPG)

S6 Fig RBBP5 is an epigenetic regulator of the hepatic ~12 h oscillator, but not the canonical ~ 24 h circadian clock. **(A)** A table listing the number of ~12 h and ~24 h circadian genes in *Rbbp5* ^*Flox*^ and *Rbbp5* ^*LKO*^ mice with different statistical thresholds. (**B**) Heat map of 6,025 ~12 h gene expression (FDR < 0.05) in the liver of *Rbbp5* ^*Flox*^ and *Rbbp5* ^*LKO*^ mice, with 5,611 of them lost in *Rbbp5* ^*LKO*^ mice, along with −log_10_ transformed adjP values for having 10−12 h rhythm by RAIN. Log_2_ normalized fold change of average gene expression across 48 hours between *Rbbp5* ^*Flox*^ and *Rbbp5* ^*LKO*^ mice was also shown. **(C)** Heat map summary of GO analysis demonstrating the −log_10_ transformed *P* values of different enriched pathways for different genes. All data were collected from mice housed under constant darkness conditions with different times indicated by CT. Numerical values are available in S3 Data.
(JPG)

S7 Fig MetaCycle and cosinor2 methods reveal RBBP5-dependent hepatic  ~12 h transcriptome. **(A-D)** MetaCycle method: Histograms showing the period distributions of all rhythmic genes uncovered from *Rbbp5* ^*Flox*^ and *Rbbp5* ^*LKO*^ mice with P value **(A)** and adjP values **(B)** cut off (<0.05). Cumulative distribution of 10 ~12 h genes with different FDR cut-offs **(C)** and number of 10-12 h and 22h-28h genes in *Rbbp5* ^*Flox*^ and *Rbbp5* ^*LKO*^ mice with FDR cutoff of 0.05 **(D)**. **(E-H)** Cosinor2 method: Histograms showing the period distributions of all rhythmic genes uncovered from *Rbbp5* ^*Flox*^ and *Rbbp5* ^*LKO*^ mice with P value **(E)** and adjP values **(F)** cut off (<0.05). Cumulative distribution of 10 ~ 12 h genes with different FDR cut-offs **(G)** and number of 10-12 h and 22h-28h genes in *Rbbp5* ^*Flox*^ and *Rbbp5* ^*LKO*^ mice with FDR cutoff of 0.05 **(H)**. Numerical values are available in S3 Data.
(JPG)

S8 Fig Eigenvalue/pencil method analysis of RBBP5-dependent hepatic ~12 h transcriptome. **(A, B)** Distributions of all (A) and dominant (B) oscillations uncovered by the eigenvalue/pencil method in *Rbbp5* ^*Flox*^ and *Rbbp5* ^*LKO*^ mice. **(C)** Heat map of all ~12 h gene expressions (or lack thereof) in *Rbbp5* ^*Flox*^ and *Rbbp5* ^*LKO*^ mice with both raw data and superimposed ~12 h rhythms shown. 4,522 ~12 h genes were abolished, 238 ~12 h genes dampened, and 392 ~12 h genes were enhanced in *Rbbp5* ^*LKO*^ mice, respectively. Log_2_ normalized fold change of average gene expression across 48 hours between *Rbbp5* ^*Flox*^ and *Rbbp5* ^*LKO*^ mice was also shown. **(D)** The periods distribution in *Rbbp5* ^*LKO*^ mice of those ~12 h genes originally identified in *Rbbp5* ^*Flox*^ mice but lost in *Rbbp5* ^*LKO*^ mice. **(E)** Venn diagram comparing unique and common ~12 h transcriptomes uncovered by the eigenvalue/pencil and RAIN methods with two different FDR cut-offs. All data were collected from mice housed under constant darkness conditions with different times indicated by CT. Numerical values are available in S3 Data.
(JPG)

S9 Fig RBBP5 is a cell-autonomous epigenetic regulator of the ~12 h oscillator, but not the canonical ~ 24h circadian clock. **(A)** qPCR analysis of *Rbbp5* expression in Tu (25ng/ml)-synchronized MEFs with scrambled, *Bmal1* and *Xbp1* siRNAs. RAIN [55] was used to test for the existence of 12 h rhythms with *P* values shown (*n* = 3 ~ 12 biologically independent samples per time point). **(B)** Representative raw (left) and polynomial detrended (middle) real-time luminescence of MEFs expressing *Manf* promoter-driven dluc [17] transfected with different siRNAs and quantified periods (*n* = 4 ~ 5 biologically independent samples) (right). **(C)** Real-time luminescence traces of Bmal1-dluc U2OS cells transfected with different siRNAs as reported in [58] (*n* = 13, 2 and 2 for scrambled, *RBBP5* and *ASH2L* siRNA, respectively). Data: Mean ± S.E.M. Statistical tests used: RAIN for A, unpaired two-tailed Welch’s *t* test for B, and unpaired two-tailed Student *t* test for C. Numerical values are available in S3 Data.
(JPG)

S10 Fig RBBP5 regulates the hepatic transcriptional response to proteotoxic stress. **(A**−**D)** Volcano plot illustrating the log _2_ normalized fold change versus −log2 transformed adjusted *p* values for different comparisons. **(E)** Scatter plot comparing the log _2_ transformed fold change of gene expression by Tu in *Rbbp5* ^*Flox*^ (x- axis) and *Rbbp5* ^*LKO*^ (y-axis) mice. **(F)** Scatter plot comparing the −log _10_ transformed adjusted *p* values for gene expression induced by Tu in *Rbbp5* ^*Flox*^ (x- axis) and *Rbbp5* ^*LKO*^ (y-axis) mice. **(G)** Venn diagram comparing distinct and common genes induced by Tu in *Rbbp5* ^*Flox*^ and *Rbbp5* ^*LKO*^ mice with FDR < 0.1. **(H)** Heat map summary of GO analysis demonstrating the −log_10_ transformed P values of different enriched pathways for different groups of genes. Numerical values are available in S4 Data.
(JPG)

S11 Fig RBBP5 expression is positively correlated with those involved in proteostasis and mRNA metabolism and negatively associated with those implicated in immune response in human liver. Data are from the human GD-CAT dataset [65].
(JPG)

S12 Fig RBBP5 is required for transcriptional responses to ER stresses. **(A)** Western blot of RBBP5 in MEFs transfected with scrambled or *Rbbp5* siRNA for 48 h. **(B)** qPCR of different genes in MEFs transfected with scrambled, *Rbbp5* or *Xbp1* siRNAs and treated with DMSO or Tu (100 ng/ml) for 8 h (*n* = 2~4 biologically independent samples). **(C)** qPCR of different genes in MEFs transfected with scrambled, *Ash2l* or *Setd1a* siRNAs and treated with DMSO or Tu (100 ng/ml) for 8 h (*n* = 2 ~ 4 biologically independent samples). **(D)** qPCR of different genes in MEFs transfected with scrambled, or the combined *Gcn5* and *Pcaf* siRNAs and treated with DMSO or Tu (100 ng/ml) for 8 h (*n* = 2~6 biologically independent samples). **(E, F)** Isolation and immortalization of *Rbbp5* (fl/fl) ROSA26-CreERT2 (+/+) MEFs (E), which were treated with vehicle (WT) or tamoxifen (*Rbbp5* KO) for 5 or 7 days and genotyping was performed (F) to confirm the deletion of floxed *Rbbp5* allele. (**G**) *Rbbp5* (fl/fl) ROSA26-CreERT2 (+/+) MEFs were treated with tamoxifen for 5 or 7 days and western blot analysis was performed. (**H**) qPCR of different genes in *Rbbp5* (fl/fl) ROSA26-CreERT2 (+/+) MEFs treated with vehicle (WT) or tamoxifen (*Rbbp5* KO) followed by treatment with vehicle control or 1 mM DTT for 5 hours (*n* = 3 biologically independent samples). Data: Mean ± S.E.M. Statistical tests used: Two-way ANOVA for B, C and H, unpaired two-tailed Student *t* test for D. Numerical values are available in S4 Data.
(JPG)

S13 Fig RBBP5 is required for transcriptional responses to nutrition deprivation. **(A)** qPCR analysis of *Rbbp5* (fl/fl) ROSA26-CreERT2 (+/+) MEFs pre-treated with (RBBP5 KO) or without (WT) tamoxifen and then treated with full DMEM media, leucine-free media or EBSS for 16 hours (*n* = 3 ~ 6 biologically independent samples). (**B**) PCA plot of transcriptome of ROSA26-CreERT2 (+/+) MEFs treated with vehicle (WT) or tamoxifen (*Rbbp5* KO) followed by treatment with full DMEM media or EBSS for 16 hours. **(C)** Diagram of the mCherry-GFP-LC3 autophagy reporter. **(D, E)** Representative images (D) and quantification (E) of autophagic flux in mCherry-GFP-LC3-expressing *Rbbp5* (fl/fl) ROSA26-CreERT2 (+/+) MEFs treated with vehicle (WT) or tamoxifen (*Rbbp5* KO) followed by treatment with full DMEM media or leucine-free media for 16 hours (*n* = 18~26 cells per condition). **(F)** western blot of different proteins in *Rbbp5* (fl/fl) ROSA26-CreERT2 (+/+) MEFs treated with vehicle (WT) or tamoxifen (*Rbbp5* KO) followed by treatment with full DMEM media or leucine free media for 16 hours**. (G)** Percentage of cells normalized to before treatment for mCherry-GFP-LC3-expressing *Rbbp5* (fl/fl) ROSA26-CreERT2 (+/+) MEFs treated with vehicle (WT) or tamoxifen (*Rbbp5* KO) followed by treatment with full DMEM media, leucine-free media or EBSS for up to 16 hours (*n* = 3~4 biologically replicates per condition). Data: Mean ± S.E.M. Statistical tests used: Two-way ANOVA for A and G, unpaired two-tailed Student *t* test for E. Numerical values are available in S4 Data.
(JPG)

S14 Fig Proximity labeling reveals H3K4me-associated proteomic architecture of the proteotoxic stress response. (**A**) Imperial blue staining of total biotinylated proteins in MEF treated with 100ng/ml Tu for 0, 2.5, or 8 h before subject to anti-IgG and anti-H3K4me3 antibody-based proximity labeling. Unspecific bands (in a different gel) denoted by asterisks are excluded from mass spectrometry analysis by gel removal. (**B**) A diagram illustrating common and specific subunits of mammalian COMPASS complex. (**C–E**) Heatmap of fold enrichment of different subunits in COMPASS (C), RNAP II (D) or Integrator Complex (E) (over IgG control) at different times after ER stress. Numerical values are available in S4 Data.
(JPG)

S1 Table FPKM quantification of temporal hepatic RBBP5 ChIP-seq in wild-type mouse liver. Tab1: Quantification for repeat 1. Tab 2: Quantification for repeat 2. Tab 3: RAIN quantification for 12 h cycling RBBP5 peaks. Tab 4: Eigenvalue/pencil for 12 h cycling RBBP5 peaks. Tab 5: RAIN quantification for 24 h cycling RBBP5 peaks.
(XLSX)

S2 Table FPKM quantification of temporal hepatic RNA-seq in *Rbbp5* ^*Flox*^ and *Rbbp5* ^*LKO*^ mice. (XLSX)

S3 Table RAIN analysis of 10~12 h hepatic transcriptome identified in *Rbbp5* ^*Flox*^ and *Rbbp5* ^*LKO*^ mice. Tab 1: 10~12 h oscillations identified in *Rbbp5 ^Flox^* mice. Tab 2: 10~12 h oscillations identified in *Rbbp5 ^LKO^* mice.
(XLSX)

S4 Table Eigenvalue/pencil analysis of oscillating hepatic transcriptome identified in *Rbbp5* ^*Flox*^ and *Rbbp5* ^*LKO*^ mice. Tab 1: All oscillations identified in *Rbbp5 ^Flox^* mice. Tab 2: All dominant oscillations identified in *Rbbp5 ^Flox^* mice. Tab 3: All 10~13 h oscillations identified in *Rbbp5 ^Flox^* mice. Tab 4: All dominant 10~13 h oscillations identified in *Rbbp5 ^Flox^* mice. Tab 5: All oscillations identified in *Rbbp5 ^LKO^* mice. Tab 6: All dominant oscillations identified in *Rbbp5 ^LKO^* mice. Tab 7: All 10~13 h oscillations identified in *Rbbp5 ^LKO^* mice. Tab 8: All dominant 10~13 h oscillations identified in *Rbbp5 ^LKO^* mice.
(XLSX)

S5 Table RNA-seq quantification of hepatic transcriptome in *Rbbp5* ^*Flox*^ and *Rbbp5* ^*LKO*^ mice with or without Tu injection. Tab 1: Raw counts. Tab 2: CPM quantification. Tab 3: FKPM quantification.
(XLSX)

S6 Table Differentially expressed gene (DEG) analysis of hepatic transcriptome in *Rbbp5* ^*Flox*^ and *Rbbp5* ^*LKO*^ mice with or without Tu injection. (XLSX)

S7 Table RNA-seq quantification (TPM) of transcriptome in WT and *Rbbp5* KO MEFs in response to EBSS treatment. (XLSX)

S8 Table Differentially expressed gene (DEG) analysis of WT and *Rbbp5* KO MEFs in response to EBSS treatment. (XLSX)

S9 Table Log 2 normalized counts of 239 proteins identified by H3K4me3 proximity labeling in MEFs that showed the highest enrichment at T = 8h. (XLSX)

S1 Raw Images Original images of western blot gels shown in Figs 2G, 3K, 4B, 4G, 4H, 5H, 6B, S1D, S4B, S4C, S12A, S12F, S12G, S13F, and S14A. (PDF)

S2 Raw Image Original html file for LISA analysis shown in S1B, S14. (HTML)

S1 Data Original numerical values for Figs 1B, 1D, 1E, 1F, S1B–S1F and S2D–S2F. (XLSX)

S2 Data Original numerical values for Figs 2B–2E and S3. (XLSX)

S3 Data Original numerical values for Figs 3C–3L, S4D, S5A–S5I, S6B, S6C, S7A, S7B, S7C, S7E, S7F, S7G, S8A–S8D, and S9A–S9C. (XLSX)

S4 Data Original numerical values for Figs 4B–4I, 5A, 5C, 5D, 5E, 5F, 5I, 6D, 6E, S10A–S10F, S10H, S12B, S12C, S12D, S12H, S13A, S13B, S13E, S13G, and S14C–S14E. (XLSX)

## Abbreviations

BH: Benjamini–Hochberg
BSA: bovine serum albumin
DEG: Differentially expressed gene
EBSS: Earle’s Balanced Salt Solution
ER: endoplasmic reticulum
FDR: false discovery rate
GO: Gene Ontology
HAT: histone acetyltransferase
HSF1: heat shock factor 1
IDT: Integrated DNA Technologies
ISR: integrated stress response
iWAT: inguinal white adipose tissue
LISA: Landscape In Silico deletion Analysis
LLPS: liquid-liquid phase separation
MEFs: mouse embryonic fibroblasts
MOI: multiplicity of infection
NE: Nuclear extracts
ORA: Over-representation analysis
PCA: Principle component analysis
SAM: S-adenosyl methionine
SEM: standard error of the mean
TBG: Thyroxine binding globulin
TF: transcription factors
TSS: transcription start sites
UPR: unfolded protein response.

## References

[1] Labbadia J, Morimoto RI. The biology of proteostasis in aging and disease. Annu Rev Biochem. 2015;84:435–64. doi: 10.1146/annurev-biochem-060614-033955 25784053 PMC4539002

[2] Buttgereit F, Brand MD. A hierarchy of ATP-consuming processes in mammalian cells. Biochem J. 1995;312 (Pt 1)(Pt 1):163–7. doi: 10.1042/bj3120163 7492307 PMC1136240

[3] Zhao L, Ackerman SL. Endoplasmic reticulum stress in health and disease. Curr Opin Cell Biol. 2006;18(4):444–52. doi: 10.1016/j.ceb.2006.06.005 16781856

[4] Klaips CL, Jayaraj GG, Hartl FU. Pathways of cellular proteostasis in aging and disease. J Cell Biol. 2018;217(1):51–63. doi: 10.1083/jcb.201709072 29127110 PMC5748993

[5] Martínez G, Duran-Aniotz C, Cabral-Miranda F, Vivar JP, Hetz C. Endoplasmic reticulum proteostasis impairment in aging. Aging Cell. 2017;16(4):615–23. doi: 10.1111/acel.12599 28436203 PMC5506418

[6] Taylor RC, Hetz C. Mastering organismal aging through the endoplasmic reticulum proteostasis network. Aging Cell. 2020;19(11):e13265. doi: 10.1111/acel.13265 33128506 PMC7681052

[7] Costa-Mattioli M, Walter P. The integrated stress response: From mechanism to disease. Science. 2020;368(6489):eaat5314. doi: 10.1126/science.aat5314 32327570 PMC8997189

[8] Hetz C, Zhang K, Kaufman RJ. Mechanisms, regulation and functions of the unfolded protein response. Nat Rev Mol Cell Biol. 2020;21(8):421–38. doi: 10.1038/s41580-020-0250-z 32457508 PMC8867924

[9] Richter K, Haslbeck M, Buchner J. The heat shock response: Life on the verge of death. Mol Cell. 2010;40(2):253–66. doi: 10.1016/j.molcel.2010.10.006 20965420

[10] Ron D, Walter P. Signal integration in the endoplasmic reticulum unfolded protein response. Nat Rev Mol Cell Biol. 2007;8(7):519–29. doi: 10.1038/nrm2199 17565364

[11] Balch WE, Morimoto RI, Dillin A, Kelly JW. Adapting proteostasis for disease intervention. Science. 2008;319(5865):916–9. doi: 10.1126/science.1141448 18276881

[12] Acosta-Alvear D, Harnoss JM, Walter P, Ashkenazi A. Homeostasis control in health and disease by the unfolded protein response. Nat Rev Mol Cell Biol. 2025;26(3):193–212. doi: 10.1038/s41580-024-00794-0 39501044

[13] Zhu B, Dacso CC, O’Malley BW. Unveiling “Musica Universalis” of the cell: A brief history of biological 12-hour rhythms. J Endocr Soc. 2018;2(7):727–52. doi: 10.1210/js.2018-00113 29978151 PMC6025213

[14] Meng H, Gonzales NM, Lonard DM, Putluri N, Zhu B, Dacso CC, et al. XBP1 links the 12-hour clock to NAFLD and regulation of membrane fluidity and lipid homeostasis. Nat Commun. 2020;11(1):6215. doi: 10.1038/s41467-020-20028-z 33277471 PMC7718229

[15] Ballance H, Zhu B. Revealing the hidden reality of the mammalian 12-h ultradian rhythms. Cell Mol Life Sci. 2021;78(7):3127–40. doi: 10.1007/s00018-020-03730-5 33449146 PMC8407301

[16] Meng H, Gonzales NM, Jung SY, Lu Y, Putluri N, Zhu B, et al. Defining the mammalian coactivation of hepatic 12-h clock and lipid metabolism. Cell Rep. 2022;38(10):110491. doi: 10.1016/j.celrep.2022.110491 35263593 PMC8958721

[17] Pan Y, Ballance H, Meng H, Gonzalez N, Kim S-M, Abdurehman L, et al. 12-h clock regulation of genetic information flow by XBP1s. PLoS Biol. 2020;18(1):e3000580. doi: 10.1371/journal.pbio.3000580 31935211 PMC6959563

[18] Zhu B, Liu S, David NL, Dion W, Doshi NK, Siegel LB, et al. Evidence for ~12-h ultradian gene programs in humans. NPJ Biol Timing Sleep. 2024;1(1):4. doi: 10.1038/s44323-024-00005-1 39148626 PMC11325440

[19] Dion W, Zhu B. Basic research and opportunities for translational advancement in the field of mammalian ∼12-hour ultradian chronobiology. Front Physiol. 2024;15:1497836. doi: 10.3389/fphys.2024.1497836 39633646 PMC11614809

[20] Matz-Soja M, Körner C, Ott F, Fischer J, Marbach-Breitrück E, Bergmann C, et al. Modeling the dynamics of hepatic metabolism: The predominance of 12-hour rhythmicity in metabolic adaptation. Cell Mol Life Sci. 2026;83(1):55. doi: 10.1007/s00018-025-06046-4 41511549 PMC12819941

[21] Song J, Wang C, Li H, Wang T, Jiao X. Unveiling the 12-hour ultradian rhythm: biological foundations, mechanistic insights, and potential applications. Cell Biochem Funct. 2026;44(3):e70195. doi: 10.1002/cbf.70195 41845938

[22] Zhu B, Liu S. Preservation of ∼12-h ultradian rhythms of gene expression of mRNA and protein metabolism in the absence of canonical circadian clock. Front Physiol. 2023;14:1195001. doi: 10.3389/fphys.2023.1195001 37324401 PMC10267751

[23] Zhu B. Decoding the function and regulation of the mammalian 12-h clock. J Mol Cell Biol. 2020;12(10):752–8. doi: 10.1093/jmcb/mjaa021 32384155 PMC7816679

[24] Jiao X, Li Z. Temporal dynamics and composition of ocular surface microbiota in C57BL/6J mice: uncovering a 12 h ultradian rhythm. Front Cell Infect Microbiol. 2023;13:1244454. doi: 10.3389/fcimb.2023.1244454 38029247 PMC10651734

[25] Dion W, Ballance H, Lee J, Pan Y, Irfan S, Edwards C, et al. Four-dimensional nuclear speckle phase separation dynamics regulate proteostasis. Sci Adv. 2022;8(1):eabl4150. doi: 10.1126/sciadv.abl4150 34985945 PMC8730402

[26] Scott MR, Zong W, Ketchesin KD, Seney ML, Tseng GC, Zhu B, et al. Twelve-hour rhythms in transcript expression within the human dorsolateral prefrontal cortex are altered in schizophrenia. PLoS Biol. 2023;21(1):e3001688. doi: 10.1371/journal.pbio.3001688 36693045 PMC9873190

[27] Chen L, Chen P, Xie Y, Guo J, Chen R, Guo Y, et al. Twelve-hour ultradian rhythmic reprogramming of gene expression in the human ovary during aging. J Assist Reprod Genet. 2025;42(2):545–61. doi: 10.1007/s10815-024-03339-8 39849236 PMC11871189

[28] Asher G, Zhu B. Beyond circadian rhythms: Emerging roles of ultradian rhythms in control of liver functions. Hepatology. 2023;77(3):1022–35. doi: 10.1002/hep.32580 35591797 PMC9674798

[29] Westermark PO, Herzel H. Mechanism for 12 hr rhythm generation by the circadian clock. Cell Rep. 2013;3(4):1228–38. doi: 10.1016/j.celrep.2013.03.013 23583178

[30] Zhu B, Zhang Q, Pan Y, Mace EM, York B, Antoulas AC, et al. A cell-autonomous mammalian 12 hr clock coordinates metabolic and stress rhythms. Cell metabolism. 2017;25(6):1305–19. e9. 10.1016/j.cmet.2017.05.004 PMC5526350 28591634

[31] Cretenet G, Le Clech M, Gachon F. Circadian clock-coordinated 12 Hr period rhythmic activation of the IRE1alpha pathway controls lipid metabolism in mouse liver. Cell Metab. 2010;11(1):47–57. doi: 10.1016/j.cmet.2009.11.002 20074527

[32] Emery P, Gachon F. Biological rhythms: Living your life, one half-day at a time. NPJ Biol Timing Sleep. 2025;2(1):21. doi: 10.1038/s44323-025-00037-1 40475272 PMC12133581

[33] Hughes ME, DiTacchio L, Hayes KR, Vollmers C, Pulivarthy S, Baggs JE, et al. Harmonics of circadian gene transcription in mammals. PLoS Genet. 2009;5(4):e1000442. doi: 10.1371/journal.pgen.1000442 19343201 PMC2654964

[34] Unruh BA, Weidemann DE, Miao L, Kojima S. Coordination of rhythmic RNA synthesis and degradation orchestrates 24- and 12-h RNA expression patterns in mouse fibroblasts. Proc Natl Acad Sci U S A. 2024;121(7):e2314690121. doi: 10.1073/pnas.2314690121 38315868 PMC10873638

[35] van der Veen DR, Gerkema MP. Unmasking ultradian rhythms in gene expression. FASEB J. 2017;31(2):743–50. doi: 10.1096/fj.201600872R 27871062 PMC5240665

[36] Zhou D, López-Valiente RE, Mattar SG, Guan D. Noncanonical clock regulators control stress responses in digestive diseases. Trends Endocrinol Metab. 2026;37(5):454–65. doi: 10.1016/j.tem.2025.07.002 40753007 PMC12720270

[37] Lago Solis B, Koch R, Nagoshi E. Circadian clock-independent ultradian rhythms in lipid metabolism in the *Drosophila* fat body. J Biol Chem. 2025;301(6):110245. doi: 10.1016/j.jbc.2025.110245 40383146 PMC12205654

[38] Shilatifard A. The COMPASS family of histone H3K4 methylases: Mechanisms of regulation in development and disease pathogenesis. Annu Rev Biochem. 2012;81:65–95. doi: 10.1146/annurev-biochem-051710-134100 22663077 PMC4010150

[39] Dasgupta S, Lonard DM, O’Malley BW. Nuclear receptor coactivators: Master regulators of human health and disease. Annu Rev Med. 2014;65:279–92. doi: 10.1146/annurev-med-051812-145316 24111892 PMC4327818

[40] Roeder RG. Transcriptional regulation and the role of diverse coactivators in animal cells. FEBS Lett. 2005;579(4):909–15. doi: 10.1016/j.febslet.2004.12.007 15680973

[41] Spiegelman BM, Heinrich R. Biological control through regulated transcriptional coactivators. Cell. 2004;119(2):157–67. doi: 10.1016/j.cell.2004.09.037 15479634

[42] Koike N, Yoo S-H, Huang H-C, Kumar V, Lee C, Kim T-K, et al. Transcriptional architecture and chromatin landscape of the core circadian clock in mammals. Science. 2012;338(6105):349–54. doi: 10.1126/science.1226339 22936566 PMC3694775

[43] Cornelissen G. Cosinor-based rhythmometry. Theor Biol Med Model. 2014;11:16. doi: 10.1186/1742-4682-11-16 24725531 PMC3991883

[44] Takahashi Y, Westfield GH, Oleskie AN, Trievel RC, Shilatifard A, Skiniotis G. Structural analysis of the core COMPASS family of histone H3K4 methylases from yeast to human. Proc Natl Acad Sci U S A. 2011;108(51):20526–31. doi: 10.1073/pnas.1109360108 22158900 PMC3251153

[45] Shilatifard A. The COMPASS family of histone H3K4 methylases: Mechanisms of regulation in development and disease pathogenesis. Annu Rev Biochem. 2012;81:65–95. doi: 10.1146/annurev-biochem-051710-134100 22663077 PMC4010150

[46] Qu Q, Takahashi Y-H, Yang Y, Hu H, Zhang Y, Brunzelle JS, et al. Structure and conformational dynamics of a COMPASS histone H3K4 methyltransferase complex. Cell. 2018;174(5):1117-1126.e12. doi: 10.1016/j.cell.2018.07.020 30100186 PMC6108936

[47] Hu S, Song A, Peng L, Tang N, Qiao Z, Wang Z, et al. H3K4me2/3 modulate the stability of RNA polymerase II pausing. Cell Res. 2023;33(5):403–6. doi: 10.1038/s41422-023-00794-3 36922644 PMC10156655

[48] Wang H, Fan Z, Shliaha PV, Miele M, Hendrickson RC, Jiang X, et al. H3K4me3 regulates RNA polymerase II promoter-proximal pause-release. Nature. 2023;615(7951):339–48. doi: 10.1038/s41586-023-05780-8 36859550 PMC9995272

[49] Qin Q, Fan J, Zheng R, Wan C, Mei S, Wu Q, et al. Lisa: inferring transcriptional regulators through integrative modeling of public chromatin accessibility and ChIP-seq data. Genome Biol. 2020;21(1):32. doi: 10.1186/s13059-020-1934-6 32033573 PMC7007693

[50] Li Y, Han J, Zhang Y, Cao F, Liu Z, Li S, et al. Structural basis for activity regulation of MLL family methyltransferases. Nature. 2016;530(7591):447–52. doi: 10.1038/nature16952 26886794 PMC5125619

[51] Ruthenburg AJ, Allis CD, Wysocka J. Methylation of lysine 4 on histone H3: intricacy of writing and reading a single epigenetic mark. Mol Cell. 2007;25(1):15–30. doi: 10.1016/j.molcel.2006.12.014 17218268

[52] Han J, Li T, Li Y, Li M, Wang X, Peng C, et al. The internal interaction in RBBP5 regulates assembly and activity of MLL1 methyltransferase complex. Nucleic Acids Res. 2019;47(19):10426–38. doi: 10.1093/nar/gkz819 31544921 PMC6821195

[53] Antoulas AC, Zhu B, Zhang Q, York B, O’Malley BW, Dacso CC. A novel mathematical method for disclosing oscillations in gene transcription: A comparative study. PLoS One. 2018;13(9):e0198503. doi: 10.1371/journal.pone.0198503 30231032 PMC6145530

[54] Otobe Y, Deki-Arima N, Xinyan S, Itabashi K, Kurabayashi N, Nakamura U, et al. A mouse circadian proteome atlas. Mol Cell. 2026;86(2):393-406.e3. doi: 10.1016/j.molcel.2025.12.020 41519126

[55] Thaben PF, Westermark PO. Detecting rhythms in time series with RAIN. J Biol Rhythms. 2014;29(6):391–400. doi: 10.1177/0748730414553029 25326247 PMC4266694

[56] Zhu B, Liu S. Preservation of ∼12-h ultradian rhythms of gene expression of mRNA and protein metabolism in the absence of canonical circadian clock. Front Physiol. 2023;14:1195001. doi: 10.3389/fphys.2023.1195001 37324401 PMC10267751

[57] Wu G, Anafi RC, Hughes ME, Kornacker K, Hogenesch JB. MetaCycle: An integrated R package to evaluate periodicity in large scale data. Bioinformatics. 2016;32(21):3351–3. doi: 10.1093/bioinformatics/btw405 27378304 PMC5079475

[58] Zhang EE, Liu AC, Hirota T, Miraglia LJ, Welch G, Pongsawakul PY, et al. A genome-wide RNAi screen for modifiers of the circadian clock in human cells. Cell. 2009;139(1):199–210. doi: 10.1016/j.cell.2009.08.031 19765810 PMC2777987

[59] Walter P, Ron D. The unfolded protein response: from stress pathway to homeostatic regulation. Science. 2011;334(6059):1081–6. doi: 10.1126/science.1209038 22116877

[60] Metcalf MG, Higuchi-Sanabria R, Garcia G, Tsui CK, Dillin A. Beyond the cell factory: Homeostatic regulation of and by the UPRER. Sci Adv. 2020;6(29):eabb9614. doi: 10.1126/sciadv.abb9614 32832649 PMC7439504

[61] Wiseman RL, Mesgarzadeh JS, Hendershot LM. Reshaping endoplasmic reticulum quality control through the unfolded protein response. Mol Cell. 2022;82(8):1477–91. doi: 10.1016/j.molcel.2022.03.025 35452616 PMC9038009

[62] So J-S. Roles of endoplasmic reticulum stress in immune responses. Mol Cells. 2018;41(8):705–16. doi: 10.14348/molcells.2018.0241 30078231 PMC6125421

[63] Rutkowski DT, Wu J, Back S-H, Callaghan MU, Ferris SP, Iqbal J, et al. UPR pathways combine to prevent hepatic steatosis caused by ER stress-mediated suppression of transcriptional master regulators. Dev Cell. 2008;15(6):829–40. doi: 10.1016/j.devcel.2008.10.015 19081072 PMC2923556

[64] Yan Z, Yan H, Ou H. Human thyroxine binding globulin (TBG) promoter directs efficient and sustaining transgene expression in liver-specific pattern. Gene. 2012;506(2):289–94. doi: 10.1016/j.gene.2012.07.009 22820390

[65] Zhou M, Tamburini I, Van C, Molendijk J, Nguyen CM, Chang IY-Y, et al. Leveraging inter-individual transcriptional correlation structure to infer discrete signaling mechanisms across metabolic tissues. Elife. 2024;12:RP88863. doi: 10.7554/eLife.88863 38224289 PMC10945578

[66] Gates LA, Shi J, Rohira AD, Feng Q, Zhu B, Bedford MT, et al. Acetylation on histone H3 lysine 9 mediates a switch from transcription initiation to elongation. J Biol Chem. 2017;292(35):14456–72. doi: 10.1074/jbc.M117.802074 28717009 PMC5582839

[67] Higa A, Taouji S, Lhomond S, Jensen D, Fernandez-Zapico ME, Simpson JC, et al. Endoplasmic reticulum stress-activated transcription factor ATF6α requires the disulfide isomerase PDIA5 to modulate chemoresistance. Mol Cell Biol. 2014;34(10):1839–49. doi: 10.1128/MCB.01484-13 24636989 PMC4019026

[68] G G, Singh J. Dithiothreitol causes toxicity in *C. elegans* by modulating the methionine-homocysteine cycle. Elife. 2022;11:e76021. doi: 10.7554/eLife.76021 35438636 PMC9090326

[69] Pakos-Zebrucka K, Koryga I, Mnich K, Ljujic M, Samali A, Gorman AM. The integrated stress response. EMBO Rep. 2016;17(10):1374–95. doi: 10.15252/embr.201642195 27629041 PMC5048378

[70] Bjørkøy G, Lamark T, Pankiv S, Øvervatn A, Brech A, Johansen T. Monitoring autophagic degradation of p62/SQSTM1. Methods Enzymol. 2009;452:181–97. doi: 10.1016/s0076-6879(08)03612-4 19200883

[71] Liang JR, Lingeman E, Luong T, Ahmed S, Muhar M, Nguyen T, et al. A Genome-wide ER-phagy screen highlights key roles of mitochondrial metabolism and ER-resident UFMylation. Cell. 2020;180(6):1160-1177.e20. doi: 10.1016/j.cell.2020.02.017 32160526 PMC7197389

[72] Liang JR, Lingeman E, Ahmed S, Corn JE. Atlastins remodel the endoplasmic reticulum for selective autophagy. J Cell Biol. 2018;217(10):3354–67. doi: 10.1083/jcb.201804185 30143524 PMC6168278

[73] Wang H, Helin K. Roles of H3K4 methylation in biology and disease. Trends Cell Biol. 2025;35(2):115–28. doi: 10.1016/j.tcb.2024.06.001 38909006

[74] Santos-Barriopedro I, van Mierlo G, Vermeulen M. Off-the-shelf proximity biotinylation for interaction proteomics. Nat Commun. 2021;12(1):5015. doi: 10.1038/s41467-021-25338-4 34408139 PMC8373943

[75] Hodges C, Kirkland JG, Crabtree GR. The Many Roles of BAF (mSWI/SNF) and PBAF complexes in cancer. Cold Spring Harb Perspect Med. 2016;6(8):a026930. doi: 10.1101/cshperspect.a026930 27413115 PMC4968166

[76] Masri S, Kinouchi K, Sassone-Corsi P. Circadian clocks, epigenetics, and cancer. Curr Opin Oncol. 2015;27(1):50–6. doi: 10.1097/CCO.0000000000000153 25405464 PMC4732884

[77] Takahashi JS. Transcriptional architecture of the mammalian circadian clock. Nat Rev Genet. 2017;18(3):164–79. doi: 10.1038/nrg.2016.150 27990019 PMC5501165

[78] Nakahata Y, Kaluzova M, Grimaldi B, Sahar S, Hirayama J, Chen D. The NAD-dependent deacetylase SIRT1 modulates CLOCK-mediated chromatin remodeling and circadian control. Cell. 2008;134(2):329–40. doi: 10.1016/j.cell.2008.07.002 PMC3526943 18662547

[79] Aguilar-Arnal L, Sassone-Corsi P. Chromatin landscape and circadian dynamics: Spatial and temporal organization of clock transcription. Proc Natl Acad Sci U S A. 2015;112(22):6863–70. doi: 10.1073/pnas.1411264111 25378702 PMC4460512

[80] Zhu B, Gates LA, Stashi E, Dasgupta S, Gonzales N, Dean A, et al. Coactivator-dependent oscillation of chromatin accessibility dictates circadian gene amplitude via REV-ERB loading. Mol Cell. 2015;60(5):769–83. doi: 10.1016/j.molcel.2015.10.024 26611104 PMC4671835

[81] Stashi E, Lanz RB, Mao J, Michailidis G, Zhu B, Kettner NM, et al. SRC-2 is an essential coactivator for orchestrating metabolism and circadian rhythm. Cell Rep. 2014;6(4):633–45. doi: 10.1016/j.celrep.2014.01.027 24529706 PMC4096300

[82] Doi M, Hirayama J, Sassone-Corsi P. Circadian regulator CLOCK is a histone acetyltransferase. Cell. 2006;125(3):497–508. doi: 10.1016/j.cell.2006.03.033 16678094

[83] Feng D, Liu T, Sun Z, Bugge A, Mullican SE, Alenghat T, et al. A circadian rhythm orchestrated by histone deacetylase 3 controls hepatic lipid metabolism. Science. 2011;331(6022):1315–9. doi: 10.1126/science.1198125 21393543 PMC3389392

[84] Alenghat T, Meyers K, Mullican SE, Leitner K, Adeniji-Adele A, Avila J, et al. Nuclear receptor corepressor and histone deacetylase 3 govern circadian metabolic physiology. Nature. 2008;456(7224):997–1000. doi: 10.1038/nature07541 19037247 PMC2742159

[85] Chen Y-JC, Koutelou E, Dent SYR. Now open: Evolving insights to the roles of lysine acetylation in chromatin organization and function. Mol Cell. 2022;82(4):716–27. doi: 10.1016/j.molcel.2021.12.004 35016034 PMC8857060

[86] Gates LA, Shi J, Rohira AD, Feng Q, Zhu B, Bedford MT, et al. Acetylation on histone H3 lysine 9 mediates a switch from transcription initiation to elongation. J Biol Chem. 2017;292(35):14456–72. doi: 10.1074/jbc.M117.802074 28717009 PMC5582839

[87] Gates LA, Foulds CE, O’Malley BW. Histone marks in the “Driver’s Seat”: Functional roles in steering the transcription cycle. Trends Biochem Sci. 2017;42(12):977–89. doi: 10.1016/j.tibs.2017.10.004 29122461 PMC5701853

[88] Policarpi C, Munafò M, Tsagkris S, Carlini V, Hackett JA. Systematic epigenome editing captures the context-dependent instructive function of chromatin modifications. Nat Genet. 2024;56(6):1168–80. doi: 10.1038/s41588-024-01706-w 38724747 PMC11176084

[89] Mazina MY, Nikolenko YV, Krasnov AN, Vorobyeva NE. SWI/SNF protein complexes participate in the initiation and elongation stages of *Drosophila* hsp70 gene transcription. Genetika. 2016;52(2):164–9. doi: 10.1134/s1022795416010105 27215030

[90] Schwabish MA, Struhl K. The Swi/Snf complex is important for histone eviction during transcriptional activation and RNA polymerase II elongation in vivo. Mol Cell Biol. 2007;27(20):6987–95. doi: 10.1128/MCB.00717-07 17709398 PMC2168902

[91] Cenik BK, Shilatifard A. COMPASS and SWI/SNF complexes in development and disease. Nat Rev Genet. 2021;22(1):38–58. doi: 10.1038/s41576-020-0278-0 32958894

[92] Huang Y, Jay KL, Yen-Wen Huang A, Wan J, Jangam SV, Chorin O, et al. Loss-of-function in RBBP5 results in a syndromic neurodevelopmental disorder associated with microcephaly. Genet Med. 2024;26(11):101218. doi: 10.1016/j.gim.2024.101218 39036895 PMC11648989

[93] Zhu B. Logic of the temporal compartmentalization of the hepatic metabolic cycle. Physiology (Bethesda). 2022;37(5):0. doi: 10.1152/physiol.00003.2022 35658626 PMC9394779

[94] Zhang L, Zhang Y, Chen Y, Gholamalamdari O, Wang Y, Ma J, et al. TSA-seq reveals a largely conserved genome organization relative to nuclear speckles with small position changes tightly correlated with gene expression changes. Genome Res. 2021;31(2):251–64. doi: 10.1101/gr.266239.120 33355299 PMC7849416

[95] Wu J, Xiao Y, Liu Y, Wen L, Jin C, Liu S, et al. Dynamics of RNA localization to nuclear speckles are connected to splicing efficiency. Sci Adv. 2024;10(42):eadp7727. doi: 10.1126/sciadv.adp7727 39413186 PMC11482332

[96] Dion W, Tao Y, Chambers M, Zhao S, Arbuckle RK, Sun M, et al. SON-dependent nuclear speckle rehabilitation alleviates proteinopathies. Nat Commun. 2025;16(1):7065. doi: 10.1038/s41467-025-62242-7 40764481 PMC12325801

[97] Chen X, Zhu B. Decoding the functions of nuclear speckles in neurodegeneration. Trends Neurosci. 2026;49(4):292–305. doi: 10.1016/j.tins.2026.01.010 41794640 PMC13179099

[98] Wulansari N, Darsono WHW, Woo H-J, Chang M-Y, Kim J, Bae E-J, et al. Neurodevelopmental defects and neurodegenerative phenotypes in human brain organoids carrying Parkinson’s disease-linked DNAJC6 mutations. Sci Adv. 2021;7(8):eabb1540. doi: 10.1126/sciadv.abb1540 33597231 PMC7888924

[99] Quadros RM, Miura H, Harms DW, Akatsuka H, Sato T, Aida T, et al. Easi-CRISPR: a robust method for one-step generation of mice carrying conditional and insertion alleles using long ssDNA donors and CRISPR ribonucleoproteins. Genome Biol. 2017;18(1):92. doi: 10.1186/s13059-017-1220-4 28511701 PMC5434640

[100] Pelletier S, Gingras S, Green DR. Mouse genome engineering via CRISPR-Cas9 for study of immune function. Immunity. 2015;42(1):18–27. doi: 10.1016/j.immuni.2015.01.004 25607456 PMC4720985

[101] Xu J. Preparation, culture, and immortalization of mouse embryonic fibroblasts. Curr Protoc Mol Biol. 2005. p. Unit 28.1. doi: 10.1002/0471142727.mb2801s70 18265366

[102] Malovannaya A, Lanz RB, Jung SY, Bulynko Y, Le NT, Chan DW, et al. Analysis of the human endogenous coregulator complexome. Cell. 2011;145(5):787–99. doi: 10.1016/j.cell.2011.05.006 21620140 PMC3131083

[103] Bolger AM, Lohse M, Usadel B. Trimmomatic: A flexible trimmer for Illumina sequence data. Bioinformatics. 2014;30(15):2114–20. doi: 10.1093/bioinformatics/btu170 24695404 PMC4103590

[104] Kim D, Langmead B, Salzberg SL. HISAT: A fast spliced aligner with low memory requirements. Nat Methods. 2015;12(4):357–60. doi: 10.1038/nmeth.3317 25751142 PMC4655817

[105] Liu S, Obert C, Yu Y-P, Zhao J, Ren B-G, Liu J-J, et al. Utility analyses of AVITI sequencing chemistry. BMC Genomics. 2024;25(1):778. doi: 10.1186/s12864-024-10686-4 39127634 PMC11316309

[106] Dobin A, Davis CA, Schlesinger F, Drenkow J, Zaleski C, Jha S, et al. STAR: Ultrafast universal RNA-seq aligner. Bioinformatics. 2013;29(1):15–21. doi: 10.1093/bioinformatics/bts635 23104886 PMC3530905

[107] Trapnell C, Williams BA, Pertea G, Mortazavi A, Kwan G, van Baren MJ, et al. Transcript assembly and quantification by RNA-Seq reveals unannotated transcripts and isoform switching during cell differentiation. Nat Biotechnol. 2010;28(5):511–5. doi: 10.1038/nbt.1621 20436464 PMC3146043

[108] Ge SX, Son EW, Yao R. iDEP: An integrated web application for differential expression and pathway analysis of RNA-Seq data. BMC Bioinformatics. 2018;19(1):534. doi: 10.1186/s12859-018-2486-6 30567491 PMC6299935

[109] Ramírez F, Ryan DP, Grüning B, Bhardwaj V, Kilpert F, Richter AS, et al. deepTools2: A next generation web server for deep-sequencing data analysis. Nucleic Acids Res. 2016;44(W1):W160-5. doi: 10.1093/nar/gkw257 27079975 PMC4987876

[110] Huang DW, Sherman BT, Lempicki RA. Systematic and integrative analysis of large gene lists using DAVID bioinformatics resources. Nat Protoc. 2009;4(1):44–57. doi: 10.1038/nprot.2008.211 19131956

[111] He HH, Meyer CA, Shin H, Bailey ST, Wei G, Wang Q, et al. Nucleosome dynamics define transcriptional enhancers. Nat Genet. 2010;42(4):343–7. doi: 10.1038/ng.545 20208536 PMC2932437

