# Supplementary material for: The histone methyltransferase complex subunit RBBP5 serves as a central epigenetic regulator of proteostasis dynamics
Source: PLoS Biol. 2026 Oct 8;24(10):e3004048. doi: 10.1371/journal.pbio.3004048 (PMC13667922; doi:10.1371/journal.pbio.3004048)
Supplement: Supplementary file 1 [file pbio.3004048.s024.pdf]

Fig. S1D

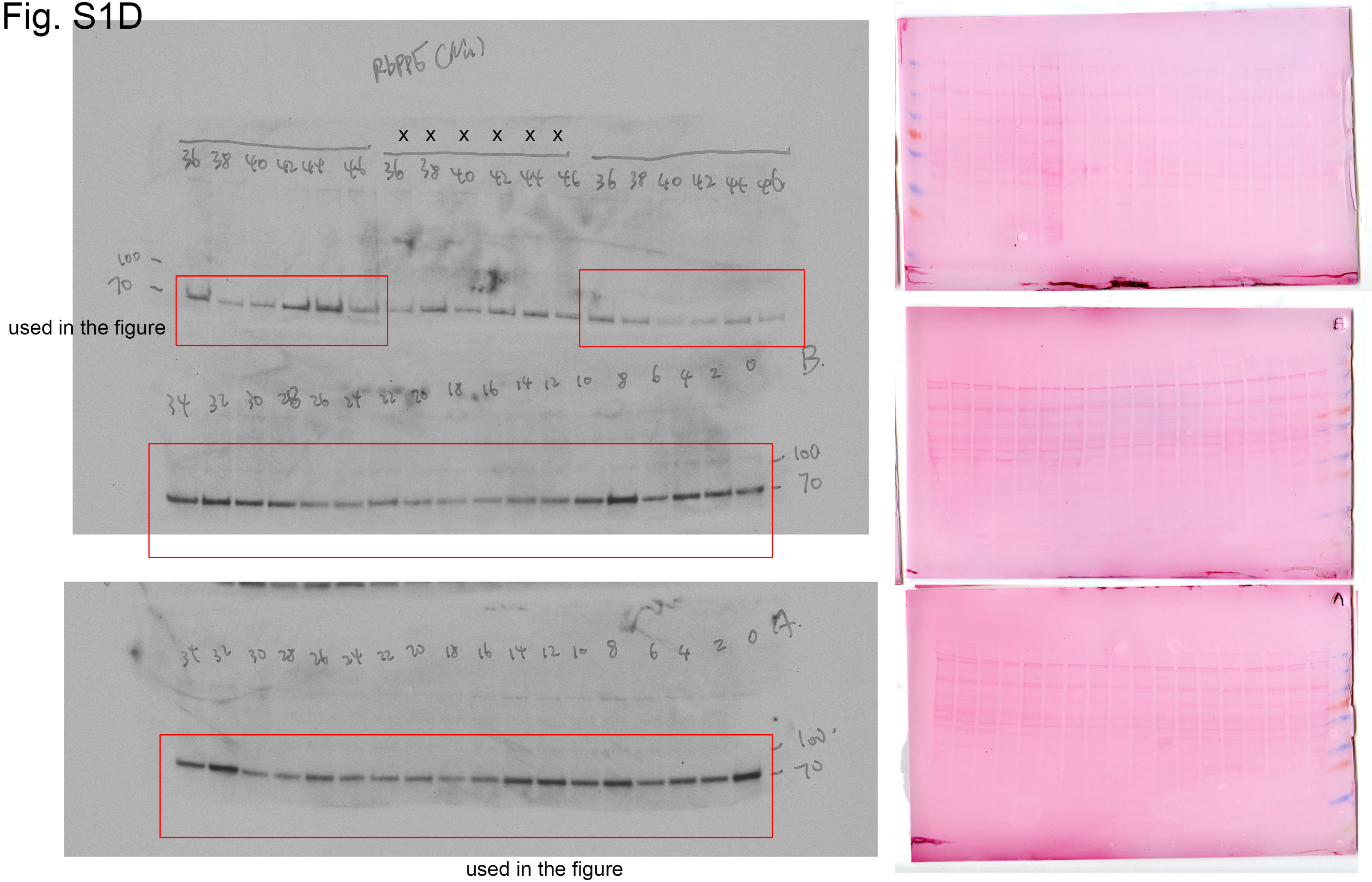

Fig 4B

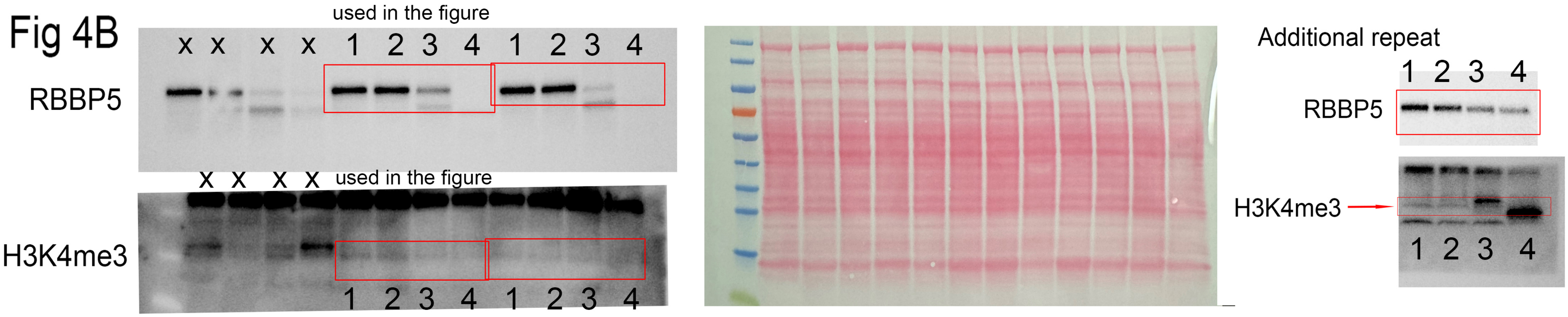

1. WT DMSO 2. WT Tu 3. Rbbp5 LKO DMSO 4.Rbbp5 LKO Tu

Fig 4G

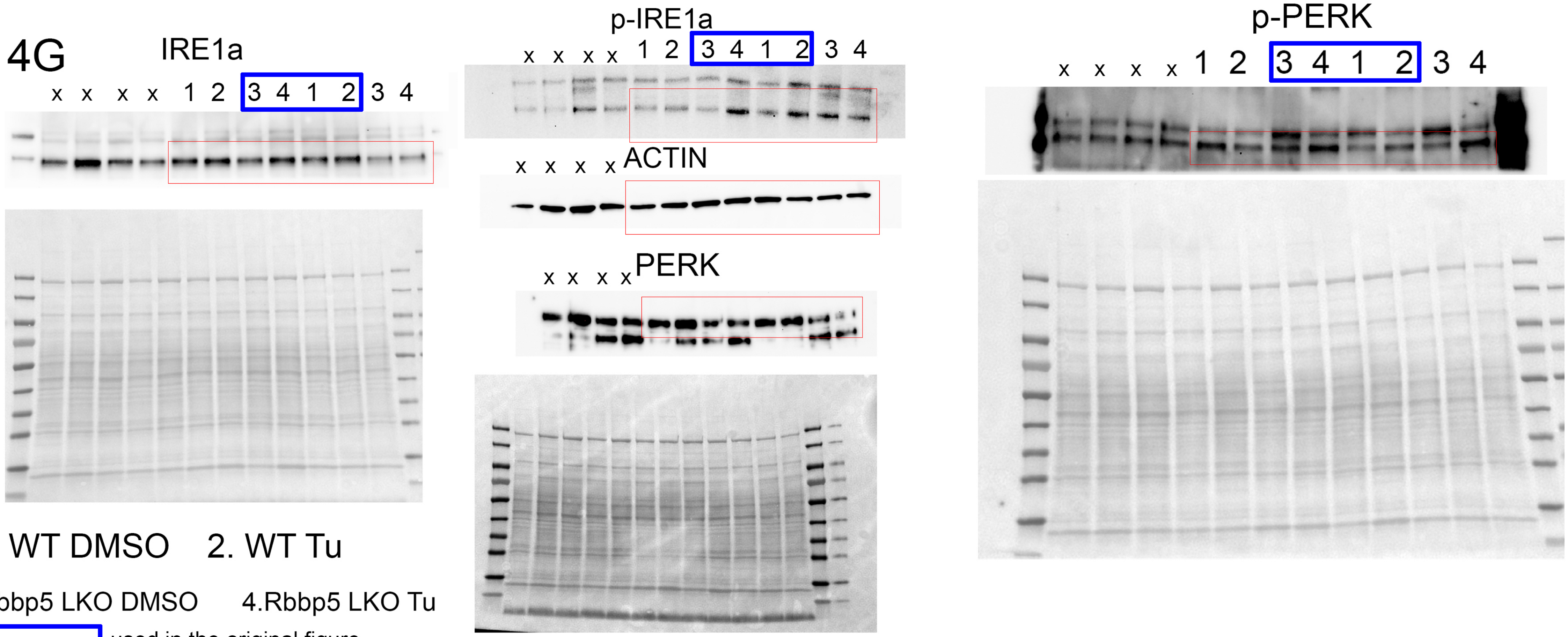

1. WT DMSO 2. WT Tu

3. Rbbp5 LKO DMSO 4.Rbbp5 LKO Tu

used in the original figure

Fig. 4H

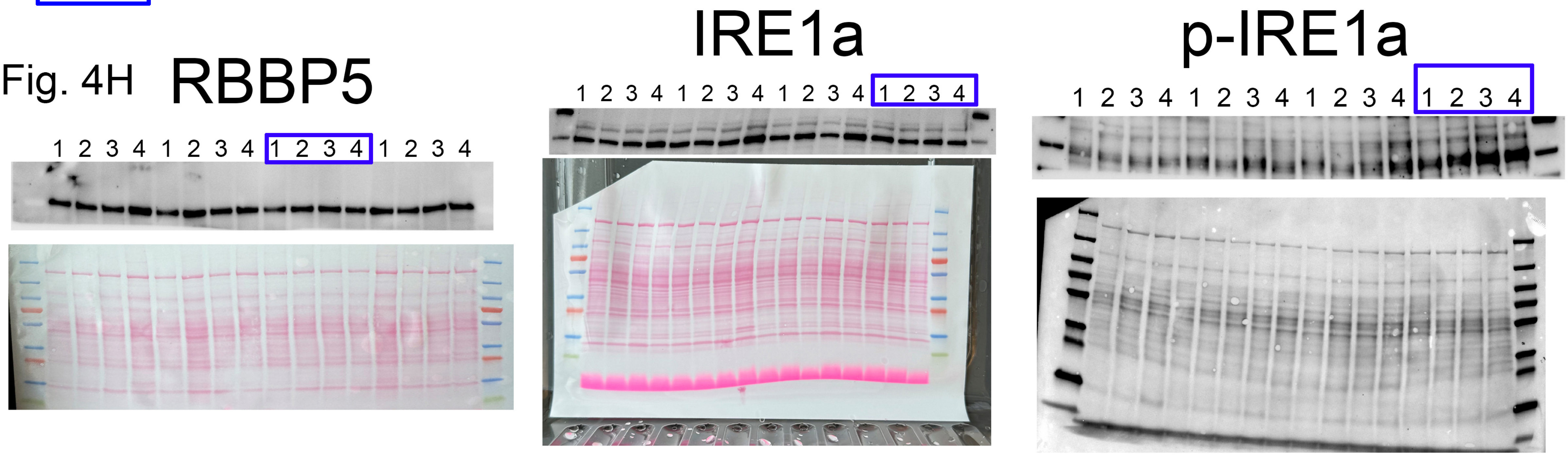

1.Ad-GFP DMSO 2. Ad-GFP Tu 3. Ad-RBBP5 DMSO 4. Ad-RBBP5 Tu

used in the original figure

Fig. S12A

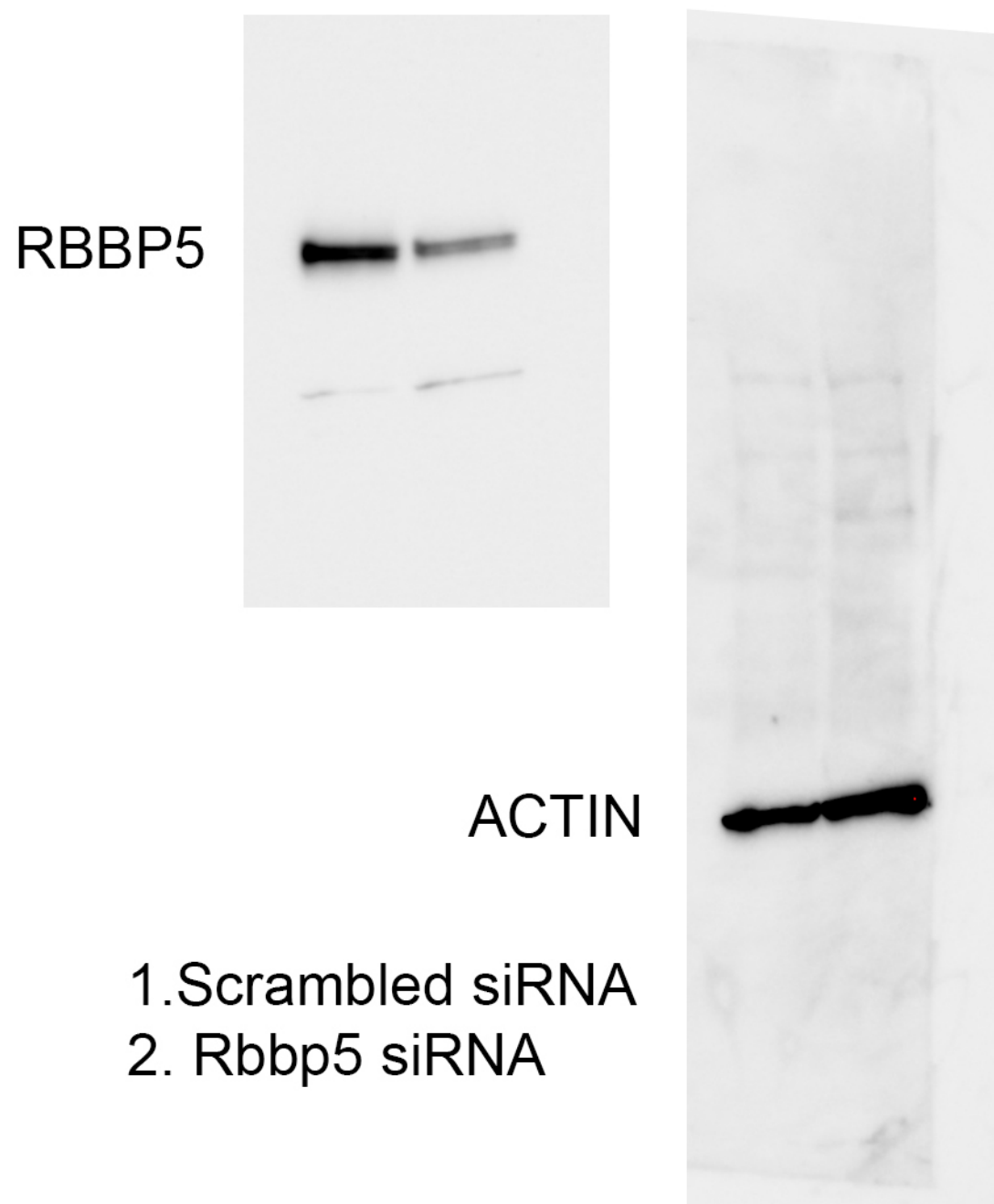

Fig. S12G

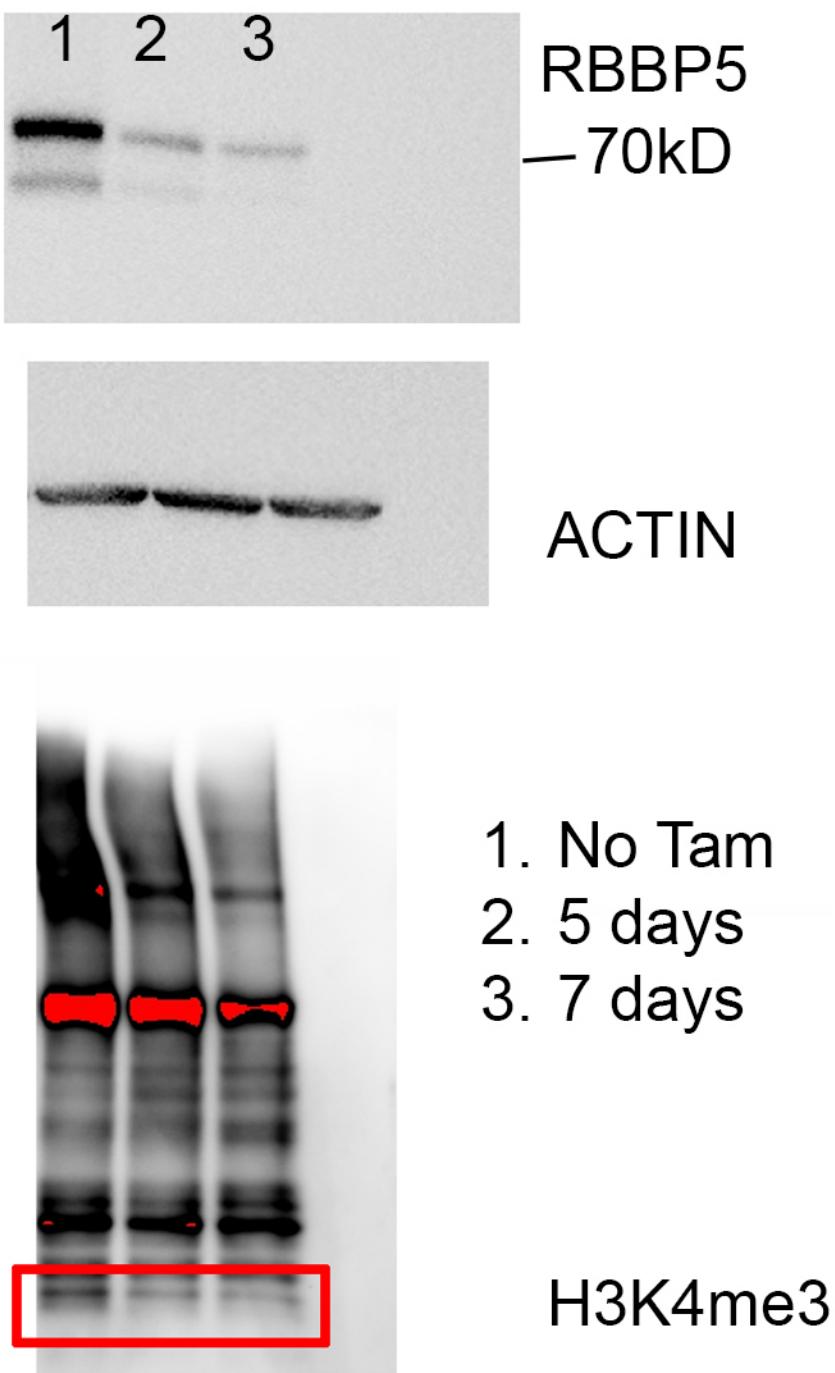

Fig. S13F

1. WT full medium
2. RBBP5 KO full medium
3. WT leucine free
4. RBBP5 KO leucine free

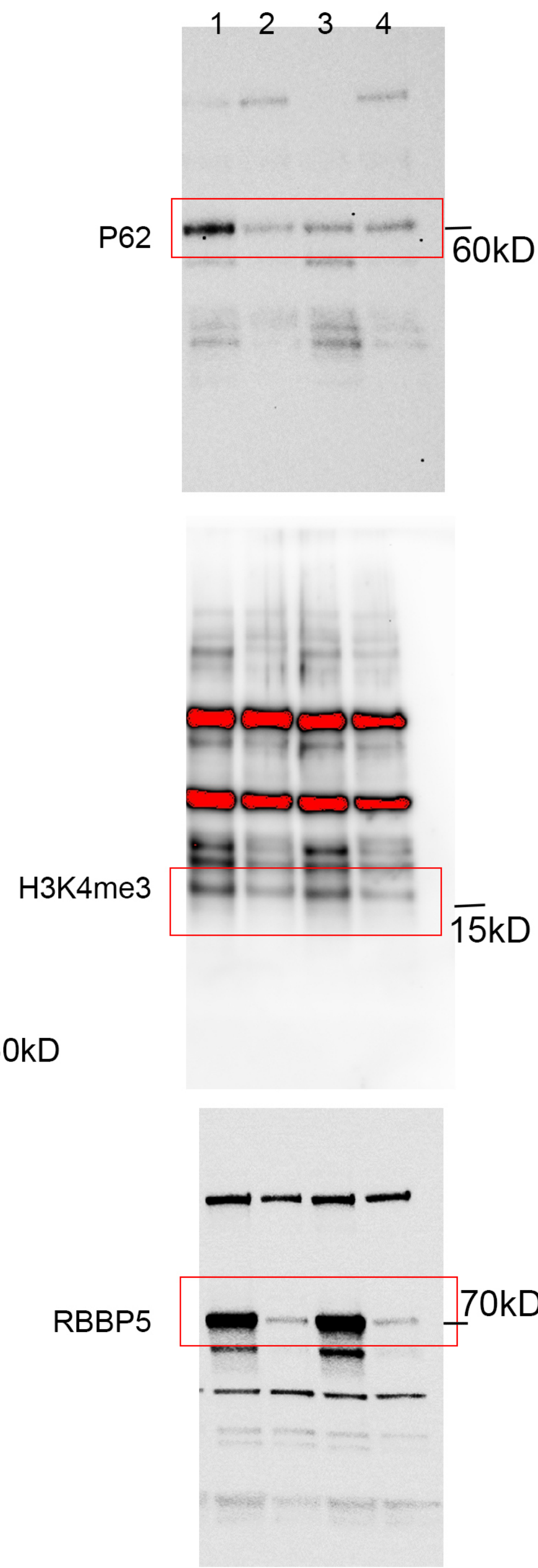

Fig. 5H

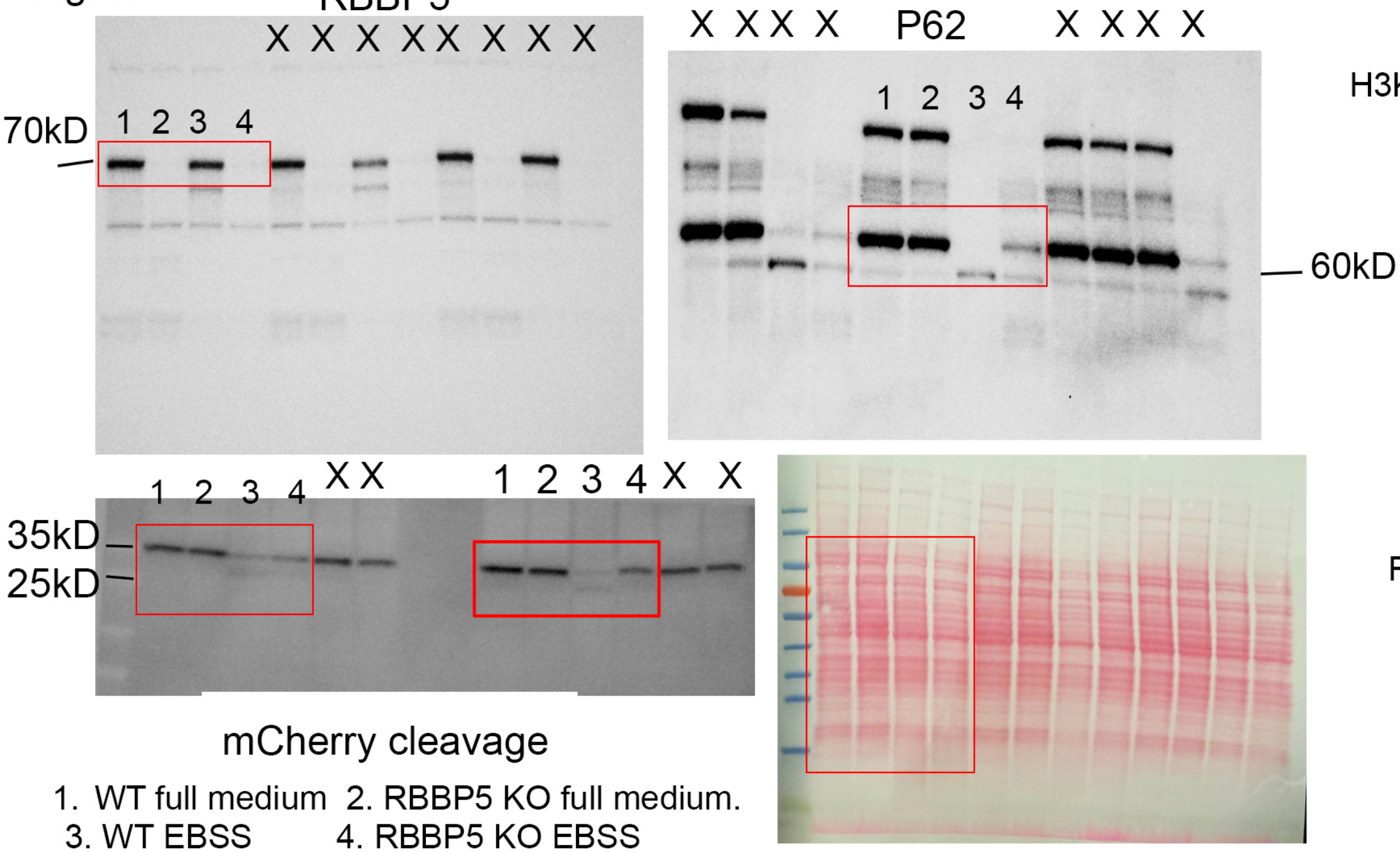

mCherry cleavage

1. WT full medium 2. RBBP5 KO full medium.
3. WT EBSS 4. RBBP5 KO EBSS

Fig 2G

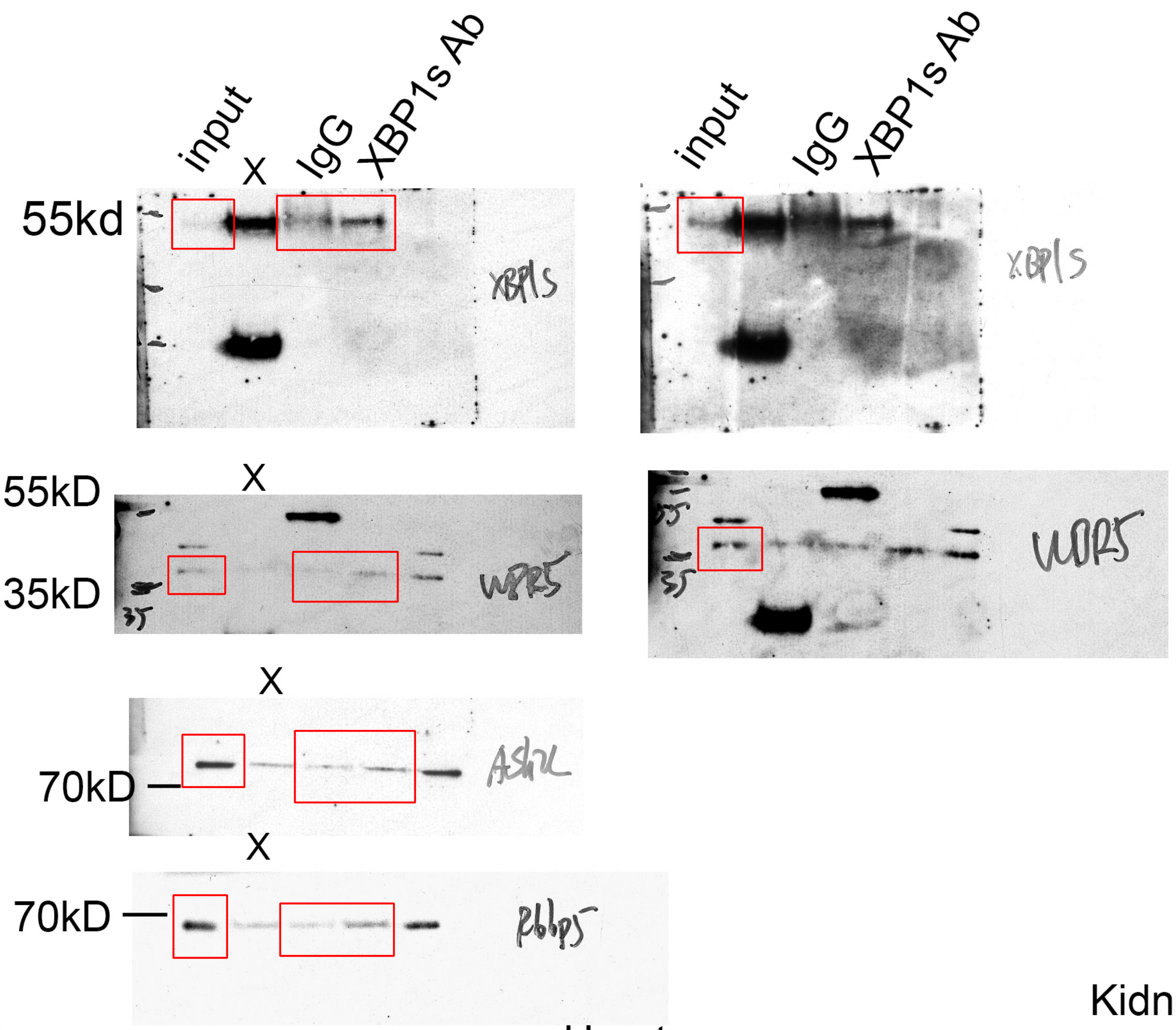

Fig S4C

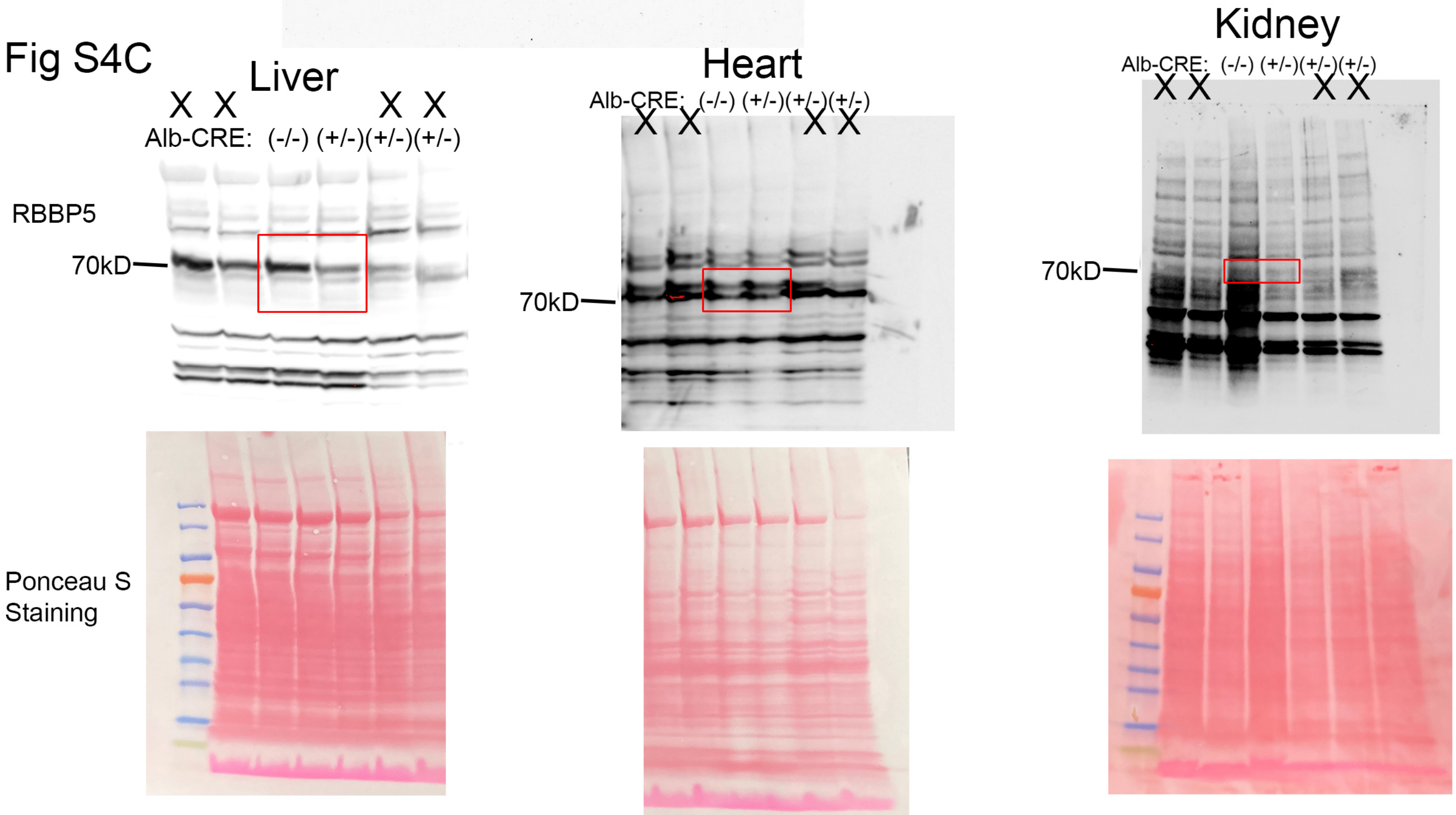

Fig. 6B.

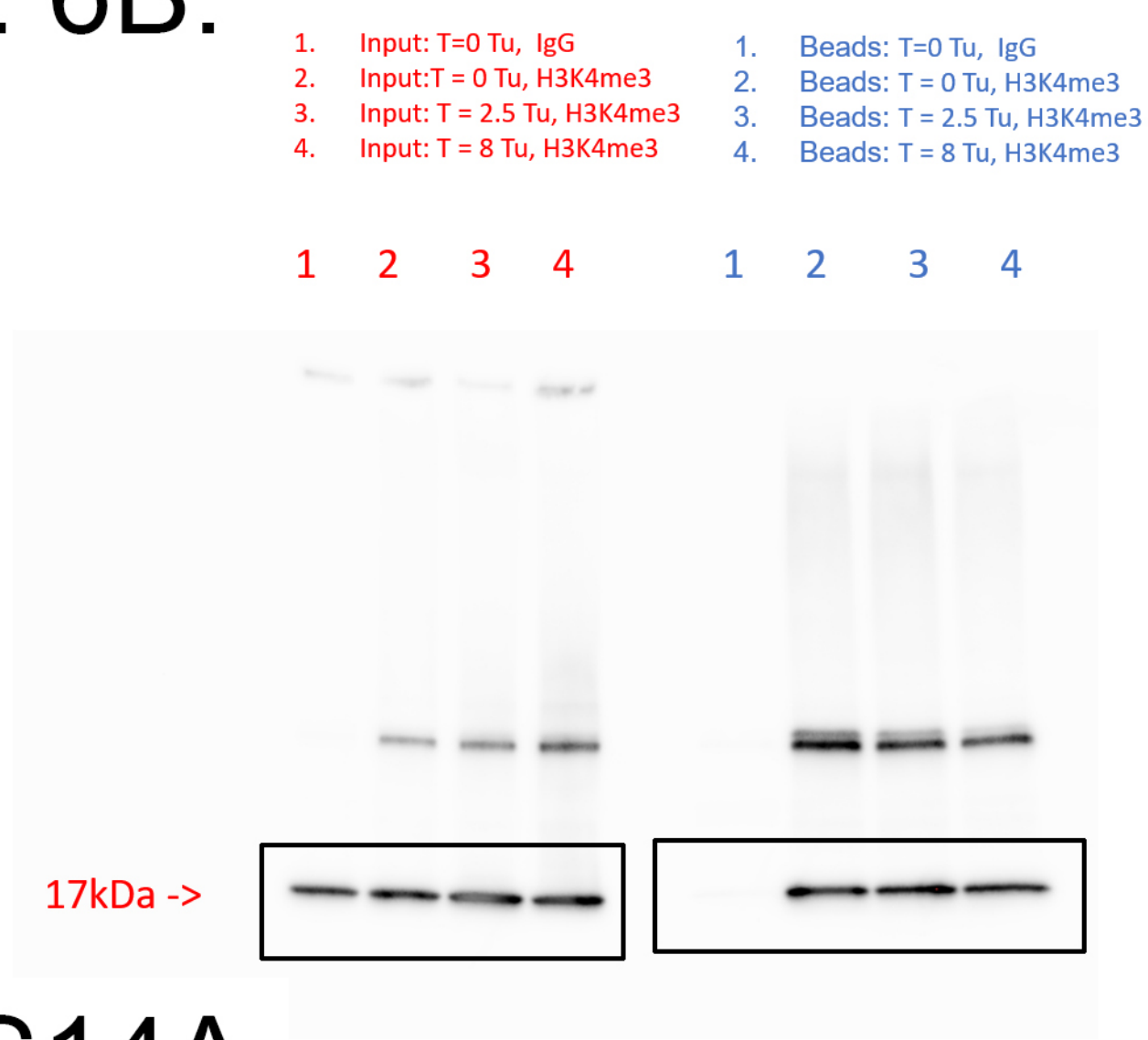

Fig. S12F

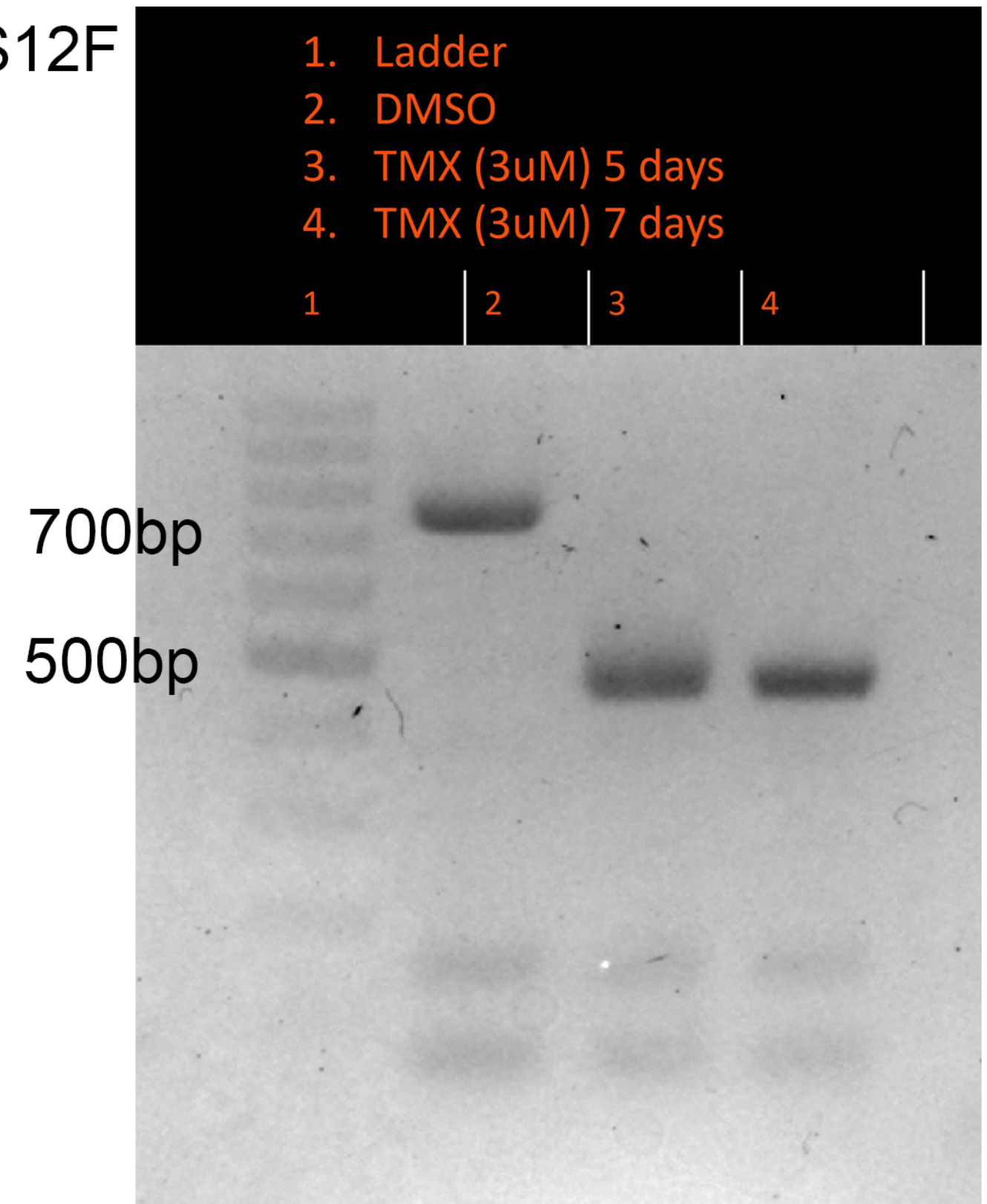

Fig. S14A

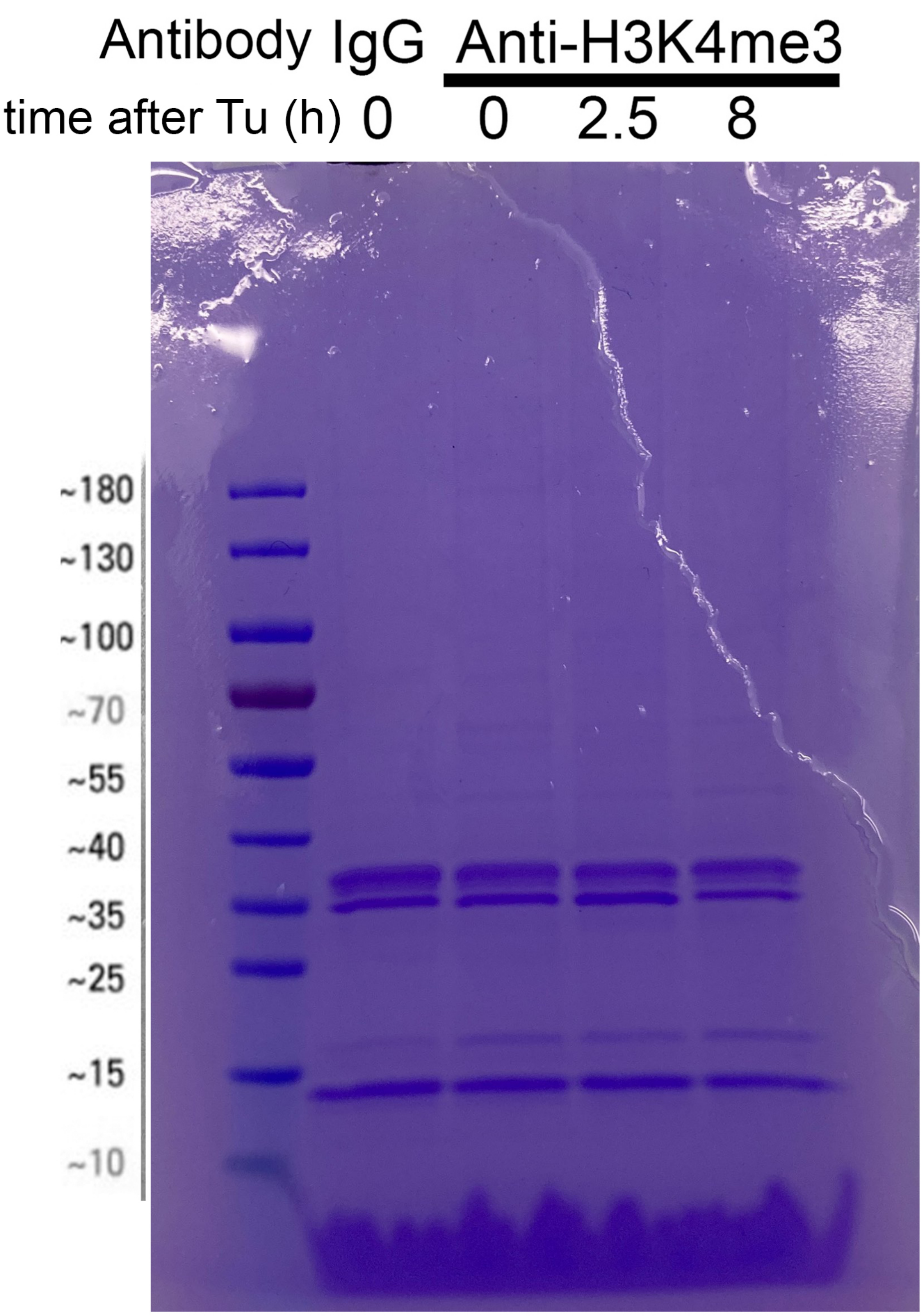

Fig. S4B

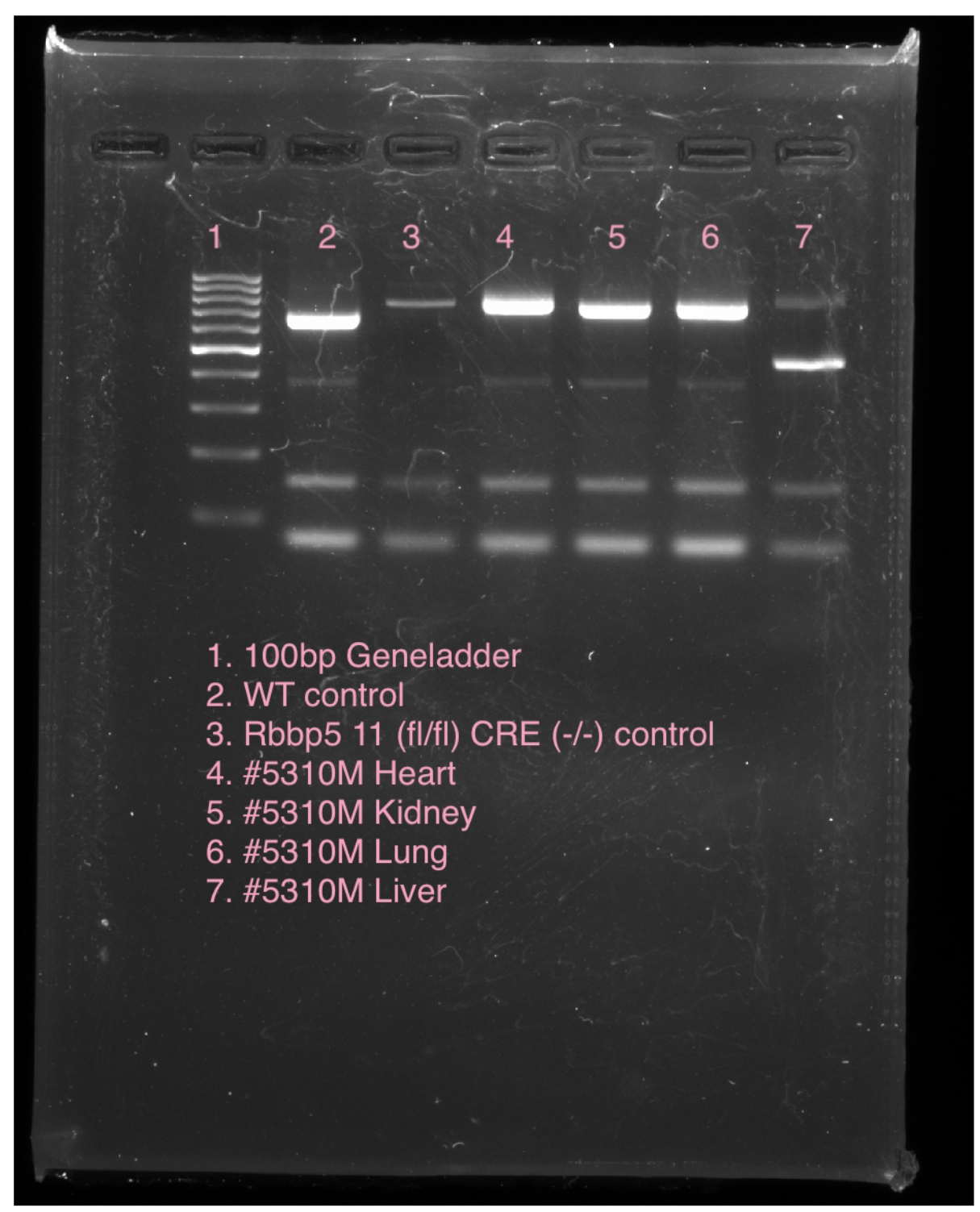

Fig. 3K

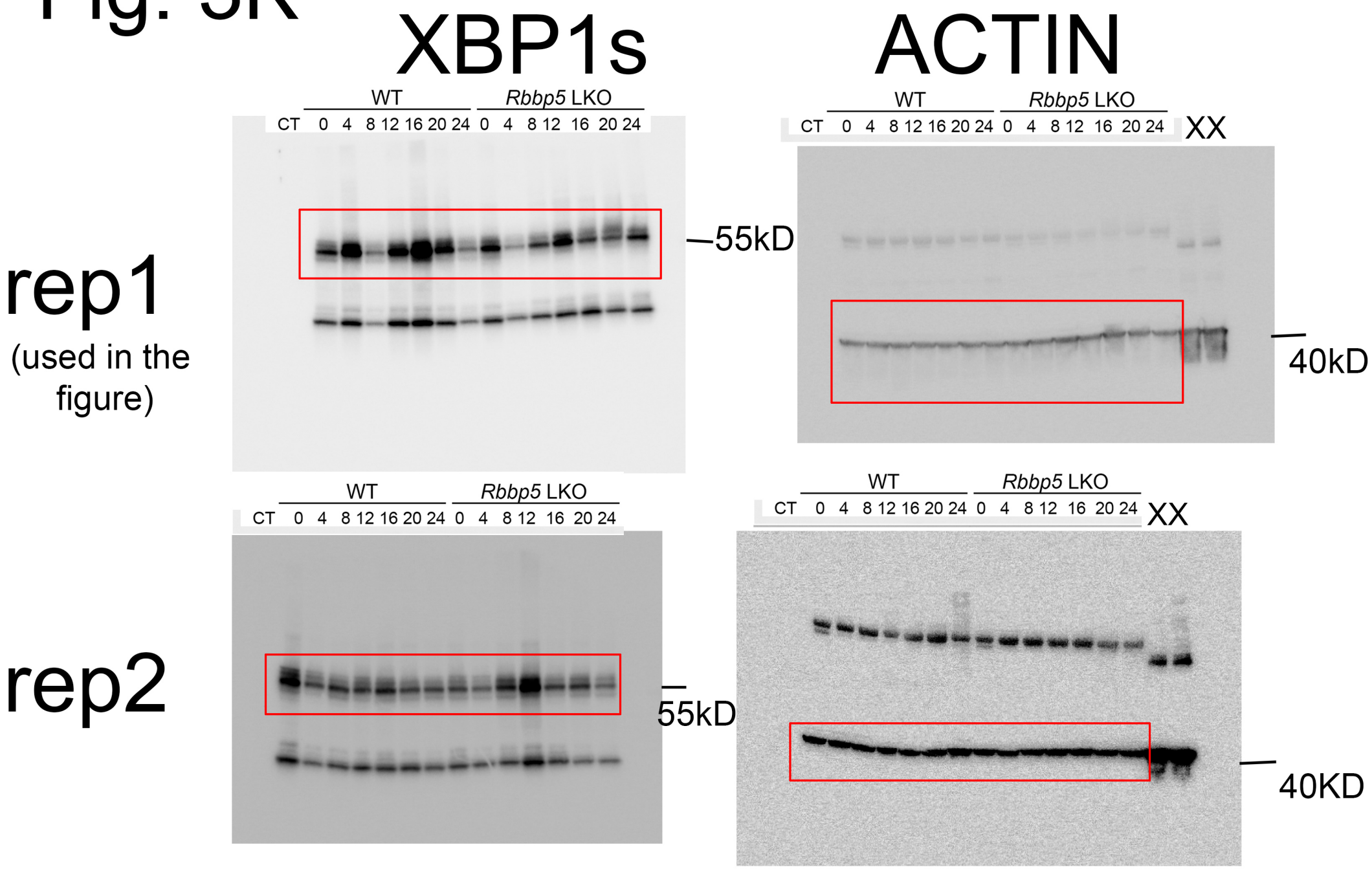
XBP1s
ACTIN
WT
Rbbp5 LKO
CT 0 4 8 12 16 20 24 0 4 8 12 16 20 24
XX
55kD
40kD
40KD
rep1
(used in the figure)
rep2
