# Supplementary material for: The histone methyltransferase complex subunit RBBP5 serves as a central epigenetic regulator of proteostasis dynamics
Source: PLoS Biol. 2026 Oct 8;24(10):e3004048. doi: 10.1371/journal.pbio.3004048 (PMC13667922; doi:10.1371/journal.pbio.3004048)
Supplement: Supplementary file 2 [file pbio.3004048.s032.pdf]

Reviewer #1: The data in this manuscript are really interesting and it should be published. Clear evidence is presented that RBBP5 is required for many transcriptional responses in cells and in vivo. However, the evidence causally connecting implicating RBBP5 as part of a putative ~12h ultradian clock is less than convincing and I'm concerned that the authors overlook other plausible interpretations in favour of their preferred hypothesis. I also have reservations about the way in which these findings are communicated:

1. The liver of a freely behaving mouse is not under constant conditions. In addition to any cell-autonomous regulatory processes, there will be daily and higher frequency fluctuations in feeding, body temperature and various hormonal cues. Therefore, there is no reason to believe that the abundance of any given mRNA or transcription at any given genomic locus would be constant over time. Given previous reports (e.g., Hughes et al, 2009; Cretenet et al, 2012), its clear that some of this variation has a ~12h periodicity. The function and mechanism of these ~12h oscillations is the focus of this paper.

I am afraid that I am not as confident as the authors that "many of these ~12h ultradian rhythms are established by an XBP1s-dependent 12h ultradian oscillator separate from ... the ~24h circadian clock ..." What they have shown is that ~12h oscillations are observed in the absence of Bmal1, but so also are many circadian rhythms, e.g., Lipton et al., 2015; O'Neill and Reddy, 2011. Moreover, Cretenet et al found that ~12h oscillations were severely disrupted in Cry1/2-deficient mice. Therefore, please refine this claim. I also find the phrase "12h oscillator" ambiguous, because it suggests self-sufficiency and established independence from any other known mechanism, whereas I do not believe that this is widely accepted to be the case (e.g. Emery & Gachon, 2025).

The clear communication of their work would also be improved immensely if the authors could be more explicit to distinguish what they have actually shown from their speculation and interpretation. In particular, it would be more accurate and balanced if they were to make clear it is their hypothesis that ~12h oscillations are driven by a dedicated ~12h oscillator, rather than an established fact. This is important because to do otherwise would discount the possibility that there might be other explanations for their observations.

The way to test the authors' assertion that 12h oscillations are driven by a XBP1s-dependent oscillator independent of the cell or circadian cycle, would be to clamp XBP1s activity at a constant level within the normal range of physiological activity and then assess whether any other 12h oscillations remain downstream. Knocking out genes does not test this hypothesis, and cannot rule out epistasis. This is not a criticism of their work, but of its reasonable interpretation. The authors might be entirely correct in their interpretation, but the evidence presented is not strong enough to rule out other interpretations so they simply need to tone it down and add caveats.

We thank the reviewer for this thoughtful and constructive comment. We fully agree that the liver of a freely behaving mouse is not under constant conditions and that multiple systemic and environmental factors—including feeding behavior, body temperature, and hormonal fluctuations—can contribute to temporal variation in gene expression. We also agree that prior studies (e.g., Hughes et al., 2009; Cretenet et al., 2012) have documented ~12h periodic components in such physiological contexts. Our goal in this study is to investigate the potential mechanisms and functional relevance underlying these ~12h oscillations.

In response to the reviewer's concern regarding overstatement, we have substantially revised the Introduction to provide a more balanced and nuanced view of the current understanding of ~12h rhythms. Specifically, we now explicitly outline three non–mutually exclusive models for their regulation:

"Although the mechanisms governing these ~12h rhythms remain an active area of investigation, three non-mutually exclusive models have been proposed [23, 27–29]: (i) non–cell-autonomous regulation driven by interactions between the circadian clock and environmental cues [24, 30]; (ii) emergence of 12h rhythms from antiphase circadian transcriptional programs [31]; and (iii) the existence of a dedicated, cell-autonomous 12h oscillator [22, 32–34]. Supporting the latter model, we and others have provided strong evidence that many ~12-hour rhythms are driven by the unfolded protein response (UPR) transcription factor XBP1s, independently of both the canonical BMAL1-dependent circadian clock and the cell cycle [18–22, 29, 35]."

We would like to clarify that our use of the term “independent” specifically refers to independence from the canonical BMAL1-dependent circadian clock, rather than excluding all possible 24-hour regulatory influences. We have explicitly added “BMAL1-dependent” throughout the revised manuscript to avoid overgeneralization and to more precisely reflect this distinction.

We have also moderated our language throughout the manuscript to clarify that the concept of a “cell-autonomous 12h oscillator” represents a working model or hypothesis rather than an established fact. Importantly, we would like to note that this model has gained recognition in the field and is discussed as a plausible mechanism in several recent independent review articles (PMID: 40475272; PMID: **41845938**; PMID: 40753007), none of which are authored by us. Notably, one of these reviews includes Dr. Frederick Gachon—a pioneer in the field and an early proponent of non–cell-autonomous mechanisms—highlighting that consideration of a cell-autonomous 12h oscillator is increasingly accepted as part of the conceptual framework for ultradian regulation. We agree, however, that the degree of acceptance remains an evolving matter, and we have revised the text to reflect this appropriately.

Regarding the reviewer’s suggestion of constitutively clamping XBP1s activity to test sufficiency, we agree that this would be an important and rigorous experiment. However, such an approach is technically challenging in practice. Moreover, it is not necessarily expected that constitutive expression of a core regulatory factor would abolish rhythmicity. For example, constitutive expression of BMAL1 does not eliminate circadian oscillations but only makes it more susceptible to noise (e.g., PMID: 35999195). Thus, interpretation of such experiments may not be straightforward. To further address these mechanistic questions, we are currently developing mathematical models of the proposed 12h regulatory network to better understand the relative contributions of different components, including XBP1s-dependent and -independent mechanisms. We look forward to sharing these results with the community in future work.

Overall, we have revised the manuscript to more clearly distinguish between experimental observations and interpretation, and to explicitly acknowledge alternative explanations and remaining uncertainties.

2. There is an implicit assumption that these 12h rhythms in transcription/transcript abundance are functional rather than consequences of something else. I am not sure that this has been clearly established. For example, to my mind it is quite plausible that these 12h oscillations are epiphenomena of homeostatic processes and do not "drive" anything, i.e., what is the evidence that the abundance of any 12h oscillating species (mRNA, protein, metabolite) becomes rate-limiting for the activity of some downstream process at its nadir. To provide an example, outside of fasting, physiological blood glucose levels exhibit both daily and ultradian rhythms but, because it is homeostatically-regulated, there is no reason to believe that the availability of extracellular glucose is ever rate-limiting for the vast majority of glucose-dependent processes.

We thank the reviewer for this important point. We agree that the functional significance of ~12h oscillations, and whether they are causal drivers versus epiphenomena of underlying homeostatic processes, remains an important question in the field.

To address this concern, we have incorporated recent evidence supporting a functional role of ~12h rhythms. In particular, a recent study combining multi-omics profiling with kinetic mathematical modeling (PMID: 41511549) demonstrated that a substantial fraction of hepatic metabolic processes exhibit dominant ~12h rhythmicity. Importantly, these rhythms are not limited to transcript abundance but extend to metabolic fluxes and pathway-level activities, suggesting functional integration rather than passive oscillation.

Accordingly, we have added the following text to the revised manuscript:

“Functionally, the physiological relevance of these rhythms is most extensively characterized in the mouse liver, where integrative multi-omics analyses coupled with kinetic mathematical modeling reveal that a substantial fraction of hepatic metabolic processes—particularly those governing lipid and carbohydrate metabolism—exhibit dominant ~12h rhythmicity rather than classical ~24h circadian oscillations [37]. These findings underscore a tight functional coupling between the 12h UPR program and core metabolic regulation.”

While we acknowledge that this does not fully exclude the possibility that some observed oscillations may arise as secondary consequences of homeostatic regulation, these systems-level data support the notion that ~12h rhythms are embedded within core physiological processes. We further acknowledge that further work will be

required to determine whether specific oscillatory components become rate-limiting for downstream processes under defined conditions.

3. It is not clear to me what is the unappreciated epigenetic timing mechanism that the authors believe they have identified (see abstract).

We removed this claim from the abstract.

4. An alternative explanation for the authors' observations is that RBBP5 is an essential or conditionally essential gene that regulates transcription globally and promiscuously as part of the KMT2 family of methyltransferase complexes. Interfering with RBBP5 function therefore globally disrupts transcription of genes that are regulated by H3K4me, which includes many genes encoding proteins that regulate protein homeostasis. Thus, what evidence argues against the possibility that loss of RBBP5 may simply be epistatic to many transcriptional responses and programs.

We think the strongest evidence arguing against this possibility is that RBBP5 itself exhibits a robust ~12h rhythm in both expressions (not just restricted to the liver, but also in many other mouse tissues, see response below) and chromatin binding. In our view, this evidence argues against RBBP5 acting merely as a constitutive, global, and promiscuous transcriptional cofactor whose loss nonspecifically disrupts many downstream programs. Rather, its activity appears to be temporally organized.

More broadly, we favor a temporal function–relationship model, in which genes and pathways involved in related biological processes tend to cluster in time and display similar temporal profiles. In this context, the fact that the ~12 h rhythm of RBBP5 is tightly synchronized with the ~12 h rhythm of UPR/proteostasis gene expression strongly suggests that RBBP5 is not simply a general epistatic regulator of transcription, but instead plays a more specific role in controlling proteostasis dynamics. This temporal concordance is difficult to reconcile with a purely global transcriptional effect and instead supports the idea that RBBP5 is functionally integrated into the 12 h proteostasis program.

Consistent with this principle, our previous work provides an additional example: nuclear speckle phase separation dynamics also follow a ~12 h rhythm that is in phase with UPR/proteostasis gene expression. Building on this temporal alignment, we and others demonstrated that nuclear speckles play a critical role in the transcriptional regulation of stress responses, including heat shock and ER stress (PMID: **34985945,** PMID**: 41794640,** PMID**: 40764481,** PMID: **39413186,** PMID: **33355299)**. Together, these observations reinforce the idea that temporally coordinated regulation—rather than nonspecific global transcriptional disruption—underlies the role of RBBP5 in proteostasis control.

We added this point to our discussion as follows;

While our findings support a model in which RBBP5 functions as a key epigenetic regulator of proteostasis, an alternative explanation for our observations would be that RBBP5, as a core component of COMPASS, acts as an essential and broadly acting transcriptional cofactor, such that its loss nonspecifically perturbs global transcription, including genes involved in proteostasis. However, several lines of evidence argue against this interpretation. Most notably, RBBP5 itself exhibits robust ~12h rhythms across multiple tissues, indicating that its activity is temporally regulated rather than constitutive. In this context, we favor a temporal function–relationship model, whereby genes and pathways involved in related biological processes cluster in time and share similar temporal profiles [98]. The tight synchronization between the ~12h rhythm of RBBP5 and that of UPR/proteostasis gene expression strongly supports a more specific, functionally integrated role for RBBP5 in orchestrating proteostasis dynamics, rather than a purely global and epistatic effect on transcription. Consistent with this principle, our previous work demonstrated that nuclear speckle phase separation dynamics also follow a ~12h rhythm in phase with UPR/proteostasis programs [18], and that nuclear speckles play key roles in regulating transcriptional responses to proteotoxic stress, including the heat shock and ER stress [99-102]. Together, these findings support a model in which temporally coordinated regulation underlies proteostasis control. Finally, we propose that the 12h oscillator serves as an effective “discovery tool” for uncovering previously unrecognized mechanisms of proteostasis regulation. Continued exploration of the 12h oscillator will likely reveal additional hidden principles governing proteostasis beyond nuclear speckles and RBBP5.

All figures - to save the reader having to look it up each time, it would be helpful to state in each relevant figure legend whether the mice were in LD or DD conditions. If LD, when was lights on (ZT0)?

We thank the reviewer for this helpful suggestion. We have now revised all relevant figure legends to explicitly indicate whether mice were maintained under LD or DD conditions, and for LD experiments we have specified the timing of lights on (ZT0) in each case to improve clarity and readability. As a general convention used throughout the manuscript, CT (circadian time) refers to mice housed under constant darkness (DD), whereas ZT (zeitgeber time) refers to mice maintained under a 12 h light/12 h dark (LD) cycle, with ZT0 corresponding to the time of lights on, and ZT12 the time of lights off.

Fig 1B. If the idea is that there is change over time then it seems to be missing an appropriate statistical test.

We performed statistical tests with cosinor2 package (PMID: **24725531)** as this method is specifically designed for the analysis of short and sparsely sampled time-series data. These results have been updated in Fig. 1B. with only H3K4me3 showing a statistically significant p value of 0.024.

Fig 1C. Is it a subjective judgement or is there some statistical analysis to support the claim of robust 12h rhythms please?

To increase the stringency of the RBBP5 ChIP-seq analysis, we performed a second independent temporal ChIP-seq experiment using separate biological samples. Figure 1E (the quantification of Fig. 1C and S2A) has been updated to reflect the averaged signal from both independent runs. Using RAIN, we identified a robust global ~12 h oscillation in RBBP5 chromatin binding ($P = 0.0016$).

Fig 1E. Presented as continuous data but isn't. Fine to show a trend line but please also show the data points so that the variance and temporal resolution is clear.

As suggested, we showed all data points as well as the trend line.

Fig S1H. It would be helpful to see a graph comparing the relative amplitudes of "rhythmic genes". This would allow the reader to assess the extent of biological variation, rather just how many significant rhythms there are.

The amplitude of RBBP5 chromatin binding rhythms is already conveyed in the heatmap and corresponding quantification (Fig. S2A), which together illustrate both the relative magnitude and variability of oscillatory signals across sites. We therefore believe that an additional graph specifically summarizing amplitudes would be redundant and would not substantially enhance the interpretation of the data.

Fig S1D. It is not sufficiently strong evidence to claim a ~12h rhythm in nuclear RBBP5. Please show replicates, whole cell RBBP5, appropriate loading control and appropriate statistical analysis.

We thank the reviewer for this important comment.

First, the quantification shown in Fig. S1D is derived from two independent biological replicates. Due to space limitations, only a representative blot from one experiment is presented in the main figure. To ensure full transparency, we have now included the raw blots from both biological replicates in Data S1.

Second, protein levels were normalized to total protein using Ponceau S staining rather than a single housekeeping protein. In our view, total protein normalization provides a more accurate and unbiased assessment of loading variability compared to individual loading controls, which can themselves exhibit variability under different physiological conditions. The corresponding raw Ponceau S staining images have also been included in Data S1.

Third, we performed statistical analyses of ~12 h rhythmicity using four independent methods—RAIN, MetaCycle, JTK_CYCLE, and cosinor2—and all methods consistently identified significant ~12 h oscillations ($p < 0.05$), supporting the robustness of this observation.

Finally, RBBP5 is predominantly localized to the nucleus, as demonstrated by our immunofluorescence data (Fig. 3B and Fig. 5B). This is further supported by data from the Human Protein Atlas, which shows almost exclusive nuclear localization of RBBP5 across multiple human cell types (**review figure 1A**). Thus, nuclear RBBP5 levels

closely reflect total cellular RBBP5. Consistent with this, analysis of a recently published mouse time-series proteomics dataset (PMID: 41519126) revealed robust ~12 h oscillations of total RBBP5 protein across multiple tissues—including liver, heart, iWAT, kidney, and several brain regions—with peak expression at CT10–14 and CT22–26 (Fig. S1E, F and **review figure 1B**).

Together, these additional data and analyses further strengthen the conclusion that RBBP5 exhibits robust ~12 h rhythmicity at the protein level.

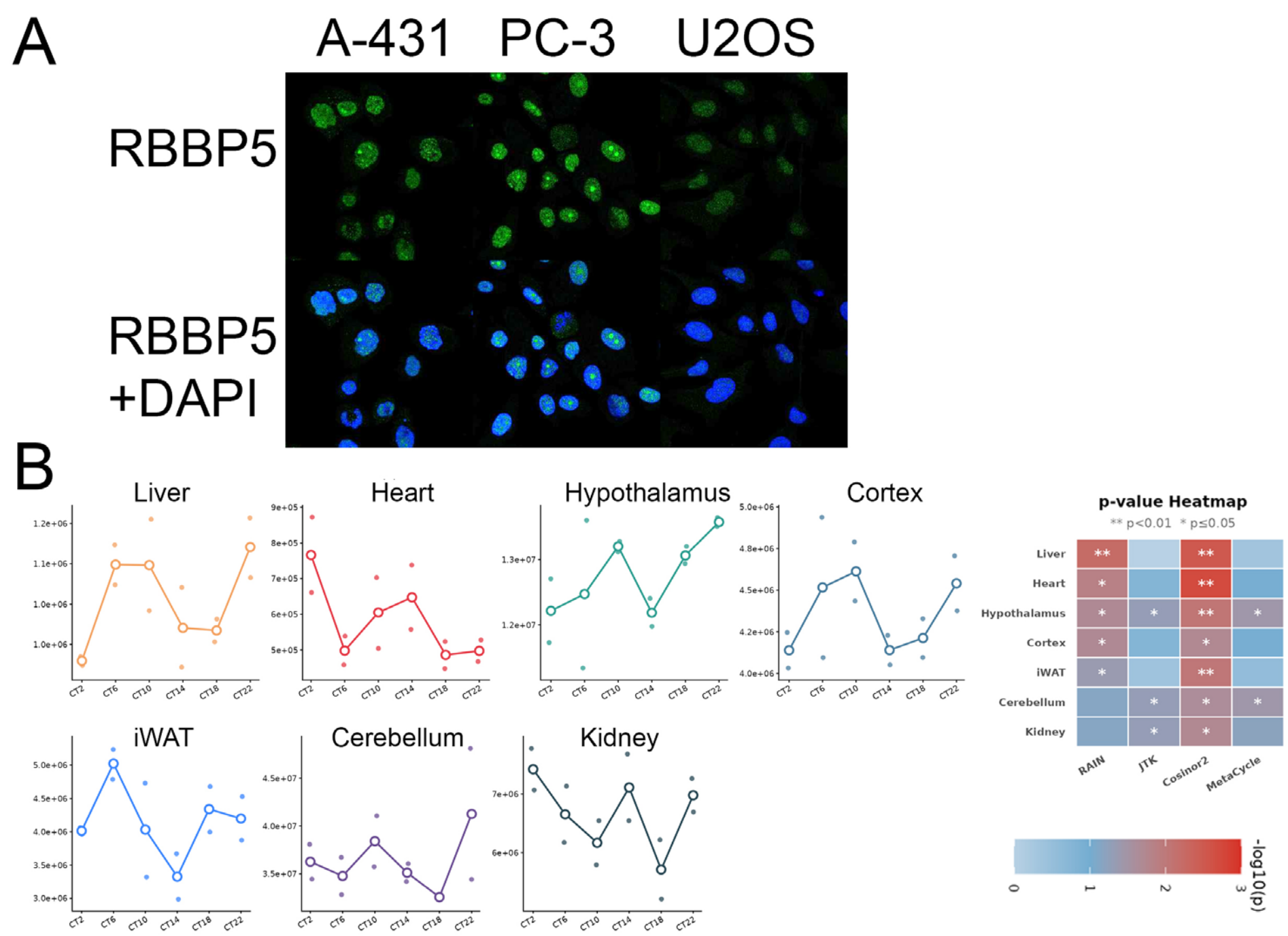

Review Figure 1. **(A)** IF of RBBP5 in three human cell lines, from human protein atlas (https://www.proteinatlas.org/ENSG00000117222-RBBP5/subcellular). **(B)** Temporal expression of total RBBP5 proteins in different mice tissues previously reported in PMID: 41519126. Heatmap demonstrates the P values for having 12h rhythm of RBBP5 expression in each tissue tested by four different methods.

Fig 2A. Not clear what a non-12h cycling gene is. Does this mean it cycles but not with 12h periodicity, or that it doesn't cycle. How was each gene's promoter defined please? Please consider whether the correlation might be explained by a third variable, e.g., RBBP5 binding correlates with expression level, expression level correlates with 12h cycling.

“Non-12 h cycling genes” refers to genes that lack ~12 h rhythmicity, but are not necessarily arrhythmic overall (i.e., they may exhibit other periodicities such as circadian rhythms). Promoter regions were defined as ±2 kb around the transcription start site (TSS), and RBBP5 peaks falling within this window were considered promoter binding events. Finally, we agree that the observed correlation may reflect an underlying variable: RBBP5 chromatin binding is most likely driven by its own expression, which displays robust ~12 h rhythms at both the mRNA and protein levels peaking at CT0, 12, 24 and 36, the same as its chromatin binding (Fig. S1C, D).

Fig 2B. All the RBBP5 ChiP-seq genes seem to be in the same phase, whereas nearly half of the RNA-seq genes look to be in antiphase. A phase scatterplot of Chip-seq vs RNA-Seq phase would be helpful here to test the authors' suggestion that the former is "driving" the latter. How well do they correlate. Similarly a relative amplitude scatterplot for Chip-seq vs RNA-Seq would provide additional insight.

We appreciate this suggestion. In response, we have added both a phase scatterplot and a bar chart (Fig. 2B) comparing the phase relationship between RBBP5 chromatin binding and gene expression. These analyses reveal a statistically significant phase difference, with RBBP5 binding consistently preceding gene expression. This temporal offset supports our interpretation that rhythmic RBBP5 occupancy is associated with, and may contribute to, subsequent transcriptional output.

Fig 2E. Some of these are overexposed. Good practise guidelines dictate the input should really be on the same blot as the IP, without cropping the bands. Where can the raw blots be assessed please?

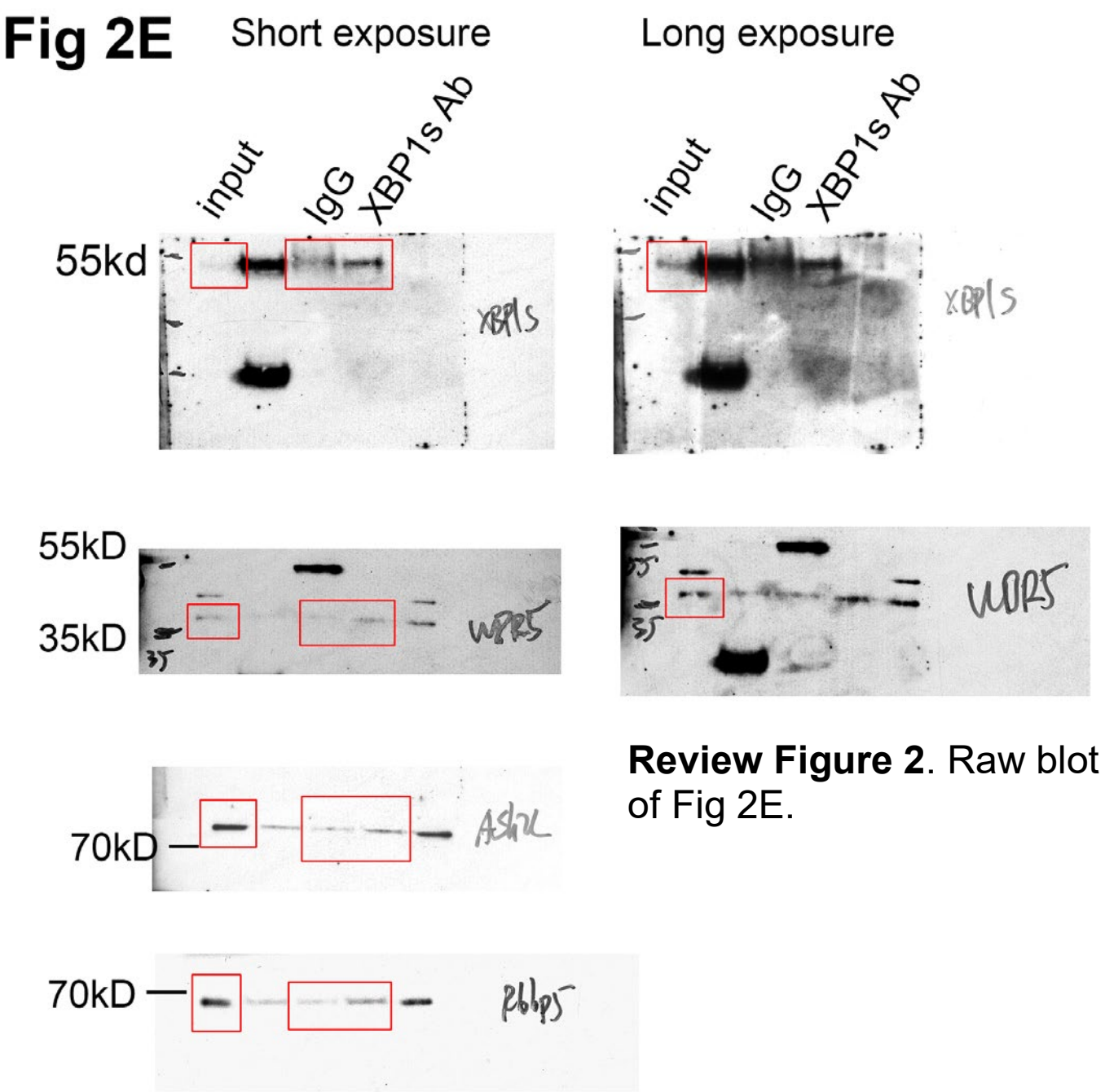

Review Figure 2. Raw blot of Fig 2E.

We have provided all raw blot images and included them as Data S1. Specifically for Fig. 2E, the corresponding raw blot has been added as a **Reviewer Figure 2**. The input samples were run on the same blot as the IP, although not positioned adjacent to the IP lanes. In addition, for XBP1s and WDR5, longer exposure times were required to clearly visualize the input signals, as the XBP1s pull-down was highly efficient. For this reason, the blot was cropped for presentation clarity.

Fig 3B. Fig S2C shows there is detected in the CRE =/- liver. This is difficult to reconcile with Fig 3B. Is the same antibody being used for the IF and western blot? If not, what region of the protein does each recognise. Is this really a partial LoF rather than full KO? Fig S2C. Where can the raw blots be assessed please?

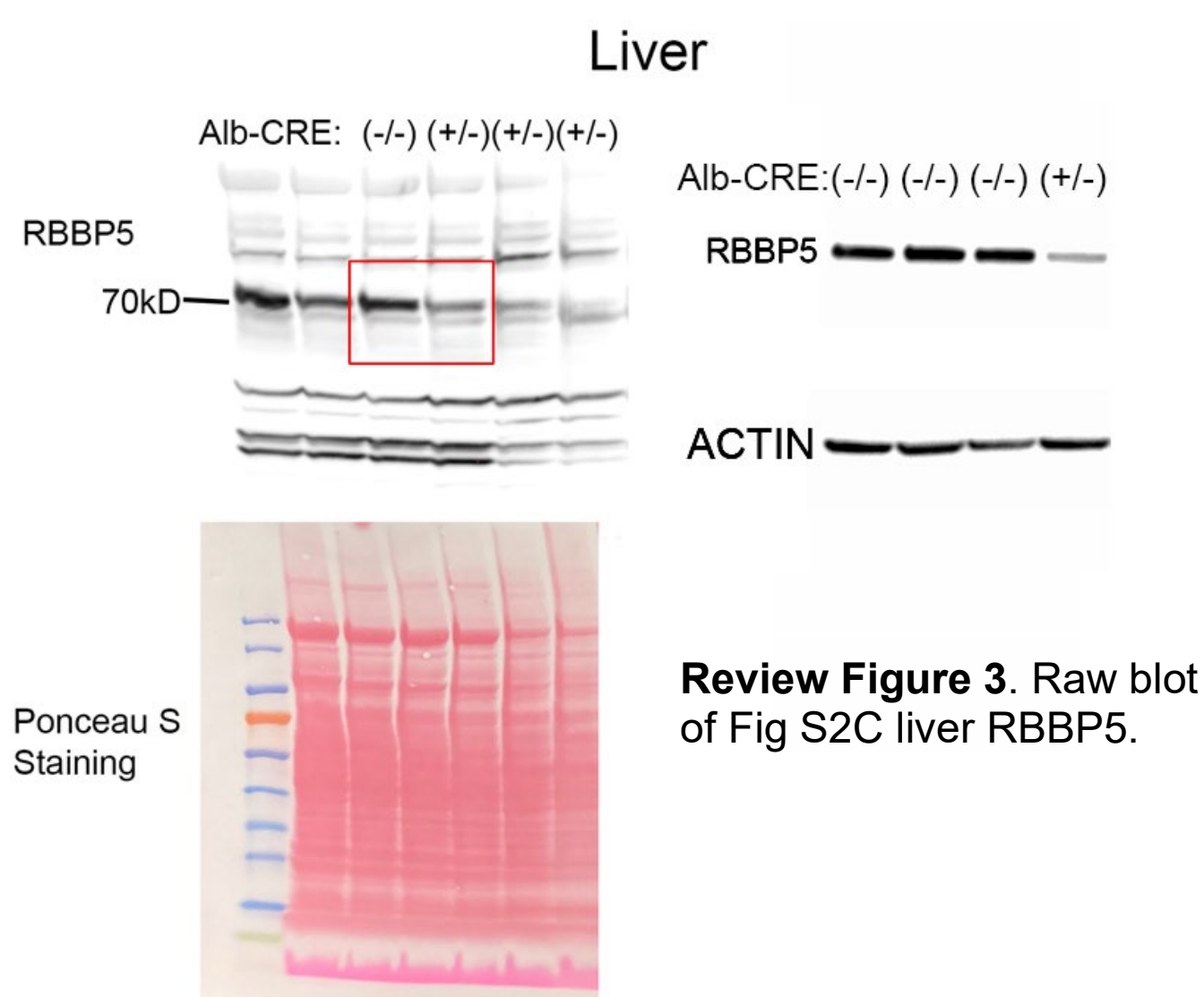

Review Figure 3. Raw blot of Fig S2C liver RBBP5.

We thank the reviewer for this important question. The same antibody was used for both immunofluorescence (IF) and western blot analyses. RBBP5 deletion was achieved using Alb-Cre, which drives recombination specifically in hepatocytes, where Albumin is highly expressed. As a result, RBBP5 is efficiently deleted in hepatocytes but remains present in non-parenchymal liver cell types, including Kupffer cells, liver sinusoidal endothelial cells, and hepatic stellate cells. Although hepatocytes constitute up to 80% of liver cells, the residual RBBP5 signal observed in Fig. S4C likely originates from these non-hepatocyte populations, consistent with the ubiquitous expression of RBBP5. Therefore, this model represents a hepatocyte-specific knockout rather than a global liver loss of RBBP5. In the IF analysis, the loss of RBBP5 staining is correspondingly restricted to hepatocytes of the liver. To improve clarity and precision, we have revised the terminology throughout the manuscript from “RBBP5 liver-specific knockout” to “RBBP5 liver hepatocyte-specific knockout.” We have also provided the raw western blot data, along with additional blots from independent experiments, which can be found in **Reviewer Fig 3.** In addition, for western blot analysis, we used small pieces of liver tissue rather than homogenizing the entire liver. Therefore, the varying levels of residual RBBP5 observed across different hepatocyte-specific RBBP5 knockout samples likely reflect differences in the relative contribution of non-hepatocyte cell types in each sampled piece.

Fig 3C. Doesn't instill confidence in the analytical approach by claiming there are thousands of genes with periodicities >30h. I've used RAIN, it identifies loads of false positives. The claim that it robustly uncovers ultradian rhythms is spurious. The reader would have more confidence if a higher level of stringency were applied to the rhythmicity analysis.

We appreciate the reviewer’s concern regarding the analytical approach and the potential for false positives when using RAIN. As described in several of our prior studies (e.g., *PLOS Biology* 2020 PMID: **31935211**; *Frontiers in Biology* 2023 PMID: **37324401**; *NPJ Biological Timing and Sleep* 2024 PMID: **39148626**), RAIN is particularly well-suited for detecting ultradian rhythms because it does not assume a symmetric (sinusoidal) waveform. This feature is critical, as many ultradian oscillations—including the 12 h rhythms studied here—are

inherently non-sinusoidal, in part because they are often superimposed on (and thus masked by) a larger circadian rhythm (Zhu et al, Cell metabolism 2017 PMID: **28591634;** van der Veen and Gerkema, FASEB J 2016 PMID: **27871062)**. We agree that periodicities longer than 24 h should be interpreted with caution. Due to the limited temporal window of sampling, these longer periods are supported by fewer than two full cycles and thus inherently have lower confidence compared to shorter oscillations (e.g., 12 h), where multiple cycles are observed.

Importantly, to address this concern and further strengthen the robustness of our conclusions, we have incorporated additional analytical approaches in the revision. Specifically, we applied both MetaCycle and cosinor-based methods, which rely on different statistical frameworks and assumptions. Despite their lower sensitivity for non-sinusoidal ultradian rhythms, both methods independently recapitulate our central finding—namely, a near-complete loss of 12 h rhythmic gene expression in Rbbp5 LKO mice. These new analyses are now included in a new Fig. S7. Furthermore, in our original analysis, we also employed the eigenvalue/pencil method, which is fundamentally distinct from statistical rhythm detection approaches. This method performs spectral decomposition without requiring pre-specified period ranges and enables unbiased identification of superimposed oscillations.

Taken together, the convergence of results from four independent methods—RAIN, MetaCycle, cosinor, and eigenvalue/pencil—provides strong support for the robustness of our conclusions. We therefore believe that our analytical framework is both rigorous and appropriate for capturing ultradian rhythmicity.

Fig 3G. What is a superimposed ~12h rhythm please?

By “superimposed ~12 h rhythm,” we refer to the observation that many ultradian rhythms (particularly ~8 h and ~12 h) are often overlaid with circadian (~24 h) oscillations within the same signal (the same gene) (**PMID: 27871062; PMID: 28591634; PMID: 30231032; PMID: 30472532)**. As a result, the composite waveform reflects the combination of multiple periodic components, making the underlying ~12 h rhythm difficult to detect using traditional rhythm-identification tools such as JTK_CYCLE or MetaCycle, which typically assume a single dominant periodicity. To address this, signal-processing approaches (i.e., spectral decomposition methods) are required to separate (“decompose”) the composite signal into its constituent oscillatory components. In our study, we used the eigenvalue/pencil method to achieve this decomposition. Thus, “superimposed ~12 h rhythm” refers to the underlying ~12 h oscillatory component that becomes evident after removing (filtering out) the circadian and other oscillatory components from the original signal.

Fig S6A. Error bars too faint for me to see. What is the null hypothesis please? The three traces look very similar to my eyes. What is the p-value for fitting a straight-line vs a 12h damped sine wave?

We have replaced Fig. S9A (old S6A) with an updated version featuring clearer and more visible error bars. To assess rhythmicity, we used the RAIN algorithm. In this analysis, the null hypothesis is that the time-series data consist of independent, randomly ordered observations with no underlying rhythmic pattern. The alternative hypothesis is that the data exhibits a rhythm with a predefined period (in this case, 12 hours). Rhythmicity is considered significant when the p-value is less than 0.05, at which point the null hypothesis is rejected in favor of the alternative. We chose RAIN because of its ability to robustly detect both sinusoidal and non-sinusoidal rhythms, which is particularly important given that many ultradian rhythms deviate from a simple sine-wave pattern.

Fig S6B. Please check x-axis labelling. It would be helpful to see the raw data before detrending and some more examples.

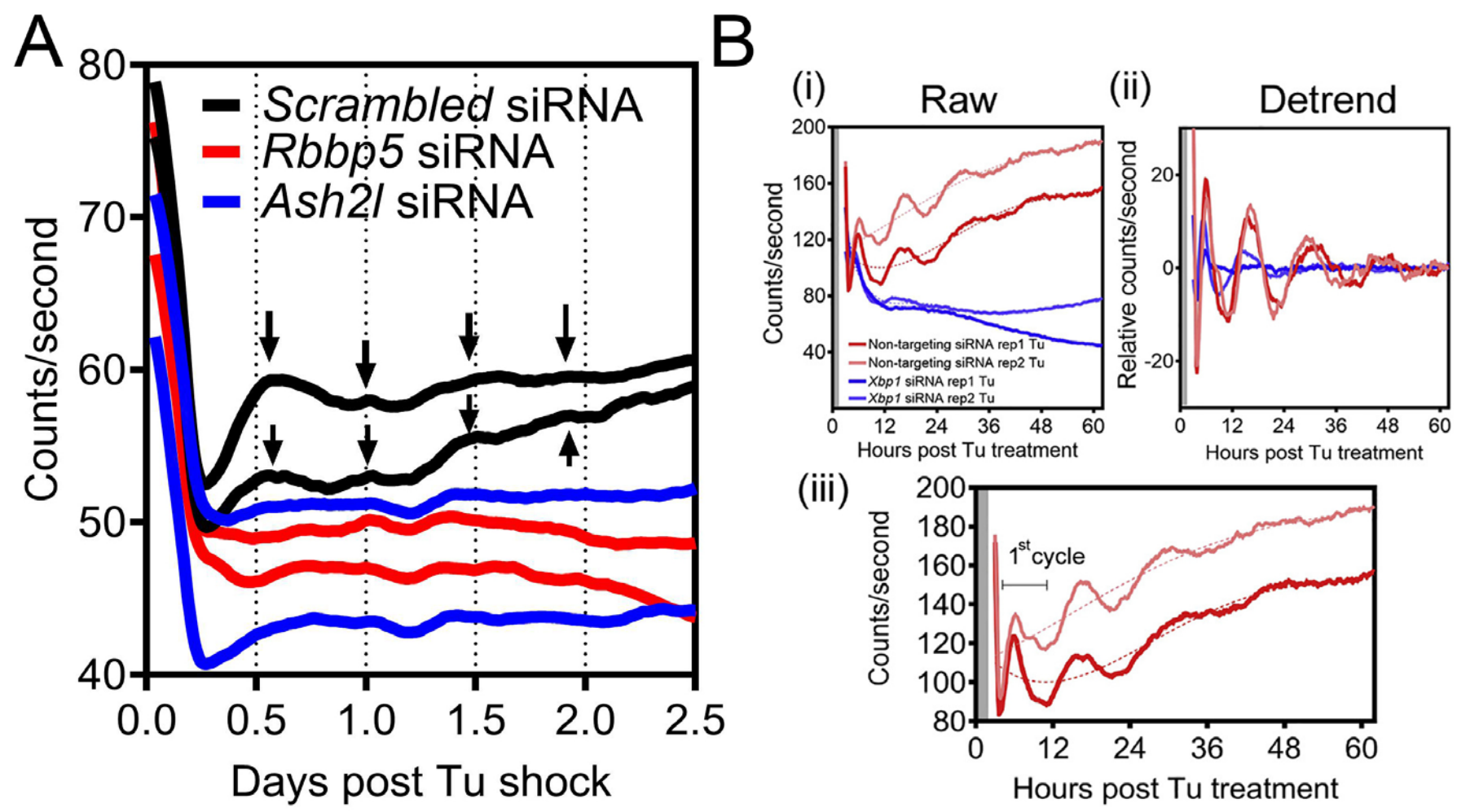

Review Figure 4. **(A)** Raw luciferase trace of *Manf* promoter-driven dluc activity in MEFs after Tu treatment. **(B)** Data from Fig. 3F of PMID: 28591634.

We have revised the x-axis label to "days post Tu treatment." In addition, as requested, we have included the raw (undetrended) data in the new Fig. S9B. While we acknowledge that the amplitude of the Manf-dLuc oscillation is modest, the overall rhythmic pattern is consistent with previously reported luciferase assays using Perk-dLuc MEFs following Tu synchronization (Fig. 3F, G; PMID: 28591634).

Furthermore, we have included additional examples of raw data, which are now presented in the **Reviewer Figure 4**, alongside the original Fig. 3F from Zhu et al., 2017 (*Cell Metabolism:* PMID: 28591634) for direct comparison.

Fig 4F. I don't understand how RBBP5 binding can be measured in the KO unless it is an incomplete KO.

We apologize for the confusion in the figure legend. Fig. 4F shows the fold induction of genes in response to Tu in WT and RBBP5 LKO mice, stratified based on whether their promoters are bound by RBBP5 in WT mice. Thus, the legend "Without RBBP5 binding in Rbbp5 LKO" refers to the log2 fold induction by Tu in Rbbp5 LKO mouse liver for genes whose promoters are not bound by RBBP5 in WT mice. We have revised the figure legend accordingly.

Fig 4. Where can these blots be assessed please? Why is the ponceau statin black and white in G and coloured in H. A more traditional loading control might be better. Is there quantification for these blots please?

For all Western blots, we quantified the protein band intensities and provided the corresponding raw blots, including biological repeats, in Data S1. ACTIN as the loading control was added for Fig. 4G. For Fig. 4B and 4H, ACTIN was not run for the original experiment unfortunately; instead, total protein staining by Ponceau S was used as the loading control. All Ponceau S staining images are also provided in Data S1. All changes were updated in the new figure 4.

Line 260. "Before the discovery of the mammalian 12h oscillator, the dynamics of proteostasis were primarily studied as transient responses to various proteotoxic stresses, with a classic example being the UPR triggered by ER stress." ....without wanting to in any way diminish the authors' work, I do not believe their research has been responsible for any paradigm shift in the wider proteostasis field. I could be wrong of course. I already expressed by skepticism about whether it is widely accepted that a mammalian 12h oscillator has been discovered, but it is certainly an interesting hypothesis.

We thank the reviewer for this comment.

First, we would like to clarify that this sentence is intended as a transition from our discussion of 12-hour rhythmic UPR dynamics to the canonical, stress-induced UPR. It is not meant to imply that our work represents a paradigm shift in the broader proteostasis field. We agree that such a claim would be overstated.

That said, our work does highlight a previously underappreciated aspect of UPR biology—namely, that in addition to its well-established role as a transient response to ER stress, UPR activity can also exhibit self-sustained ~12-hour oscillatory dynamics. This observation raises important questions about how distinct temporal modes of UPR dynamics may encode different biological information and influence cell fate, a topic we are actively pursuing. In support of the relevance of this concept, our work has been cited by leaders in the UPR field, including Peter Walter (PMID: 39501044; see Fig. 1).

To address the reviewer's concern and improve clarity, we have revised the beginning of the paragraph to: "In contrast to the self-sustained oscillatory dynamics of the UPR, its regulation has been more extensively characterized as a transient response to proteotoxic stress, most notably during ER stress."

Finally, regarding the broader acceptance of a mammalian 12-hour oscillator, we have addressed this point in detail in our previous responses and do not further elaborate here.

Line 312. " RBBP5 is both necessary and sufficient for the transcriptional regulation of hepatic response to acute proteotoxic stress in vivo". I cannot imagine the authors are really suggested that RBBP5 alone is sufficient for this. The rest of the complex and downstream mechanisms are obviously going to be necessary also. Please rephrase and think about there is any stronger evidence that might support this claim - the data presented suggests that some transcriptional responses to tunicamycin are modulated by the abundance of RBBP5 .... however the over-expression experiment is difficult to understand because I can't see any strong evidence that RBBP5 is over-expressed in 4H - please show all the blots with quantification. If RBBP5 were over-expressed, how would this lead to increased COMPASS activity without commensurate expression of the other complex subunits?

Thank you for these comments. First, we have now provided quantification of RBBP5 overexpression following tail-vein injection of Ad-TBG-RBBP5, showing an approximately 1.3-fold increase compared to control ($p = 0.003$). The corresponding quantification and raw blots have been included in the revised figure and data S1, respectively.

Second, we agree that our original statement that "RBBP5 is both necessary and sufficient" was inaccurate and overstated. We have revised the text to: "Taken together, these results demonstrate that RBBP5 modulates the transcriptional regulation of the hepatic response to acute proteotoxic stress in vivo."

Regarding the mechanism by which increased RBBP5 enhances the UPR, we propose that elevated RBBP5 levels may further facilitate the recruitment and assembly of the COMPASS complex at gene promoters, as RBBP5 (together with ASH2L) forms an essential heterodimer required for COMPASS assembly. Alternatively, increased RBBP5 may promote condensate formation of COMPASS at target promoters, as RBBP5 contains an intrinsically disordered region capable of phase separation in cell-free systems (data not shown). However, these possibilities remain speculative and are beyond the scope of the current study, and will require further investigation.

Fig 5A. Which are the MEF and which are the liver data?

The top UCSC genome browser track is derived from mouse liver, whereas the lower panel is from MEFs. We have revised the figure labeling to clarify this distinction.

Discussion - if the authors are receptive to my suggestions I expect that the discussion will change somewhat so I won't provide any feedback on it.

We revised our discussion accordingly.

Reviewer #2: Major comments:

The paper by Kubra et al is an exhaustive manuscript investigating the role of RBBP5 protein in ultradian rhythms in the liver. The experiments are well done and there are many striking findings within the manuscript, but in some cases they lack depth - and the claims made at the end are not sufficiently backed up.

Point 1: As they say, in line 472 of the discussion - "the epigenome of the mammalian ~12h ultradian oscillator remains completely unexplored" - yet they perform a 4h resolution ChIP-seq over 48 hours for RBBP5 - a typical circadian experiment rather than performing a 2hr resolution over 24h, which would be more appropriate for determining rhythms from an ultradian oscillator.

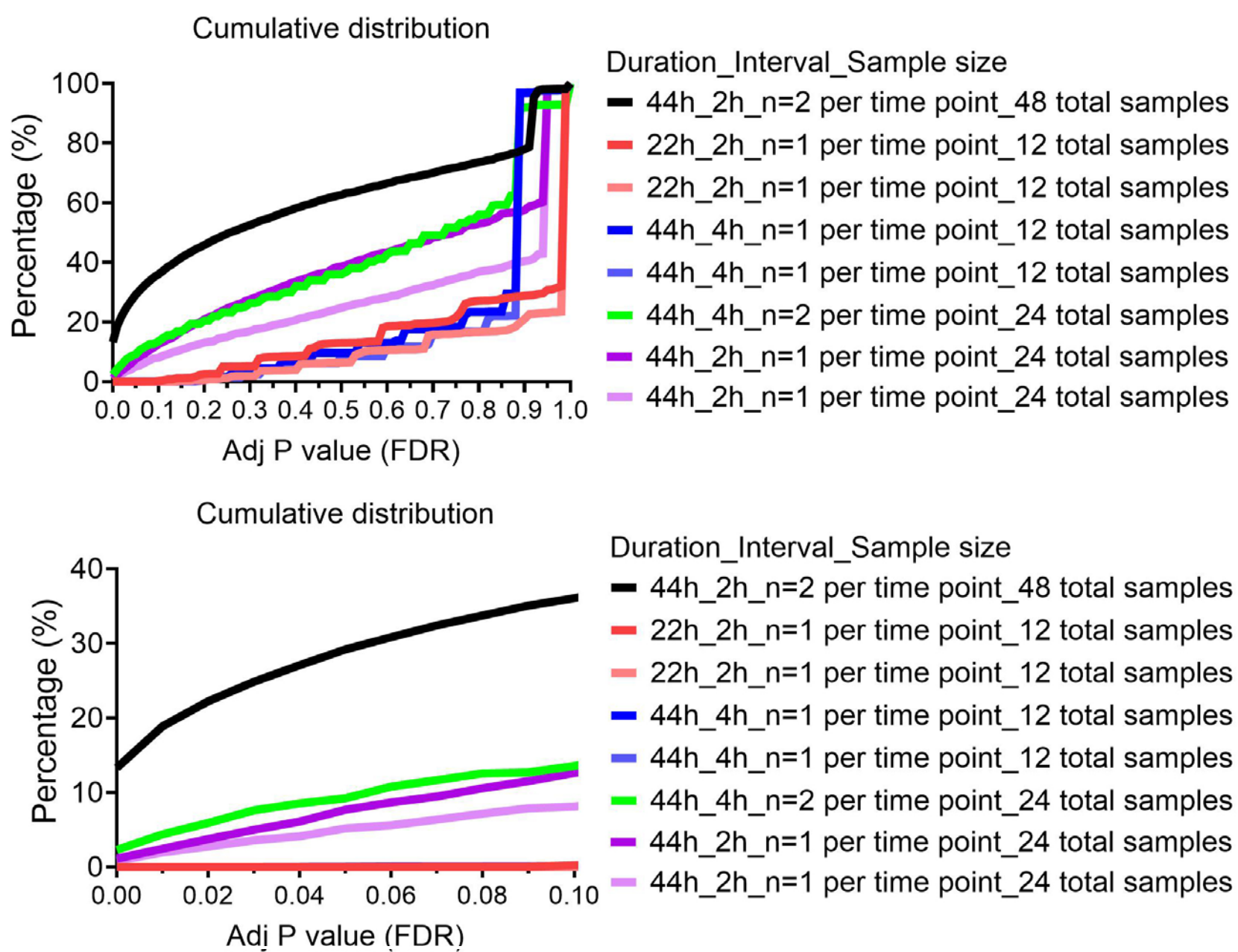

Review Figure 5. **Cumulative distributions of adj P value of having 12h rhythm with different sampling strategy.**

We appreciate the reviewer's point regarding the importance of sampling frequency and duration for accurately detecting ~12h ultradian rhythms. We agree that, in principle, higher temporal resolution can improve the detection of such oscillations.

To guide our experimental design in a cost-effective and statistically rigorous manner, we systematically evaluated this issue using previously published temporal RNA-seq datasets (2h resolution for 48 hours with duplicates at each time point). Specifically, we subsampled these data to compare different strategies, including 2 h versus 4 h sampling intervals, 24h versus 48 hour duration, as well as the inclusion or absence of biological replicates at each time point (n = 1 vs n = 2). We then assessed performance by examining the cumulative distribution of adjusted P values for ~12h rhythm detection. These analyses indicated that 2 h sampling over 24 h does not provide a substantial advantage over 4 h sampling over 48 h in terms of robustness for detecting ~12 h rhythms. Instead, increasing statistical power through biological replication (n = 2 per time point) had a markedly greater impact (**Review Fig. 5**).

Given these considerations, and the practical constraints of time and cost, we did not repeat the entire RBBP5 ChIP-seq at 2 h resolution for 48 hours with biological duplicates at each time point (the ideal scenario). Instead, we performed a second independent temporal ChIP-seq experiment at 4 h resolution over 48 hours and combined it with the original dataset. This approach substantially improved statistical robustness, enabling the identification of 4,667 ~12 h cycling RBBP5 binding events (adjusted P < 0.05) and 5,366 events (P < 0.05), compared to only 37 (adjusted P < 0.05) and 1,865 (P < 0.05) sites in the initial analysis, respectively. The updated results are presented in Fig. 2, Figs. S2 and 3 and Table S1 after incorporating both independent runs.

Point 2: In Line 419-422 of the discussion, the authors state that the "epigenome of the 12h oscillator is marked by COMPASS-mediated histone methylation"- and that "loss of one of the essential subunits of COMPASS, RBBP5, abolished more than 90% of the hepatic ~12h transcriptome".

For this statement to be valid, the definitive experiment - a H3K4Me3 ChIPSeq in RBBP5 LKO mice (with a 2hr resolution) - is missing. Would the ultradian genes specifically lose K4Me3 rhythms? "while having no effects on the core circadian clock"

We agree with the reviewer and have toned down the claim in the discussion as follows:

In this study, we uncovered that unlike the circadian clock, which was predominantly regulated by histone acetyltransferase (HAT) [87-89] and histone deacetylase (HDAC) [85, 90, 91], the epigenome of the 12h oscillator is associated with 12h H3K4me3 oscillation at the proximal promoters of UPR genes.

Point 3: In Fig2e, the authors show the XBP1s co-IP with RBBP5. Does it dynamically assemble/disassemble over 12hrs? What is the state of this complex in RBBP5 LKO mice? Does Xbp1 fail to target genes with a 12h rhythm in the RBBP5 LKO mice? What about the other components identified by proximity ligation? Are any of them specific to the epigenome of the ultradian oscillator? The final dataset of remodelers almost seems like a throwaway experiment - which is unfortunate.

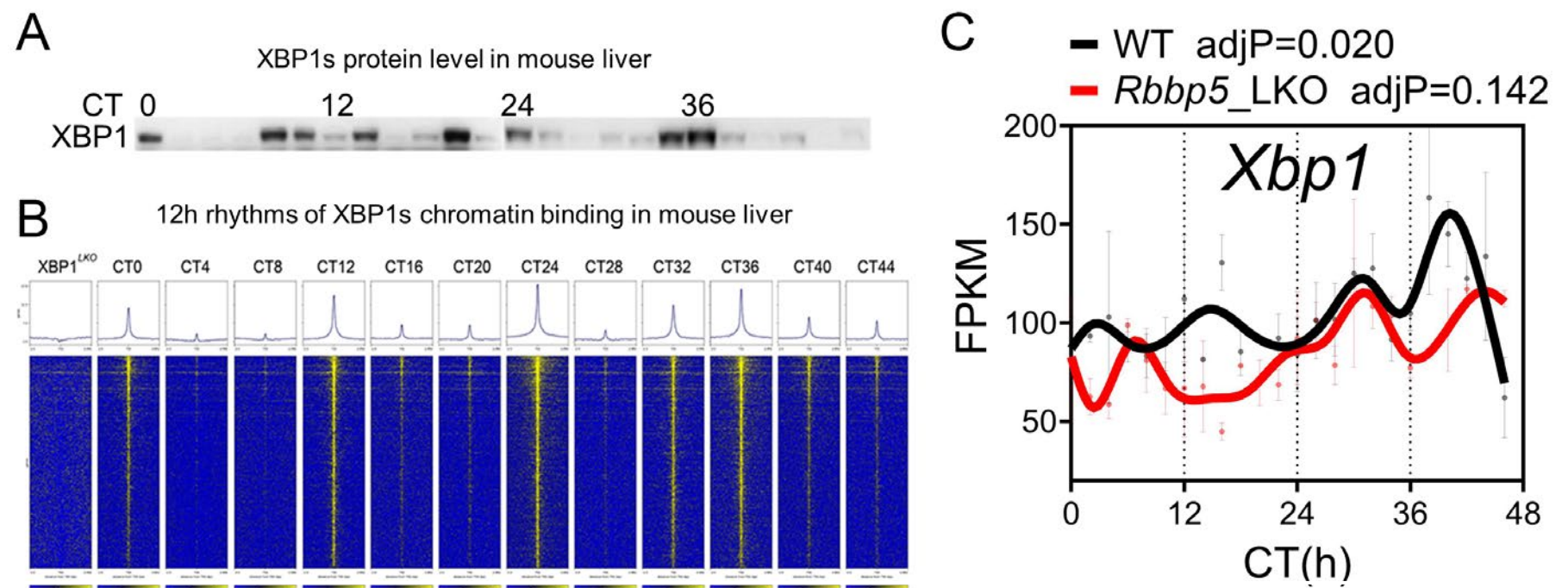

Review Figure 6. **(A)** Protein level of XBP1s in mouse liver as reported in PMID: **20074527, (B)** Heatmap showing XBP1s ChIP-seq signal in mouse liver, as reported in PMID: **31935211. (C)** Expression of Xbp1 mRNA assayed by RNA-seq in the mouse liver of WT and RBBP5_LKO mice. adj P value were calculated by RAIN to test for the existence of 12h rhythms.

We thank the reviewer for these insightful questions, which touch on two important aspects of our study: (1) the dynamics of XBP1s–RBBP5 interaction, and (2) the functional relevance of additional chromatin-associated components identified by proximity labeling.

1. Dynamics of XBP1s–RBBP5 interaction and effects of RBBP5 loss

The reviewer asks whether the interaction between XBP1s and RBBP5 dynamically assembles and disassembles with a 12-hour periodicity, and how this interaction behaves in RBBP5 LKO mice.

While this is an important question, previous data by us and others indicate that XBP1s itself exhibits highly robust 12-hour rhythms at both the protein level and in chromatin occupancy (**Review Figure 6A-B**). Notably, XBP1s abundance and binding are reduced to near-undetectable levels during trough phases (CT4–CT8 and CT16–CT20) (**Review Figure 6A**). This suggests that the dominant regulatory layer of XBP1s activity lies in its rhythmic expression and availability, rather than dynamic assembly/disassembly with cofactors such as RBBP5.

Because of this strong oscillation in XBP1s abundance, experimentally assessing interaction dynamics between XBP1s and RBBP5 across the 12-hour cycle would be inherently confounded by fluctuations in XBP1s protein levels. Similarly, in RBBP5 LKO mice, XBP1s expression is reduced and its rhythmicity is disrupted (**Review Figure 6C**), making it difficult to disentangle (i) direct effects of RBBP5 on XBP1s chromatin recruitment from (ii) indirect effects mediated through altered XBP1s expression and oscillatory dynamics.

For these reasons, we believe that experiments aimed at measuring interaction dynamics in this context would be difficult to interpret unambiguously. Instead, our current data supports a model in which RBBP5 functions within the chromatin landscape that assists XBP1s-dependent rhythmic transcription of target genes, rather than regulating XBP1s activity through cyclical complex assembly.

2. Relevance of additional chromatin components identified by proximity labeling

We agree with the reviewer that the chromatin remodelers identified in our proximity labeling dataset warrant further clarification regarding their relevance to the 12-hour oscillator.

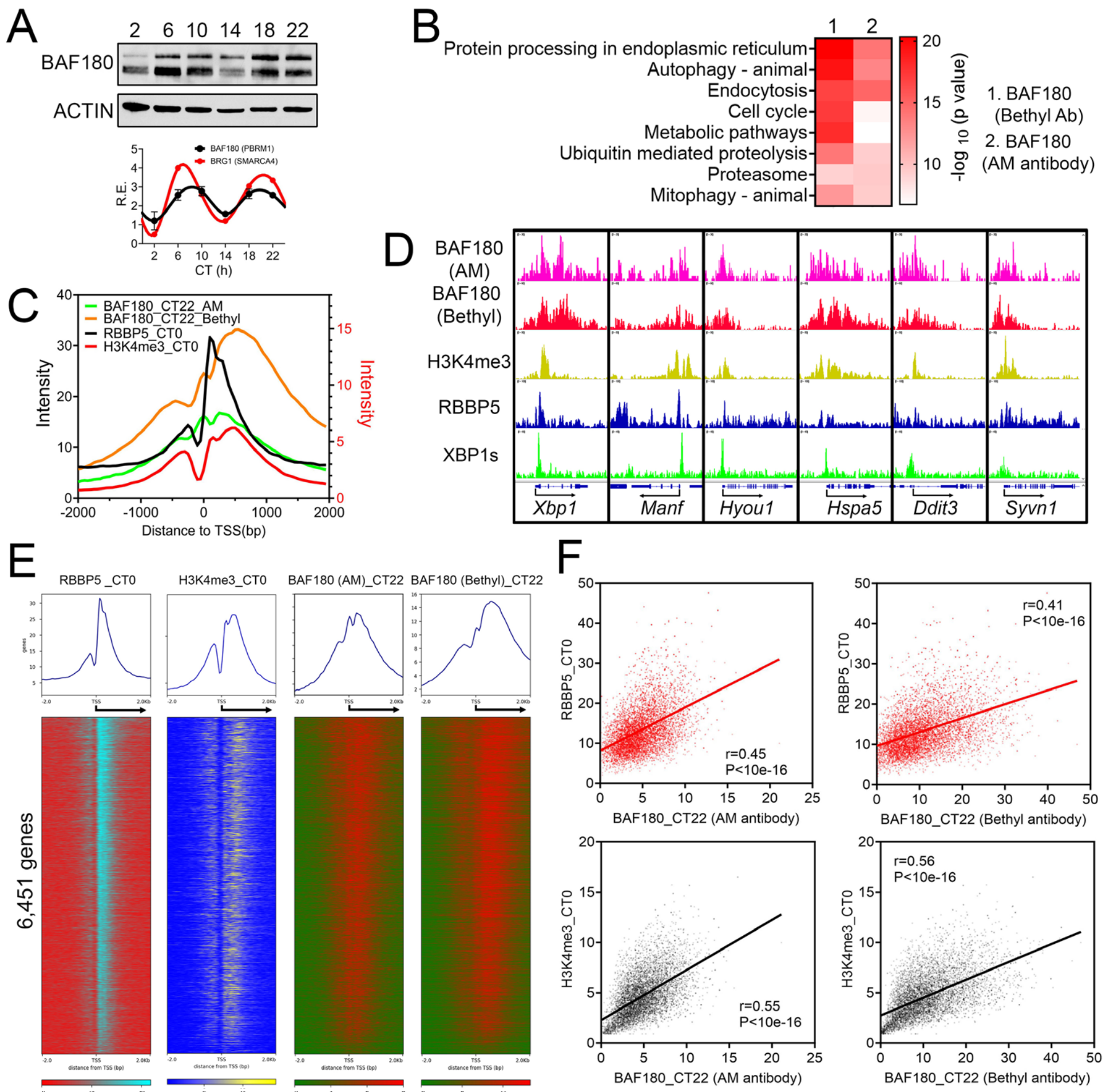

Review Figure 7. **(A)** Western blot and quantification of nuclear BAF180 in the liver of wild-type mouse liver at different CTs. The quantification of BRG1 is based upon the original blot reported in Fig. 5E in PMID: **26611104**. **(B)** GO analysis of BAF180 hepatic cistrome. **(C)** Quantification of RBBP5, H3K4me3 and BAF180 chromatin occupancy 2kb ± of TSS of 6,451 genes. **(D)** Selected UPR genes aligned for BAF180, RBBP5, H3K4me3 and XBP1s ChIP-seq signal in mouse liver. **(E)** Heatmap showing RBBP5, H3K4me3 and BAF180 chromatin occupancy 2kb ± of TSS of 6,451 genes. **(F)** Scatter plot comparing BAF180 binding signal with those of RBBP5 and H3K4me3 2kb downstream of TSS of 6,451 genes.Data: Mean ± S.E.M.

To address this, we performed additional analyses focusing on SWI/SNF (BAF/PBAF) components. Interestingly, even prior to our identification of the hepatic 12-hour oscillator, we had reported ~12-hour rhythmic expression of several SWI/SNF subunits, including PBRM1 (BAF180), ARID2 (BAF200), and SMARCA4 (BRG1), in mouse liver (PMID: **26611104)**. We have now extended these observations by: 1) experimentally validating the 12-hour rhythmicity of nuclear BAF180 protein peaks at CT10 and CT22 (**Review Figure 7A**), and 2) reanalyzing previously published BAF180 ChIP-seq datasets.

This analysis revealed that promoter-proximal BAF180 binding is significantly enriched at genes involved in proteostasis pathways (ER stress, autophagy, proteolysis) (**Review Figure 7B**). Moreover, we observe strong co-enrichment and positive correlation between BAF180, RBBP5, and H3K4me3 at these loci (**Review Figure 7C-F**), suggesting coordinated action of COMPASS and SWI/SNF complexes in regulating proteostasis genes. These findings support the idea that multiple chromatin regulators identified in our proximity labeling dataset are not incidental but instead may represent components of a broader epigenomic framework underlying 12-hour rhythmic transcription.

However, we acknowledge that these analyses remain correlative, and that direct functional interrogation (e.g., genetic perturbation of SWI/SNF components) will be required to establish causality and oscillator specificity. As such, we consider these results to be preliminary and beyond the scope of the current manuscript. To maintain focus and rigor, we have elected not to include these data in the main manuscript. For transparency, these analyses have been included in the revised bioRxiv preprint (https://www.biorxiv.org/content/10.1101/2024.09.13.612812v2.full) and are provided to reviewers as supplementary information.

Minor Point 4: A global criticism of the figures are the font sizes for some of the text within panels are just too small and impossible to read - ; TSS labels in 1C; gene names in Fig4d, X axis labels in Fig5a among others-

We thank the reviewer for this helpful comment. We have carefully revised all figures to improve readability by increasing font sizes across panels, including TSS labels in Fig. 1C, gene names in Fig. 4D, and axis labels in Fig. 5A, among others. We have made every effort to enlarge text elements to the maximum extent possible while maintaining overall figure layout and clarity.

Minor point 5: In Fig2, it would be important to plot and visualize RBBP5 ChIP-Seq on the 3926 non-12h cycling gene set to show that their patterns differ from the heat map for the 12h cycling gene set of 2525 genes shown in panel 2B.

We added two panels in Fig. S3A, B to demonstrate that RBBP5 chromatin binding is stronger and exhibits more robust ~12h rhythmicity at sites associated with 12h cycling genes compared to sites linked to genes lacking ~12h rhythmicity (**Fig. S3A, B**).

Minor errors: Fig S6B- axis label needs correction for time

We changed the label to “days post Tu treatment”. Thank you for catching that!
