# Supplementary material for: The histone methyltransferase complex subunit RBBP5 serves as a central epigenetic regulator of proteostasis dynamics
Source: PLoS Biol. 2026 Oct 8;24(10):e3004048. doi: 10.1371/journal.pbio.3004048 (PMC13667922; doi:10.1371/journal.pbio.3004048)
Supplement: Supplementary file 3 [file pbio.3004048.s033.docx]

Reviewer #2: I thank the authors for addressing my points but my primary concerns remain.

Point 1: regarding the argument against a higher- time resolution chip for RBBP5.

I can't understand why the authors preferred a 4hr window for a new set of duplicate ChIPs over 48h. From the supplemental file figure S2, the two replicates match only on the first day and don't on the second day. ChIP signals from the second replicate are weak from CT28 onwards unlike rep1 (which peaks even earlier at CT24). Given this variability, I fail to understand how it made the analysis more robust and allowed the detection of more than 4667 ~12h cycling events compared to 37 before. "This approach substantially improved statistical robustness, enabling the identification of 4,667 ~12 h cycling RBBP5 binding events (adjusted P < 0.05) and 5,366 events (P < 0.05), compared to only 37 (adjusted P < 0.05) and 1,865 (P < 0.05) sites in the initial analysis, respectively. The updated results are presented in Fig. 2, Figs. S2 and 3 and Table S1 after incorporating both independent runs."
As opposed to what is stated in the rebuttal above, the results in the main figure 2 have not been updated with these new and the numbers are identical to version R1.

We thank the reviewer for raising this concern. We addressed the rationale for the second temporal RBBP5 ChIP-seq experiment in detail during the first round of revision, but we recognize that our explanation may not have been sufficiently clear. We therefore clarify below why we chose a 4-h sampling interval over 48 h, how the two independent datasets were integrated.

**Review Figure 1**. **Cumulative distributions of adj P value of having 12h rhythm with different sampling strategy. The bottom is a zoomed in view from FDR 0 to FDR 0.1**

**Rationale for performing the second RBBP5 ChIP-seq experiment at 4-h resolution over 48 h.** To guide our experimental design in a cost-effective but statistically rigorous manner, we systematically evaluated the effects of sampling interval, experimental duration, and biological replication using our previously published temporal RNA-seq datasets, which were collected at 2-h resolution over 48 h with biological duplicates at each time point. We computationally subsampled these datasets to compare 2-h versus 4-h sampling intervals, 24-h versus 48-h duration, and the presence or absence of biological replicates (n = 1 versus n = 2). We then evaluated the performance of each design based on the cumulative distribution of adjusted P values for detection of ~12-h rhythms. We emphasize that the RNA-seq dataset was used here simply as a proof-of-principle synthetic dataset to evaluate temporal sampling strategies. The statistical consequences of sampling frequency, duration, and replication are independent of the specific molecular readout, and we used RNA-seq because, to our knowledge, no comparable ChIP-seq dataset with 2-h resolution over 48 h and biological duplicates are available for such systematic subsampling analysis.

As shown in **Review Fig. 1**, these analyses indicated that 2-h sampling over 24 h (the sampling strategy originally suggested by the reviewer) provided little statistical advantage over 4-h sampling over 48 h for detecting ~12-h rhythms. **In contrast, biological replication produced a substantially greater improvement in statistical power and robustness.** Indeed, **sampling every 4 h over 48 h with biological duplicates at each time point yielded greater statistical power than sampling every 2 h over 48 h with only one sample per time point** (compare the green and purple lines in Review Fig. 1).

Ideally, we would have repeated the entire RBBP5 ChIP-seq experiment at 2-h resolution over 48 h with biological duplicates at every time point, which would provide the greatest statistical power. However, such an experiment is prohibitively resource-intensive at this stage, particularly because the first three co-authors who performed the original experiments have all left the laboratory. We therefore pursued the most rigorous feasible alternative: we performed a second, completely independent temporal ChIP-seq experiment at 4-h resolution over 48 h and integrated it with the original dataset, generating a complete 48-h dataset with biological duplicates at every 4-h time point. This design provides independent biological replication across the entire 48-h time course while retaining sufficient temporal resolution for robust detection of ~12-h oscillations.

**Regarding the differences between the two RBBP5 ChIP-seq experiments**, we agree with the reviewer that the temporal profiles are not identical. However, the second experiment was intentionally performed as a fully independent biological replicate rather than as a technical replication of the first experiment. The two experiments used different biological samples, were performed by different individuals, and the libraries were prepared and sequenced by different sequencing providers. In addition, because this project began more than eight years ago while the corresponding author was still at Baylor College of Medicine, the sequencing technologies differed: the original experiment used single-end sequencing, whereas the second experiment used paired-end sequencing. Under these circumstances, some differences in absolute ChIP signal intensity and temporal dynamics are expected.

Importantly, despite these technical and biological differences, the major temporal features are reproducible between the two experiments. RBBP5 binding peaks are preferentially centered around CT0, CT12, CT24, and CT36 in both datasets, consistent with the temporal profile of nuclear RBBP5 protein abundance. Thus, although the amplitudes of individual peaks differ between experiments—including weaker signals at later time points in the second experiment—the overall ~12-h temporal pattern is reproducible.

To integrate the two independently generated datasets while minimizing differences in absolute ChIP signal caused by batch and sequencing-platform effects, we first performed **Z-score normalization within each dataset** and then applied the RAIN algorithm to the combined Z-score-normalized temporal profiles to identify ~12-h cycling RBBP5-binding sites. The substantial increase from 37 to 4,667 sites at adjusted P < 0.05 reflects the substantially greater statistical power obtained by incorporating an independent biological replicate across the 48-h time course, consistent with our subsampling analysis in Review Fig. 1. This information is included in the updated method section of the manuscript. The resulting data is presented in Fig. 1E.

Regarding Fig. 2, we apologize that our previous response was not sufficiently clear. The numbers shown in Fig. 2 do **not** represent the number of promoters exhibiting ~12-h RBBP5-binding rhythms. Rather, they represent genes classified according to the presence or absence of RBBP5 promoter binding and whether those genes exhibit ~12-h rhythms in gene expression as determined by RNA-seq. The second RBBP5 ChIP-seq dataset produced substantially stronger peak signals and greater sensitivity than the original dataset. Importantly, essentially all genes with RBBP5-binding TSS sites identified in the first dataset were recapitulated in the second dataset, whereas the second dataset identified sites in additional genes (see Fig. S2A for visualization). Consequently, incorporation of the second dataset greatly increased our statistical power for identifying 12h rhythmic RBBP5 binding, but did not materially alter the gene classifications (genes whose RBBP5 binding sites were identified in both sets of RBBP5 ChIP-seq were classified as RBBP5 target genes). With that said, we did revise the number of genes and exclude those genes with RBBP5 TSS binding but are not expressed in the liver. The revised number and statistics are shown in **Fig. 2A**. It does not change, but actually further strengthen the conclusion of the study: RBBP5 TSS binding is strongly associated with 12h rhythmic gene expression.

Finally, we would like to emphasize that the revised dataset consists of **two independent temporal RBBP5 ChIP-seq experiments spanning 48 h sampled at 4h resolution**, providing biological replication across the entire time course. Temporal ChIP-seq studies are experimentally demanding, and many published studies use sampling intervals of approximately 3–4 h, cover only a single 24-h cycle, and/or lack independent biological replication across the complete time course. We therefore believe that the current experimental design—48 h of temporal coverage with independent biological duplicates and 4-h resolution—provides a rigorous dataset for assessing ~12-h RBBP5-binding rhythms. Together with the concordant ~12-h phase structure observed across the two independent experiments and the statistical evaluation presented in Review Fig. 1, we believe these data provide robust support for our conclusions.

Point 2. I don't fully agree with response to point 2 .

(In this study, we uncovered that unlike the circadian clock, which was predominantly regulated by histone acetyltransferase (HAT) [87-89] and histone deacetylase (HDAC) [85, 90, 91], the epigenome of the 12h oscillator is associated with 12h H3K4me3 oscillation at the proximal promoters of UPR genes.)

While it is toned down from the original statement,  to say that circadian gene expression is "predominantly regulated" only by acetylation dynamics is a stretch...

We revised this text by stating “we uncovered that unlike the circadian clock, which was **modulated** by histone acetyltransferase (HAT) [87-89] and histone deacetylase (HDAC) [85, 90, 91]”.

Point 3.

I encourage the authors to insert the review figure 6C in the main figure panel 3H.

Additionally, do we know what the protein levels  of Xbp1 are in the liver during the time course? While I can understand that studying the dynamics of the complex would be confounded by the absence of Xbp1 at some time points- is Xbp1 still targeted to chromatin?

The authors say "their data supports a model in which RBBP5 functions
within the chromatin landscape that assists XBP1s-dependent rhythmic transcription of target genes"

While its slightly dampened, it doesnt seem like Xbp1 expression rhythms are so different in the LKO mice ( a couple of time points have high variance in review figure 3c).
 
So, is XBP1 still targeted to chromatin but not able to drive rhythmic 12h transcription of its targets in the absence of RBBP5?

As requested, we performed western blot of XBP1s as well as ChIP-qPCR of XBP1s in both WT and RBBP5 *^LKO^* mice. We added the results as follows:

Lastly, since XBP1s is a major transcriptional regulator of the hepatic 12h oscillator [20, 22], we examined how RBBP5 regulates its temporal expression. In the liver of *Rbbp5* *^LKO^* mice, the 12h oscillations of *Xbp1s* mRNA and protein were impaired, accompanied by a reduction in mean daily XBP1s expression **(Fig. 3J, K**). We next performed XBP1s ChIP-qPCR at CT12, when hepatic XBP1s protein levels were comparable between WT and *Rbbp5* *^LKO^* mice. Hepatic XBP1s occupancy at the promoters of canonical 12h UPR genes was comparable between the two genotypes (**Fig. 3L**). These results suggest that RBBP5 regulates the temporal expression of XBP1s but is likely not required for XBP1s chromatin recruitment, consistent with its role as a transcriptional co-regulator.

Minor point 4:

Its essential to visualize the RBBP5 ChIP-Seq on the non-12h cycling gene set in main Figure 2  to support their claim that "RBBP5 chromatin binding is stronger and exhibits more robust ~12h rhythmicity at sites associated with 12h cycling genes compared to sites linked to genes lacking ~12h rhythmicity".

The authors can add the pval plots as well in the main figure panels to drive home the point.

As requested, we have added the heatmap and corresponding *P* values to the main **Fig. 2C and D**. For statistical analysis, we used the −log10-transformed *P* values. We also revised the data to reflect the updated gene numbers shown in **Fig. 2A**.
