# Supplementary material for: The histone methyltransferase complex subunit RBBP5 serves as a central epigenetic regulator of proteostasis dynamics
Source: PLoS Biol. 2026 Oct 8;24(10):e3004048. doi: 10.1371/journal.pbio.3004048 (PMC13667922; doi:10.1371/journal.pbio.3004048)
Supplement: Peer review history

# Editor report: Decision Letter 0

**DOI:** 10.1371/journal.pbio.3004048.r001

**Authors:** Richard Hodge

24 Oct 2025

Dear Dr Zhu,

Thank you for submitting your manuscript entitled "Epigenetic control of proteostasis dynamics by RBBP5–mediated H3K4 trimethylation" for consideration as a Research Article by PLOS Biology. Please accept my sincere apologies for the delay in getting back to you as we consulted with an academic editor about your submission.

Your manuscript has now been evaluated by the PLOS Biology editorial staff, as well as by an academic editor with relevant expertise, and I am writing to let you know that we would like to send your submission out for external peer review.

However, before we can send your manuscript to reviewers, we need you to complete your submission by providing the metadata that is required for full assessment. To this end, please login to Editorial Manager where you will find the paper in the 'Submissions Needing Revisions' folder on your homepage. Please click 'Revise Submission' from the Action Links and complete all additional questions in the submission questionnaire.

Once your full submission is complete, your paper will undergo a series of checks in preparation for peer review. After your manuscript has passed the checks it will be sent out for review. To provide the metadata for your submission, please Login to Editorial Manager (https://www.editorialmanager.com/pbiology) within two working days, i.e. by Oct 26 2025 11:59PM.

If your manuscript has been previously peer-reviewed at another journal, PLOS Biology is willing to work with those reviews in order to avoid re-starting the process. Submission of the previous reviews is entirely optional and our ability to use them effectively will depend on the willingness of the previous journal to confirm the content of the reports and share the reviewer identities. Please note that we reserve the right to invite additional reviewers if we consider that additional/independent reviewers are needed, although we aim to avoid this as far as possible. In our experience, working with previous reviews does save time.

If you would like us to consider previous reviewer reports, please edit your cover letter to let us know and include the name of the journal where the work was previously considered and the manuscript ID it was given. In addition, please upload a response to the reviews as a 'Prior Peer Review' file type, which should include the reports in full and a point-by-point reply detailing how you have or plan to address the reviewers' concerns.

During the process of completing your manuscript submission, you will be invited to opt-in to posting your pre-review manuscript as a bioRxiv preprint. Visit http://journals.plos.org/plosbiology/s/preprints for full details. If you consent to posting your current manuscript as a preprint, please upload a single Preprint PDF.

Feel free to email us at plosbiology@plos.org if you have any queries relating to your submission.

Kind regards,

Richard

Richard Hodge, PhD

Senior Editor, PLOS Biology

rhodge@plos.org

PLOS

Empowering researchers to transform science

Carlyle House, Carlyle Road, Cambridge, CB4 3DN, United Kingdom

California (U.S.) corporation #C2354500, based in San Francisco

# Peer review: Decision Letter 1

**DOI:** 10.1371/journal.pbio.3004048.r002

**Authors:** Richard Hodge

19 Dec 2025

Dear Bokai,

Thank you for your patience while we considered your revised manuscript "Epigenetic control of proteostasis dynamics by RBBP5–mediated H3K4 trimethylation" for publication as a Research Article at PLOS Biology. Please accept my sincere apologies for the delays that you have experienced during the peer review process Your revised study has been evaluated by the PLOS Biology editors, the Academic Editor and two independent reviewers.

In light of the reviews, which you will find at the end of this email, we would like to invite you to revise the work to thoroughly address the reviewers' reports.

As you can see, the reviewers agree that your revised manuscript is interesting and well done, but they raise overlapping concerns with the overall strength of the evidence for a clearly defined 12 h oscillator. Specifically, Reviewer #2 notes that the temporal resolution of the ChIP-seq analysis for RBBP5 should be improved, particularly the use of a 2-hour sampling interval. After discussions with the academic editor, we agree that addressing this point would be valuable, especially in light of the broader concerns raised by Reviewer 1 (with respect to Fig. 1C and 2B) regarding the statistical robustness and interpretation of the reported 12-hour rhythms. In addition, Reviewer #1 raises concerns about the rigor of the statistical analyses and notes that the manuscript should be more cautious in its interpretation and framing of the data.

Given the extent of revision needed, we cannot make a decision about publication until we have seen the revised manuscript and your response to the reviewers' comments. Your revised manuscript is likely to be sent for further evaluation by all or a subset of the reviewers.

In addition to these revisions, you will need to complete some formatting changes, which you will receive in a follow up email. A member of our team will be in touch with a set of requests shortly.

We expect to receive your revised manuscript within 3 months. Please email us (plosbiology@plos.org) if you have any questions or concerns, or would like to request an extension.

At this stage, your manuscript remains formally under active consideration at our journal; please notify us by email if you do not intend to submit a revision so that we may withdraw it.

**IMPORTANT - SUBMITTING YOUR REVISION**

Your revisions should address the specific points made by each reviewer. Please submit the following files along with your revised manuscript:

1. A 'Response to Reviewers' file - this should detail your responses to the editorial requests, present a point-by-point response to all of the reviewers' comments, and indicate the changes made to the manuscript.

*NOTE: In your point-by-point response to the reviewers, please provide the full context of each review. Do not selectively quote paragraphs or sentences to reply to. The entire set of reviewer comments should be present in full and each specific point should be responded to individually, point by point.

You should also cite any additional relevant literature that has been published since the original submission and mention any additional citations in your response.

2. In addition to a clean copy of the manuscript, please also upload a 'track-changes' version of your manuscript that specifies the edits made. This should be uploaded as a "Revised Article with Changes Highlighted" file type.

*Re-submission Checklist*

When you are ready to resubmit your revised manuscript, please refer to this re-submission checklist: https://plos.io/Biology_Checklist

To submit a revised version of your manuscript, please go to https://www.editorialmanager.com/pbiology/ and log in as an Author. Click the link labelled 'Submissions Needing Revision' where you will find your submission record.

Please make sure to read the following important policies and guidelines while preparing your revision:

*Published Peer Review*

Please note while forming your response, if your article is accepted, you may have the opportunity to make the peer review history publicly available. The record will include editor decision letters (with reviews) and your responses to reviewer comments. If eligible, we will contact you to opt in or out. Please see here for more details:

https://blogs.plos.org/plos/2019/05/plos-journals-now-open-for-published-peer-review/

*PLOS Data Policy*

Please note that as a condition of publication PLOS' data policy (http://journals.plos.org/plosbiology/s/data-availability) requires that you make available all data used to draw the conclusions arrived at in your manuscript. If you have not already done so, you must include any data used in your manuscript either in appropriate repositories, within the body of the manuscript, or as supporting information (N.B. this includes any numerical values that were used to generate graphs, histograms etc.). For an example see here: http://www.plosbiology.org/article/info%3Adoi%2F10.1371%2Fjournal.pbio.1001908#s5

*Blot and Gel Data Policy*

We require the original, uncropped and minimally adjusted images supporting all blot and gel results reported in an article's figures or Supporting Information files. We will require these files before a manuscript can be accepted so please prepare them now, if you have not already uploaded them. Please carefully read our guidelines for how to prepare and upload this data: https://journals.plos.org/plosbiology/s/figures#loc-blot-and-gel-reporting-requirements

*Protocols deposition*

To enhance the reproducibility of your results, we recommend that if applicable you deposit your laboratory protocols in protocols.io, where a protocol can be assigned its own identifier (DOI) such that it can be cited independently in the future. Additionally, PLOS ONE offers an option for publishing peer-reviewed Lab Protocol articles, which describe protocols hosted on protocols.io. Read more information on sharing protocols at https://plos.org/protocols?utm_medium=editorial-email&utm_source=authorletters&utm_campaign=protocols

Thank you again for your submission to our journal. We hope that our editorial process has been constructive thus far, and we welcome your feedback at any time. Please don't hesitate to contact us if you have any questions or comments.

Kind regards,

Richard

Richard Hodge, PhD

Senior Editor, PLOS Biology

rhodge@plos.org

------------------------------------

REVIEWS:

Reviewer #1: The data in this manuscript are really interesting and it should be published. Clear evidence is presented that RBBP5 is required for many transcriptional responses in cells and in vivo. However, the evidence causally connecting implicating RBBP5 as part of a putative ~12h ultradian clock is less than convincing and I'm concerned that the authors overlook other plausible interpretations in favour of their preferred hypothesis. I also have reservations about the way in which these findings are communicated:

1. The liver of a freely behaving mouse is not under constant conditions. In addition to any cell-autonomous regulatory processes, there will be daily and higher frequency fluctuations in feeding, body temperature and various hormonal cues. Therefore, there is no reason to believe that the abundance of any given mRNA or transcription at any given genomic locus would be constant over time. Given previous reports (e.g., Hughes et al, 2009; Cretenet et al, 2012), its clear that some of this variation has a ~12h periodicity. The function and mechanism of these ~12h oscillations is the focus of this paper.

I am afraid that I am not as confident as the authors that "many of these ~12h ultradian rhythms are established by an XBP1s-dependent 12h ultradian oscillator separate from ... the ~24h circadian clock ..." What they have shown is that ~12h oscillations are observed in the absence of Bmal1, but so also are many circadian rhythms, e.g., Lipton et al., 2015; O'Neill and Reddy, 2011. Moreover, Cretenet et al found that ~12h oscillations were severely disrupted in Cry1/2-deficient mice. Therefore, please refine this claim. I also find the phrase "12h oscillator" ambiguous, because it suggests self-sufficiency and established independence from any other known mechanism, whereas I do not believe that this is widely accepted to be the case (e.g. Emery & Gachon, 2025).

The clear communication of their work would also be improved immensely if the authors could be more explicit to distinguish what they have actually shown from their speculation and interpretation. In particular, it would be more accurate and balanced if they were to make clear it is their hypothesis that ~12h oscillations are driven by a dedicated ~12h oscillator, rather than an established fact. This is important because to do otherwise would discount the possibility that there might be other explanations for their observations.

The way to test the authors' assertion that 12h oscillations are driven by a XBP1s-dependent oscillator independent of the cell or circadian cycle, would be to clamp XBP1s activity at a constant level within the normal range of physiological activity and then assess whether any other 12h oscillations remain downstream. Knocking out genes does not test this hypothesis, and cannot rule out epistasis. This is not a criticism of their work, but of its reasonable interpretation. The authors might be entirely correct in their interpretation, but the evidence presented is not strong enough to rule out other interpretations so they simply need to tone it down and add caveats.

2. There is an implicit assumption that these 12h rhythms in transcription/transcript abundance are functional rather than consequences of something else. I am not sure that this has been clearly established. For example, to my mind it is quite plausible that these 12h oscillations are epiphenomena of homeostatic processes and do not "drive" anything, i.e., what is the evidence that the abundance of any 12h oscillating species (mRNA, protein, metabolite) becomes rate-limiting for the activity of some downstream process at its nadir. To provide an example, outside of fasting, physiological blood glucose levels exhibit both daily and ultradian rhythms but, because it is homeostatically-regulated, there is no reason to believe that the availability of extracellular glucose is ever rate-limiting for the vast majority of glucose-dependent processes.

3. It is not clear to me what is the unappreciated epigenetic timing mechanism that the authors believe they have identified (see abstract).

4. An alternative explanation for the authors' observations is that RBBP5 is an essential or conditionally essential gene that regulates transcription globally and promiscuously as part of the KMT2 family of methyltransferase complexes. Interfering with RBBP5 function therefore globally disrupts transcription of genes that are regulated by H3K4me, which includes many genes encoding proteins that regulate protein homeostasis. Thus, what evidence argues against the possibility that loss of RBBP5 may simply be epistatic to many transcriptional responses and programs.

All figures - to save the reader having to look it up each time, it would be helpful to state in each relevant figure legend whether the mice were in LD or DD conditions. If LD, when was lights on (ZT0)?

Fig 1B. If the idea is that there is change over time then it seems to be missing an appropriate statistical test.

Fig 1C. Is it a subjective judgement or is there some statistical analysis to support the claim of robust 12h rhythms please?

Fig 1E. Presented as continuous data but isn't. Fine to show a trend line but please also show the data points so that the variance and temporal resolution is clear.

Fig S1H. It would be helpful to see a graph comparing the relative amplitudes of "rhythmic genes". This would allow the reader to assess the extent of biological variation, rather just how many significant rhythms there are.

Fig S1D. It is not sufficiently strong evidence to claim a ~12h rhythm in nuclear RBBP5. Please show replicates, whole cell RBBP5, appropriate loading control and appropriate statistical analysis.

Fig 2A. Not clear what a non-12h cycling gene is. Does this mean it cycles but not with 12h periodicity, or that it doesn't cycle. How was each gene's promoter defined please? Please consider whether the correlation might be explained by a third variable, e.g., RBBP5 binding correlates with expression level, expression level correlates with 12h cycling.

Fig 2B. All the RBBP5 ChiP-seq genes seem to be in the same phase, whereas nearly half of the RNA-seq genes look to be in antiphase. A phase scatterplot of Chip-seq vs RNA-Seq phase would be helpful here to test the authors' suggestion that the former is "driving" the latter. How well do they correlate. Similarly a relative amplitude scatterplot for Chip-seq vs RNA-Seq would provide additional insight.

Fig 2E. Some of these are overexposed. Good practise guidelines dictate the input should really be on the same blot as the IP, without cropping the bands. Where can the raw blots be assessed please?

Fig 3B. Fig S2C shows there is detected in the CRE =/- liver. This is difficult to reconcile with Fig 3B. Is the same antibody being used for the IF and western blot? If not, what region of the protein does each recognise. Is this really a partial LoF rather than full KO?

Fig S2C. Where can the raw blots be assessed please?

Fig 3C. Doesn't instill confidence in the analytical approach by claiming there are thousands of genes with periodicities >30h. I've used RAIN, it identifies loads of false positives. The claim that it robustly uncovers ultradian rhythms is spurious. The reader would have more confidence if a higher level of stringency were applied to the rhythmicity analysis.

Fig 3G. What is a superimposed ~12h rhythm please?

Fig S6A. Error bars too faint for me to see. What is the null hypothesis please? The three traces look very similar to my eyes. What is the p-value for fitting a straight line vs a 12h damped sine wave?

Fig S6B. Please check x-axis labelling. It would be helpful to see the raw data before detrending and some more examples.

Fig 4F. I don't understand how RBBP5 binding can be measured in the KO unless it is an incomplete KO.

Fig 4. Where can these blots be assessed please? Why is the ponceau statin black and white in G and coloured in H. A more traditional loading control might be better. Is there quantification for these blots please?

Line 260. "Before the discovery of the mammalian 12h oscillator, the dynamics of proteostasis were primarily studied as transient responses to various proteotoxic stresses, with a classic example being the UPR triggered by ER stress." ....without wanting to in any way diminish the authors' work, I do not believe their research has been responsible for any paradigm shift in the wider proteostasis field. I could be wrong of course. I already expressed by skepticism about whether it is widely accepted that a mammalian 12h oscillator has been discovered, but it is certainly an interesting hypothesis.

Line 312. " RBBP5 is both necessary and sufficient for the transcriptional regulation of hepatic response to acute proteotoxic stress in vivo". I cannot imagine the authors are really suggested that RBBP5 alone is sufficient for this. The rest of the complex and downstream mechanisms are obviously going to be necessary also. Please rephrase and think about there is any stronger evidence that might support this claim - the data presented suggests that some transcriptional responses to tunicamycin are modulated by the abundance of RBBP5 .... however the over-expression experiment is difficult to understand because I can't see any strong evidence that RBBP5 is over-expressed in 4H - please show all the blots with quantification. If RBBP5 were over-expressed, how would this lead to increased COMPASS activity without commensurate expression of the other complex subunits?

Fig 5A. Which are the MEF and which are the liver data?

Discussion - if the authors are receptive to my suggestions I expect that the discussion will change somewhat so I won't provide any feedback on it.

Reviewer #2: Major comments:

The paper by Kubra et al is an exhaustive manuscript investigating the role of RBBP5 protein in ultradian rhythms in the liver. The experiments are well done and there are many striking findings within the manuscript, but in some cases they lack depth - and the claims made at the end are not sufficiently backed up.

Point 1: As they say, in line 472 of the discussion - "the epigenome of the mammalian ~12h ultradian oscillator remains completely unexplored" - yet they perform a 4h resolution ChIP-seq over 48 hours for RBBP5 - a typical circadian experiment rather than performing a 2hr resolution over 24h, which would be more appropriate for determining rhythms from an ultradian oscillator.

Point 2: In Line 419-422 of the discussion, the authors state that the "epigenome of the 12h oscillator is marked by COMPASS-mediated histone methylation"- and that "loss of one of the essential subunits of COMPASS, RBBP5, abolished more than 90% of the hepatic ~12h transcriptome".

For this statement to be valid, the definitive experiment - a H3K4Me3 ChIPSeq in RBBP5 LKO mice (with a 2hr resolution) - is missing. Would the ultradian genes specifically lose K4Me3 rhythms? "while having no effects on the core circadian clock"

Point 3: In Fig2e, the authors show the XBP1s co-IP with RBBP5. Does it dynamically assemble/disassemble over 12hrs? What is the state of this complex in RBBP5 LKO mice? Does Xbp1 fail to target genes with a 12h rhythm in the RBBP5 LKO mice? What about the other components identified by proximity ligation? Are any of them specific to the epigenome of the ultradian oscillator? The final dataset of remodelers almost seems like a throwaway experiment - which is unfortunate.

Minor Point 4: A global criticism of the figures are the font sizes for some of the text within panels are just too small and impossible to read - ; TSS labels in 1C; gene names in Fig4d, X axis labels in Fig5a among others-

Minor point 5: In Fig2, it would be important to plot and visualize RBBP5 ChIP-Seq on the 3926 non-12h cycling gene set to show that their patterns differ from the heat map for the 12h cycling gene set of 2525 genes shown in panel 2B.

Minor errors: Fig S6B- axis label needs correction for time

# Author comment: Author response to Decision Letter 2

**DOI:** 10.1371/journal.pbio.3004048.r003

28 Apr 2026

Attachment Submitted filename: Rebuttal.pdf

# Peer review: Decision Letter 2

**DOI:** 10.1371/journal.pbio.3004048.r004

**Authors:** Richard Hodge

17 Jun 2026

Dear Dr Zhu,

Thank you for your patience while we considered your revised manuscript "Epigenetic control of proteostasis dynamics by RBBP5–mediated H3K4 trimethylation" for publication as a Research Article at PLOS Biology. Please accept my sincere apologies for the delays that you have experienced during this round of the peer review process. Your revised study has been evaluated by the PLOS Biology editors, the Academic Editor and the original reviewers.

In light of the reviews, which you will find at the end of this email, we would like to invite you to revise the work to thoroughly address the reviewers' reports.

As you can see, although both reviewers agree that several points have been addressed during revision, they note that several of their primary concerns remain. Specifically, Reviewer #1 continues to raise concerns that the functional consequences of the 12 hour ultradian rhythms are unclear. Reviewer #2 raises concerns about the inclusion of the second ChIP-seq experiment at 4 h resolution over 48 hours, rather than a 2 hour sampling interval as requested. In addition, Reviewer #2 raises concerns regarding overstatements in the manuscript text, and asks for additional data to visualise the RBBP5 ChIP-Seq on the non-12h cycling gene set in Figure 2.

After discussions with an academic editor, we think that the revised version should fully address the remaining four points of Reviewer #2. In addition, we agree with the reviewer that several conclusions appear stronger than what the data can directly support, so we ask that the central conclusions are reframed, with clearer acknowledgment of the limitations and a clearer separation between experimental observations and hypothesis.

Given the extent of revision needed, we cannot make a decision about publication until we have seen the revised manuscript and your response to the reviewers' comments. Your revised manuscript is likely to be sent for further evaluation by all or a subset of the reviewers.

In addition to these revisions, you will need to complete some formatting changes, which you will receive in a follow up email. A member of our team will be in touch with a set of requests shortly.

We expect to receive your revised manuscript within 3 months. Please email us (plosbiology@plos.org) if you have any questions or concerns, or would like to request an extension.

At this stage, your manuscript remains formally under active consideration at our journal; please notify us by email if you do not intend to submit a revision so that we may withdraw it.

**IMPORTANT - SUBMITTING YOUR REVISION**

Your revisions should address the specific points made by each reviewer. Please submit the following files along with your revised manuscript:

1. A 'Response to Reviewers' file - this should detail your responses to the editorial requests, present a point-by-point response to all of the reviewers' comments, and indicate the changes made to the manuscript.

*NOTE: In your point-by-point response to the reviewers, please provide the full context of each review. Do not selectively quote paragraphs or sentences to reply to. The entire set of reviewer comments should be present in full and each specific point should be responded to individually, point by point.

You should also cite any additional relevant literature that has been published since the original submission and mention any additional citations in your response.

2. In addition to a clean copy of the manuscript, please also upload a 'track-changes' version of your manuscript that specifies the edits made. This should be uploaded as a "Revised Article with Changes Highlighted" file type.

*Re-submission Checklist*

When you are ready to resubmit your revised manuscript, please refer to this re-submission checklist: https://plos.io/Biology_Checklist

To submit a revised version of your manuscript, please go to https://www.editorialmanager.com/pbiology/ and log in as an Author. Click the link labelled 'Submissions Needing Revision' where you will find your submission record.

Please make sure to read the following important policies and guidelines while preparing your revision:

*Published Peer Review*

Please note while forming your response, if your article is accepted, you may have the opportunity to make the peer review history publicly available. The record will include editor decision letters (with reviews) and your responses to reviewer comments. If eligible, we will contact you to opt in or out. Please see here for more details:

https://blogs.plos.org/plos/2019/05/plos-journals-now-open-for-published-peer-review/

*PLOS Data Policy*

Please note that as a condition of publication PLOS' data policy (http://journals.plos.org/plosbiology/s/data-availability) requires that you make available all data used to draw the conclusions arrived at in your manuscript. If you have not already done so, you must include any data used in your manuscript either in appropriate repositories, within the body of the manuscript, or as supporting information (N.B. this includes any numerical values that were used to generate graphs, histograms etc.). For an example see here: http://www.plosbiology.org/article/info%3Adoi%2F10.1371%2Fjournal.pbio.1001908#s5

*Blot and Gel Data Policy*

We require the original, uncropped and minimally adjusted images supporting all blot and gel results reported in an article's figures or Supporting Information files. We will require these files before a manuscript can be accepted so please prepare them now, if you have not already uploaded them. Please carefully read our guidelines for how to prepare and upload this data: https://journals.plos.org/plosbiology/s/figures#loc-blot-and-gel-reporting-requirements

*Protocols deposition*

To enhance the reproducibility of your results, we recommend that if applicable you deposit your laboratory protocols in protocols.io, where a protocol can be assigned its own identifier (DOI) such that it can be cited independently in the future. Additionally, PLOS ONE offers an option for publishing peer-reviewed Lab Protocol articles, which describe protocols hosted on protocols.io. Read more information on sharing protocols at https://plos.org/protocols?utm_medium=editorial-email&utm_source=authorletters&utm_campaign=protocols

Thank you again for your submission to our journal. We hope that our editorial process has been constructive thus far, and we welcome your feedback at any time. Please don't hesitate to contact us if you have any questions or comments.

Best regards,

Richard

Richard Hodge, PhD

Senior Editor, PLOS Biology

rhodge@plos.org

------------------------------------

REVIEWS:

Reviewer #1: Thanks for trying to address my feedback. I note that my second point about functional consequences has not been engaged with but that's your call and it shouldn't be a barrier to publishing the paper. At some point you will need to engage with whether these 12h rhythms have any actual consequence for cellular or tissue function, otherwise most readers (myself including) will continue to assume that they are epiphenomena of protein homeostasis mechanisms, i.e., the periodicity arises because the perturbation is periodic.

Reviewer #2: I thank the authors for addressing my points but my primary concerns remain.

Point 1: regarding the argument against a higher- time resolution chip for RBBP5.

I can't understand why the authors preferred a 4hr window for a new set of duplicate ChIPs over 48h.

From the supplemental file figure S2, the two replicates match only on the first day and don't on the second day.

ChIP signals from the second replicate are weak from CT28 onwards unlike rep1 (which peaks even earlier at CT24). Given this variability, I fail to understand how it made the analysis more robust and allowed the detection of more than 4667 ~12h cycling events compared to 37 before.

"This approach substantially improved statistical robustness, enabling the

identification of 4,667 ~12 h cycling RBBP5 binding events (adjusted P < 0.05) and 5,366 events (P < 0.05), compared to only 37 (adjusted P < 0.05) and 1,865 (P < 0.05) sites in the initial analysis, respectively. The updated results are presented in Fig. 2, Figs. S2 and 3 and Table S1 after incorporating both independent runs."

As opposed to what is stated in the rebuttal above, the results in the main figure 2 have not been updated with these new and the numbers are identical to version R1.

Point 2. I don't fully agree with response to point 2 .

(In this study, we uncovered that unlike the circadian clock, which was predominantly regulated by histone acetyltransferase (HAT) [87-89] and histone deacetylase (HDAC) [85, 90, 91], the epigenome of the 12h oscillator is associated with 12h H3K4me3 oscillation at the proximal promoters of UPR genes.)

While it is toned down from the original statement, to say that circadian gene expression is "predominantly regulated" only by acetylation dynamics is a stretch...

Point 3.

I encourage the authors to insert the review figure 6C in the main figure panel 3H.

Additionally, do we know what the protein levels of Xbp1 are in the liver during the time course? While I can understand that studying the dynamics of the complex would be confounded by the absence of Xbp1 at some time points- is Xbp1 still targeted to chromatin?

The authors say "their data supports a model in which RBBP5 functions

within the chromatin landscape that assists XBP1s-dependent rhythmic transcription of target genes"

While its slightly dampened, it doesnt seem like Xbp1 expression rhythms are so different in the LKO mice ( a couple of time points have high variance in review figure 3c).

So, is XBP1 still targeted to chromatin but not able to drive rhythmic 12h transcription of its targets in the absence of RBBP5?

Minor point 4:

Its essential to visualize the RBBP5 ChIP-Seq on the non-12h cycling gene set in main Figure 2 to support their claim that "RBBP5 chromatin binding is stronger and exhibits more robust ~12h rhythmicity at sites associated with 12h cycling genes compared to sites linked to genes lacking ~12h rhythmicity".

The authors can add the pval plots as well in the main figure panels to drive home the point.

# Author comment: Author response to Decision Letter 3

**DOI:** 10.1371/journal.pbio.3004048.r005

15 Sep 2026

Attachment Submitted filename: rebuttal for review comments 2.docx

# Editor report: Decision Letter 3

**DOI:** 10.1371/journal.pbio.3004048.r006

**Authors:** Richard Hodge

21 Sep 2026

Dear Bokai,

Thank you for your patience while we considered your revised manuscript "Epigenetic control of proteostasis dynamics by RBBP5–mediated H3K4 trimethylation" for publication as a Research Article at PLOS Biology. This revised version of your manuscript has been evaluated by the PLOS Biology editors and the Academic Editor.

Based on our Academic Editor's assessment of your revision, I am pleased to say that we are likely to accept this manuscript for publication, provided you satisfactorily address the following data and other policy-related requests (A-H):

(A) We routinely suggest changes to titles to ensure maximum accessibility for a broad, non-specialist readership. In this case, we suggest a minor edit to the title, as follows, to provide some additional information for the function of RBBP5. Please ensure you change both the manuscript file and the online submission system, as they need to match for final acceptance:

“The histone methyltransferase complex subunit RBBP5 serves as a central epigenetic regulator of proteostasis dynamics”

(B) You may be aware of the PLOS Data Policy, which requires that all data be made available without restriction: http://journals.plos.org/plosbiology/s/data-availability. For more information, please also see this editorial: http://dx.doi.org/10.1371/journal.pbio.1001797

Note that we do not require all raw data. Rather, we ask that all individual quantitative observations that underlie the data summarized in the figures and results of your paper be made available in one of the following forms:

-Supplementary files (e.g., excel). Please ensure that all data files are uploaded as 'Supporting Information' and are invariably referred to (in the manuscript, figure legends, and the Description field when uploading your files) using the following format verbatim: S1 Data, S2 Data, etc. Multiple panels of a single or even several figures can be included as multiple sheets in one excel file that is saved using exactly the following convention: S1_Data.xlsx (using an underscore).

-Deposition in a publicly available repository. Please also provide the accession code or a reviewer link so that we may view your data before publication.

Regardless of the method selected, please ensure that you provide the individual numerical values that underlie the summary data displayed in the following figure panels as they are essential for readers to assess your analysis and to reproduce it:

Figure 1B-F, 2D-F, 3C-E, 3H-L, 4B-I, 5A, 5C-F, 5I, 6D-E, S1B-F, S2A-F, S3, S4D, S5A-I, S7A-C, S7E-G, S8A-E, S9A-C, S10A-H, S12B-D, S12H, S13A-B, S13E, S13G, S14C-E

NOTE: the numerical data provided should include all replicates AND the way in which the plotted mean and errors were derived (it should not present only the mean/average values).

(C) Please also ensure that each of the relevant figure legends in your manuscript include information on *WHERE THE UNDERLYING DATA CAN BE FOUND*, and ensure your supplemental data file/s has a legend.

(D) Thank you for providing the RNA-seq and ChIP-seq datasets in the GEO (GSE276155, GSE276157, GSE290140 and GSE276159) and the MS-proteomics data in the ProteomeXchange (PXD061069). However, I searched for the ProteomeXchange deposition and could not find it. Could you please check that the PXD061069 accession number is correct or ensure that the data is made publicly available at this stage before publication.

(E) We require the original, uncropped and minimally adjusted images supporting all blot and gel results reported in the following Figures:

Figure 2G, 3K, 4B, 4G-H, 5H, 6B, S1D, S4B-C, S12A, S12F-G, S13F, S14A

We will require these files before a manuscript can be accepted so please prepare and upload them now. Please carefully read our guidelines for how to prepare and upload this data: https://journals.plos.org/plosbiology/s/figures#loc-blot-and-gel-reporting-requirements.

(F) Per journal policy, if you have generated any custom code during the course of this investigation, please make it available without restrictions. Please ensure that the code is sufficiently well documented and reusable, and that your Data Statement in the Editorial Manager submission system accurately describes where your code can be found. More information on our Code Policy, what and how to share can be found here: https://journals.plos.org/plosbiology/s/code-availability

Please note that we cannot accept sole deposition of code in GitHub, as this could be changed after publication. However, you can archive this version of your publicly available GitHub code to Zenodo. Once you do this, it will generate a DOI number, which you will need to provide in the Data Accessibility Statement (you are welcome to also provide the GitHub access information). See the process for doing this here: https://docs.github.com/en/repositories/archiving-a-github-repository/referencing-and-citing-content

(G) Please ensure that your Data Statement in the submission system accurately describes where your data can be found and is in final format, as it will be published as written there.

(H) Please ensure that you are using best practice for statistical reporting and data presentation. These are our guidelines https://journals.plos.org/plosbiology/s/best-practices-in-research-reporting#loc-statistical-reporting and a useful resource on data presentation https://journals.plos.org/plosbiology/article?id=10.1371/journal.pbio.1002128

- If you are reporting experiments where n ≤ 5, please plot each individual data point.

------------------------------------------------------------------------

As you address these items, please take this last chance to review your reference list to ensure that it is complete and correct. If you have cited papers that have been retracted, please include the rationale for doing so in the manuscript text, or remove these references and replace them with relevant current references. Any changes to the reference list should be mentioned in the cover letter that accompanies your revised manuscript.

In addition to these revisions, you may need to complete some formatting changes, which you will receive in a follow up email. A member of our team will be in touch with a set of requests shortly. If you do not receive a separate email within a few days, please assume that checks have been completed, and no additional changes are required.

We expect to receive your revised manuscript within two weeks.

To submit your revision, please go to https://www.editorialmanager.com/pbiology/ and log in as an Author. Click the link labelled 'Submissions Needing Revision' to find your submission record. Your revised submission must include the following:

- a cover letter that should detail your responses to any editorial requests, if applicable, and whether changes have been made to the reference list

- a Response to Reviewers file that provides a detailed response to the reviewers' comments (if applicable, if not applicable please do not delete your existing 'Response to Reviewers' file.)

- a track-changes file indicating any changes that you have made to the manuscript.

NOTE: If Supporting Information files are included with your article, note that these are not copyedited and will be published as they are submitted. Please ensure that these files are legible and of high quality (at least 300 dpi) in an easily accessible file format. For this reason, please be aware that any references listed in an SI file will not be indexed. For more information, see our Supporting Information guidelines:

https://journals.plos.org/plosbiology/s/supporting-information

*Published Peer Review History*

Please note that you may have the opportunity to make the peer review history publicly available. The record will include editor decision letters (with reviews) and your responses to reviewer comments. If eligible, we will contact you to opt in or out. Please see here for more details:

https://plos.org/published-peer-review-history/

*Press*

Should you, your institution's press office or the journal office choose to press release your paper, please ensure you have opted out of Early Article Posting on the submission form. We ask that you notify us as soon as possible if you or your institution is planning to press release the article.

*Protocols deposition*

To enhance the reproducibility of your results, we recommend that if applicable you deposit your laboratory protocols in protocols.io, where a protocol can be assigned its own identifier (DOI) such that it can be cited independently in the future. Additionally, PLOS ONE offers an option for publishing peer-reviewed Lab Protocol articles, which describe protocols hosted on protocols.io. Read more information on sharing protocols at https://plos.org/protocols?utm_medium=editorial-email&utm_source=authorletters&utm_campaign=protocols

Please do not hesitate to contact me should you have any questions.

Best regards,

Richard

Richard Hodge, PhD

Senior Editor, PLOS Biology

rhodge@plos.org

PLOS

Empowering researchers to transform science

Carlyle House, Carlyle Road, Cambridge, CB4 3DN, United Kingdom

California (U.S.) corporation #C2354500, based in San Francisco

# Author comment: Author response to Decision Letter 4

**DOI:** 10.1371/journal.pbio.3004048.r007

24 Sep 2026

Attachment Submitted filename: rebuttal_for_review_comments_2_auresp_4.docx

# Editor report: Decision Letter 4

**DOI:** 10.1371/journal.pbio.3004048.r008

**Authors:** Richard Hodge

25 Sep 2026

Dear Bokai,

On behalf of my colleagues and the Academic Editor, Achim Kramer, I am pleased to say that we can accept your manuscript for publication, provided you address any remaining formatting and reporting issues. These will be detailed in an email you should receive within 2-3 business days from our colleagues in the journal operations team; no action is required from you until then. Please note that we will not be able to formally accept your manuscript and schedule it for publication until you have completed any requested changes.

Please take a minute to log into Editorial Manager at http://www.editorialmanager.com/pbiology/, click the "Update My Information" link at the top of the page, and update your user information to ensure an efficient production process.

PRESS

We frequently collaborate with press offices. If your institution or institutions have a press office, please notify them about your upcoming paper at this point, to enable them to help maximise its impact. If the press office is planning to promote your findings, we would be grateful if they could coordinate with biologypress@plos.org. If you have previously opted in to the early version process, we ask that you notify us immediately of any press plans so that we may opt out on your behalf.

We also ask that you take this opportunity to read our Embargo Policy regarding the discussion, promotion and media coverage of work that is yet to be published by PLOS. As your manuscript is not yet published, it is bound by the conditions of our Embargo Policy. Please be aware that this policy is in place both to ensure that any press coverage of your article is fully substantiated and to provide a direct link between such coverage and the published work. For full details of our Embargo Policy, please visit http://www.plos.org/about/media-inquiries/embargo-policy/.

Thank you again for choosing PLOS Biology for publication and supporting Open Access publishing. We look forward to publishing your study.

Best wishes,

Richard

Richard Hodge, PhD

Senior Editor, PLOS Biology

rhodge@plos.org

PLOS

Empowering researchers to transform science

Carlyle House, Carlyle Road, Cambridge, CB4 3DN, United Kingdom

California (U.S.) corporation #C2354500, based in San Francisco
